# Incidence of and risk factors for lumbar disc herniation with radiculopathy in adults: a systematic review

**DOI:** 10.1007/s00586-024-08528-8

**Published:** 2024-10-25

**Authors:** Cesar A. Hincapié, Daniela Kroismayr, Léonie Hofstetter, Astrid Kurmann, Carol Cancelliere, Y. Raja Rampersaud, Eleanor Boyle, George A. Tomlinson, Alejandro R. Jadad, Jan Hartvigsen, Pierre Côté, J. David Cassidy

**Affiliations:** 1 EBPI-UWZH Musculoskeletal Epidemiology Research Group, University of Zurich and Balgrist University Hospital, Zurich, Switzerland; 2 Epidemiology, Biostatistics and Prevention Institute (EBPI), University of Zurich, Zurich, Switzerland; 3 University Spine Centre Zurich (UWZH), Balgrist University Hospital, University of Zurich, Zurich, Switzerland; 4 Institute for Disability and Rehabilitation Research and Faculty of Health Sciences, Ontario Tech University, Oshawa, Canada; 5 Schroeder Arthritis Institute, Division of Orthopaedic Surgery, Toronto Western Hospital, University Health Network, Toronto, Canada; 6 Thunderbird Partnership Foundation, London, Canada; 7 Division of Epidemiology, Dalla Lana School of Public Health, University of Toronto, Toronto, Canada; 8 Toronto General Research Institute, University Health Network, Toronto, Canada; 9 Vivenxia Group LLD, Los Angeles, USA; 10 Center for Muscle and Joint Health, Department of Sports Science and Clinical Biomechanics, University of Southern Denmark, Odense, Denmark; 11 Chiropractic Knowledge Hub, Odense, Denmark

**Keywords:** Radiculopathy, Back pain, Incidence, Risk factors, Systematic review

## Abstract

**Background:**

Lumbar disc herniation (LDH) with radiculopathy is associated with greater pain, disability, healthcare use, and costs compared with nonspecific low back pain. Reliable information about its incidence and risk factors were lacking.

**Questions:**

(1) What is the incidence of lumbar disc herniation (LDH) with radiculopathy in adults? (2) What are the risk factors for LDH with radiculopathy in adults?

**Methods:**

Systematic review. We searched five electronic databases from 1970 to September 2023. Eligible cohort and case–control studies were identified and independently assessed for risk of bias. A qualitative best evidence synthesis of low and moderate risk of bias studies was conducted.

**Results:**

We critically reviewed 87 studies and synthesised data from 59 (68%) studies; 12 were of low and 47 of moderate risk of bias. The lower and upper bound limits of the 95% CIs of annual incidence estimates ranged from 0.3 to 2.7 per 1000 persons for surgical case definitions, from 0.04 to 1.5 per 1,000 persons for hospital-based case definitions, and from 0.1 to 298.3 per 1,000 persons for clinical case definitions. Factors associated with the development of LDH with radiculopathy included middle-age (30–50 years), smoking, higher BMI, presence of cardiovascular risk factors (in women), and greater cumulative occupational lumbar load by forward bending postures and manual materials handling, with effect sizes ranging from ranging from 1.1 (1.0–1.3) to 3.7 (2.3–6.0).

**Conclusions:**

Incidence of LDH varies in different populations and according to case definition. Risk factors include individual, behavioural, and work-related variables. Our findings support the need to develop standardised case definitions that validly classify the clinical spectrum of LDH and for future low risk of bias studies examining causal relationships for LDH with radiculopathy in adults.

**Supplementary Information:**

The online version contains supplementary material available at 10.1007/s00586-024-08528-8.

## Introduction

Low back pain is the leading cause of disability globally and associated with a large burden on persons, healthcare systems, and society [1]. Radiculopathy due to lumbar disc herniation (LDH) is one of the most recognizable disorders of the low back. The diagnosis is typically based on a combination of symptoms and signs suggesting lumbar spinal nerve root compression or irritation, such as radicular pain with nerve root tension signs, neurologic deficits, and imaging findings that correlate with the clinical syndrome [2, 3]. LDH, defined as the localised displacement of disc material beyond the margins of the intervertebral disc space [4], is the most common cause of lumbosacral radiculopathy [5]. Compared with nonspecific low back pain without radiculopathy, LDH with radiculopathy is typically associated with greater pain, disability, healthcare use, and intervention [6–9].

Previous studies of symptomatic LDH have reported a point prevalence of about 5% in adults 30 years of age and older [10, 11], varying by sex and age. In people aged 25 to 55 years, 95% of symptomatic herniated discs occur at the lower lumbar spine (L4-L5 and L5-S1 levels) [12]. However, little is known about the incidence of LDH with radiculopathy, and consequently, risk factors are not well understood. Our aim was to synthesise the evidence on the incidence of and risk factors for LDH with radiculopathy in adults. To the best of our knowledge, no recent systematic review has focused on synthesising the best available evidence on the incidence and risk factors of LDH with radiculopathy.

Therefore, the specific research questions for this systematic review were:

1. What is the incidence of lumbar disc herniation (LDH) with radiculopathy in adults?
2. What are the risk factors for LDH with radiculopathy in adults?

## Methods

Our review was conducted in accordance with the Preferred Reporting Items for Systematic Reviews and Meta-Analyses (PRISMA) 2020 statement [13]. Details of our protocol were registered in the international prospective register of systematic reviews (PROSPERO No. CRD42011001197) [14]. This review was initially conducted in partial fulfilment of a doctoral dissertation in epidemiology as an unpublished manuscript [15], which we updated to September 2023.

### Data sources and search strategy

We systematically searched Medline, Embase, Cochrane Central Trials Registry, Cochrane Database of Systematic Reviews, and Database of Abstracts Related to Effects; initially from 1970 to March 2016. In February 2022, we conducted an update and identified records published between April 2016 and January 2022. We ran a final search update to 21 September 2023. Search strategies combined terms from three key concepts: *intervertebral disc herniation*, *lumbar spine*, and *etiology*, and were developed in consultation with an experienced information specialist (Appendix 1 in Online Supplementary Material). The reference lists of all eligible studies were also searched for additional relevant studies.

### Eligibility criteria

Eligible were studies published in English, French, or Spanish that used cohort, case–control, or randomised trial (only potentially eligible for risk factor evidence) designs to examine the incidence of and/or risk factors for LDH with radiculopathy in adults (aged 18 years or older). We excluded studies of asymptomatic LDH, or LDH due to neoplasm, infection, fracture, dislocation, or spinal cord injury. Narrative reviews, case reports or series, cross-sectional, cadaveric, animal studies, or studies where the majority of the population was < 18 years of age were also excluded.

In consultation with clinical experts, we defined LDH with radiculopathy as lumbar or lumbosacral radiculopathy, sciatica, and/or clinically relevant neurologic deficit, with or without advanced imaging (i.e., MRI or CT) confirmation of disc herniation. We conceptualised and applied three types of case definitions for LDH with radiculopathy. A surgical case definition was used for study populations that had surgical intervention for LDH with radiculopathy. A hospital-based case definition applied when study participants sought medical care for LDH in a hospital setting without surgical intervention. A clinical case definition of LDH with radiculopathy was used to describe study populations identified or selected based on relevant clinical signs and symptoms of LDH with radiculopathy in primary care or community-based settings.

### Study selection

A three-phase screening process was used to identify eligible studies for our review. In the first-phase title/abstract screen, one of two reviewers (CAH/DK) rated all records as *relevant*, *irrelevant*, or of *uncertain relevance*. In the second-phase title/abstract screen of those records rated relevant or uncertain from the first-phase, six reviewers (CAH, JDC, PC, DK, LH, AK) independently identified *potentially eligible* reports to be retrieved for full-text screening. In the third-phase full-text screening, potentially eligible reports were deemed either eligible or ineligible by two independent reviewers (CAH, DK, JDC, PC). When necessary, discrepancies were resolved by consensus. Reasons for exclusions of full texts and references to the excluded reports are available in Appendix 2 (Online Supplementary Material).

### Risk of bias assessment

We assessed eligible reports for risk of bias in teams of two independent reviewers (CAH, DK, JDC, PC, ARJ, YRR, EB, JH, LH, AK) using checklists (cohort, case–control, and controlled trials) based on criteria recommended by the Scottish Intercollegiate Guidelines Network (SIGN) [16]. Reviewers summarised judgments to assess overall study-level risk of bias as low, moderate, or high for all studies. When further information was required from original authors, up to three attempts were made to contact them by email. We used consensus and arbitration by a third reviewer, when necessary, to resolve discrepancies.

### Data extraction, analysis, and synthesis

One reviewer (CAH/DK) extracted (verified by a second reviewer [DK/CAH]) the following data from eligible studies: study and population characteristics, case definitions and study outcomes, risk factors considered, and estimates of incidence and risk. Any discrepancies were resolved by consensus or with a third reviewer when necessary.

Based on the principle of best evidence synthesis [17], we conceptualised low and moderate risk of bias studies as admissible and the focus of our qualitative synthesis giving more weight to low risk of bias studies, but also tabled high risk of bias studies to report their findings. In best evidence synthesis, low and moderate risk of bias studies—deemed to be less susceptible to biased findings based on methodological adequacy—are given more weight than studies judged to be high risk of bias [18, 19]

Risk factor evidence was prioritised using the framework described by Côté et al. [20] Phase I studies (exploratory) are hypothesis generating and describe crude associations without considering confounding. Phase II studies (exploratory) focus on sets of risk factors or examine which risk factors are associated with the development of LDH with radiculopathy without an explicit attempt to assess and control for confounding. Phase III studies (confirmatory) are investigations of explicit hypotheses that allow for the quantification of the strength, direction, and independence of a proposed causal relationship between a risk factor and the development of LDH with radiculopathy. We combined studies with different research questions examining the same source population. If phases of evidence varied, we tabled them as mixed (Phase I/II or Phase II/III). Phase III studies are considered the strongest evidence for risk factors, followed by phase II, and then phase I studies, which are considered more preliminary evidence.

We searched for all risk factors in any domain, such as sociodemographic, health conditions, health behaviours, personal physical and psychosocial, and occupational physical and psychosocial risk factors.

We reported annual incidence estimates (cohort studies only) and risk factor effect estimates (only cohort and case–control studies, as no randomised trials were included) with 95% confidence intervals (CIs) as reported in original studies or calculated from raw data presented in original studies. Risk factor effect estimates were reported as odds ratio (OR), risk ratio (RR), or hazard rate ratio (HR), as appropriate. When needed, we computed these statistics in R [21] using standard methods [22].

## Results

### Literature search and study selection

**Fig. 1:**
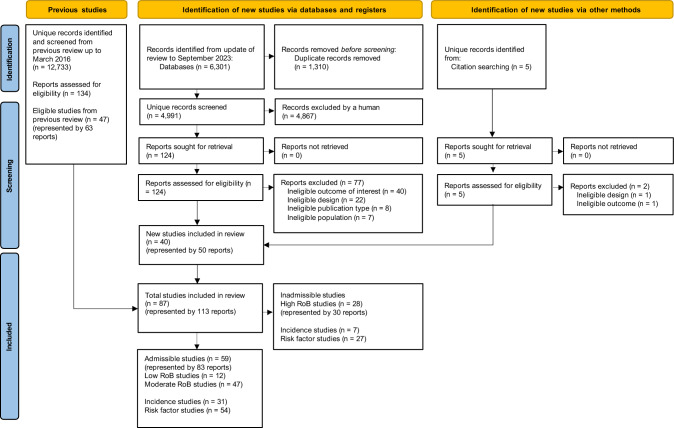
Flow diagram of information through phases of the systematic review. Abbreviations: LDH, lumbar disc herniation; ROB, risk of bias

Our search yielded 17,729 unique citations, of which 263 studies underwent full-text screening. 87 studies (represented by 113 reports) met our eligibility criteria and were critically appraised. Of these, we rated 59 studies (68%) as admissible (low or moderate risk of bias) for our best evidence synthesis, and 28 studies (32%) as inadmissible due to high risk of bias (Fig. 1).

### Study characteristics

Table 1 provides a quantitative summary of the 87 eligible studies, while Table 2 details their summary characteristics. Overall, 12 studies (14%) were rated low risk of bias; 47 studies (54%) moderate risk of bias; and 28 studies (32%) high risk of bias. The most common sources of bias included poor or inadequate description of sampling methods, exposure measurement, and confounding consideration (see Appendix 3 in Online Supplementary Material for summary risk of bias ratings). The 59 admissible studies consisted of 31 cohort studies and 28 case–control studies. Appendix 4 (Online Supplementary Material) provides full descriptions of the 31 admissible studies that reported on incidence [23–58]. Appendix 5 (Online Supplementary Material) details the 54 admissible studies that reported on risk factors [23–25, 27–32, 34–45, 47–56, 58–104]. Appendices 6 and 7 (Online Supplementary Material) characterise the 28 high risk of bias studies deemed inadmissible.

**Table 1:** Summary of studies examining the incidence or risk factors of LDH with radiculopathy in adults

		Studies by admissibility*		Admissible studies by focus†	
Characteristic	All studies (n = 87)	Admissible (n = 59)	Inadmissible (n = 28)	Incidence (n = 31)	Risk (n = 54)
Risk of bias—n (%)					
Low	12 (14)	12 (20)	NA	12 (39)	10 (19)
Moderate	47 (54)	47 (80)	NA	19 (61)	44 (81)
High	28 (32)	NA	28 (100)	NA	NA
Focus—n (%)					
Incidence only	6 (7)	5 (8)	1 (4)	5 (16)	NA
Risk only	49 (56)	28 (47)	21 (75)	NA	28 (52)
Both incidence and risk	32 (37)	26 (44)	6 (21)	26 (84)	26 (48)
Study design—n (%)					
Case–control	48 (55)	28 (47)	20 (71)	NA	28 (52)
Cohort	39 (45)	31 (53)	8 (29)	31 (100)	26 (48)
Case definition type—n (%)					
Surgical	15 (17)	8 (14)	7 (25)	6 (19)	7 (13)
Hospital	20 (23)	13 (22)	7 (25)	11 (35)	10 (19)
Clinical	43 (49)	33 (56)	10 (36)	12 (39)	32 (59)
Mixed surgical/hospital/clinical	9 (10)	5 (8)	4 (14)	2 (6)	5 (9)
Population source—n (%)					
General	15 (17)	11 (19)	4 (14)	11 (35)	8 (15)
Occupational	24 (28)	17 (29)	7 (25)	17 (55)	15 (28)
Healthcare	48 (55)	31 (53)	17 (61)	3 (10)	31 (57)
Sex—n (%)					
Men only	16 (18)	11 (19)	5 (18)	9 (29)	9 (17)
Women only	3 (3)	3 (5)	0	3 (10)	3 (6)
Both men and women	68 (78)	45 (76)	23 (82)	19 (61)	42 (78)
Incidence estimate type‡—n (%)	(n = 38)	(n = 31)	(n = 7)	(n = 31)	
Cumulative incidence only	22 (58)	19 (63)	3 (43)	19 (61)	NA
Incidence density only	12 (32)	10 (32)	2 (29)	10 (32)	NA
Both estimate types	3 (8)	2 (6)	1 (14)	2 (6)	NA
NR	1 (3)	0 (0)	1 (14)	0 (0)	NA
Risk factors considered§—n (%)	(n = 81)	(n = 54)	(n = 27)		(n = 54)
Sociodemographic	40 (49)	28 (52)	12 (44)	NA	28 (52)
BMI, anthropometrics, genetics	45 (56)	33 (59)	12 (44)	NA	33 (59)
Health conditions, prior pain, comorbidities	28 (35)	17 (30)	11 (41)	NA	17 (30)
Health behaviours	31 (38)	21 (39)	10 (37)	NA	21 (39)
Personal—physical	10 (12)	7 (13)	3 (11)	NA	7 (13)
Personal—psychosocial	9 (11)	9 (17)	0	NA	9 (17)
Occupational—physical	25 (31)	18 (33)	7 (26)	NA	18 (33)
Occupational—psychosocial	8 (10)	6 (11)	2 (7)	NA	6 (11)
Phase of risk factor evidence||—n (%)	(n = 87)	(n = 59)	(n = 28)		(n = 54)
I	27 (31)	14 (24)	13 (46)	NA	14 (26)
II	43 (49)	31 (53)	12 (43)	NA	31 (57)
III	7 (8)	7 (12)	0	NA	7 (13)
Mixed (I/II or II/III)	4 (5)	2 (3)	2 (7)	NA	2 (4)
NA	6 (7)	5 (8)	1 (4)	NA	0

NA, not applicable; NR, not reported

^*^Admissible studies were those rated as low or moderate risk of bias; inadmissible studies were those rated as high risk of bias

^†^Categories are not mutually exclusive

^‡^Proportions among studies on incidence; denominators indicated in header row

^§^Proportions among studies on risk factors and not mutually exclusive; denominators indicated in header row

|| Hierarchical categories of risk factor evidence: phase I and II is exploratory evidence for an association, phase III is confirmatory evidence from better quality studies

**Table 2:** Characteristics of eligible studies examining the incidence or risk factors of LDH with radiculopathy in adults

First author, Year published Focus	Country	Study design	Source population	Participants and setting Follow-up	Age (mean)	N (% female); Participation %	Study outcome	Case definition type	ROB	Phase
Burske-Hohlfeld 1990 [10] Incidence	USA	Cohort	General	All LDH surgeries of Olmsted County residents Follow-up: 30y	15-78y (42y)	1,028 (NR); 88% incident surgery	LDH surgery	Surgical	Low	NA
Mattila 2009 [95] Incidence	Finland	Cohort	Occupational	Male military conscripts linked to the National Hospital Discharge Register Follow-up: 267,700 person-years	18-29y (20y)	387,070 (0%); 100%	Hospitalised LDH	Hospital	Low	NA
Jhawar 2006 [60] Incidence and risk	USA	Cohort	Occupational	Female nurses from the Nurses’ Health Study responding to a questionnaire Follow-up: 16y	30-55y in 1976 (NR)	98,407 (100%); ≥ 85%	Clinical LDH	Clinical	Low	III
Hincapié 2018 [48] Incidence and risk	Canada	Cohort with self-controlled case series analysis	General	All adults with acute LDH requiring ED visit and early surgery from April 1994 to December 2004, using population-based Ontario healthcare databases Follow-up: 11y, > 100′000′000 person-years	≥ 18y (43y)	195 (40%)	LDH surgery	Surgical	Low	III
Wahlström 2018 [134] Incidence and risk	Sweden	Cohort	Occupational	Male construction workers who participated in a national occupational health surveillance program Follow-up: 1-32y	20-65y (NR)	288,926 (0%)	Hospitalised LDH	Hospital	Low	III
Balling 2019 [4] Incidence and risk	Denmark	Cohort	General	Nationwide cohort linked to the Danish Health Examination Survey	18-99y (48y)	46,826 (60%)	Hospitalised LDH or sciatica	Hospital	Low	III
Brauer 2020 [9] Incidence and risk	Denmark	Cohort	Occupational	Copenhagen Airport Cohort baggage handlers linked to the National Patient Register and Civil Registration System Follow-up: 22y	NR	68,436 (0%)	Hospitalised LDH or LDH surgery	Hospital	Low	III
Heliövaara 1987 [38, 39, 41] Incidence and risk	Finland	Cohort with case–control risk analyses	General	Nationwide cohort linked to the National Hospital Discharge Register Follow-up: 11y	≥ 15y (NR)	57,000 (48%); 83%	Hospitalised LDH or sciatica	Hospital	Low	II
Zitting 1998 [27] Incidence and risk	Finland	Cohort	General	Birth cohort from 2 provinces linked to the National Hospital Discharge Register Follow-up: 28y	15-28y among cases (NR)	12,058 (NR); 92%	Hospitalised LDH	Hospital	Low	II
Miranda 2002 [97] Incidence and risk	Finland	Cohort	Occupational	Forest industry workers responding to a questionnaire Follow-up: 1y	NR (45y)	2,077 (26%); 77%	Sciatica	Clinical	Low	II
Mattila 2008 [94] Incidence and risk	Finland	Cohort	General	Nationwide adolescent cohort linked to the National Hospital Discharge Register Follow-up: 651,027 person-years	15-41y among cases (27y at surgery)	57,408 (54%); 79%	LDH surgery	Surgical	Low	II
Jung 2020 [67] Incidence and risk	South Korea	Cohort	General	Nationwide cohort of Korean residents linked to the National Health Insurance Service Follow-up: 9y	NR (NR)	NR (NR)	Hospitalised LDH	Hospital	Low	II
Heikkilä 1989 [37] Incidence	Finland	Cohort	General	Finnish adult twin cohort of the same sex linked to the National Hospital Discharge Register Follow-up: 14y	≥ 24y (NR)	18,730 (55%); 100%	Hospitalised sciatica	Hospital	Mod	NA
Bovenzi 2015 [8] Incidence	Italy	Cohort	Occupational	Male professional drivers employed in several industries and public utilities in various provinces of Italy Follow-up: 1-2y	NR (41y)	598 (0%); 90%	Clinical LDH	Clinical	Mod	NA
Jäntti 2022 [58] Incidence	Finland	Cohort	General	Patients presenting to the emergency department in the year 2020 Follow-up: 1y	NR (54y)	4,310 (55%)	Hospital based LDH	Hospital	Mod	NA
Seidler 2003 [118] Risk	Germany	Case–control	Healthcare	Cases: 225 male patients with acute LDH from Frankfurt/Main Controls: 107 population and 90 hospital controls Follow-up: NA	25-65y (42y)	437 (0%); 66–93%	Clinical LDH	Clinical	Mod	III
Huang, 2019 [53] Incidence and Risk	Taiwan	Cohort	Healthcare	Nationwide cohort of healthcare professionals linked to the National Health Insurance Research Database Follow-up: 5y	NR (43y)	165,600 (36%)	Clinical LDH	Clinical	Mod	III
^a^ Leino-Arjas 2004 [86] ^b^ Leino-Arjas 2002 [87] Incidence and risk	Finland	Cohort	Occupational	Nationwide workforce linked to the National Hospital Discharge Register Follow-up: 1y	^a^ 25-64y (NR) ^b^ 20-64y (NR)	^a^ 1,783,616 (51%); 100% ^b^ 2,409,319 (NR); 100%	Hospitalised LDH and LDH surgery	Hospital and surgical	Mod	^a^ III ^b^ II
^a^ Seidler 2009 [117] ^b^ Bergmann 2017 [5] ^c^ Schumann 2010 [115] ^d^ Seidler 2011 [119] Risk	Germany	Case–control	Healthcare	^a,b,c,d^ Cases: 915 patients with structural lumbar disc diseases (564 with LDH) in 4 regions of Germany ^a,c,d^ Controls: 901 population controls ^b^ Controls: 655 residents from the same catchment area Follow-up: NA	^a,c,d^ 25-70y (48y) ^b^ 25-70y (51y among cases)	^a,c,d^ 1,816 (50%); 53–66% ^b^ 1,570 (51%)	LDH hospital treatment	Hospital	Mod	^a, b^ III ^c, d^ II
Kelsey 1975 [70, 71, 74–76] Risk	USA	Case–control	Healthcare	Cases: 223 radiology and hospital patients with LDH, 1971–1973 Controls: 217 controls matched on age, sex, and medical setting; 494 unmatched controls Follow-up: NA	20-64y (39y among cases)	934 (41%); 78%	Combined clinical, hospitalised and surgical LDH	Clinical, hospital and surgical	Mod	II
Kelsey 1984 [72, 73] Risk	USA	Case–control	Healthcare	Cases: 325 orthopaedic, neurosurgical, and hospital patients with LDH, 1979–1981 Controls: 241 controls matched on age, sex, and medical setting Follow-up: NA	20-64y (NR)	566 (45%); 72–79%	Combined clinical, hospitalised and surgical LDH	Clinical, hospital and surgical	Mod	II
Riihimäki 1989 [111] Incidence and risk	Finland	Cohort	Occupational	Male concrete reinforcement workers and house painters responding to a questionnaire Follow-up: 5y	25-54y at baseline (NR)	178 (0%); 77–80%	Sciatica	Clinical	Mod	II
Mundt 1993 [99, 100] Risk	USA	Case–control	Healthcare	Cases: 297 orthopaedic, neurosurgical, and hospital patients with LDH, 1986–1988 Controls: 287 clinical and hospital controls Follow-up: NA	20-64y (NR)	585 (41%); 76–79%	Combined clinical, hospitalised and surgical LDH	Clinical, hospital and surgical	Mod	II
Jørgensen 1994 [66] Incidence and risk	Denmark	Cohort	Occupational	Assistant nurses and all females linked to the Danish National Registry of Hospitalised Patients Follow-up: 1y	20-69y (NR)	1,681,152 (100%); 100%	LDH surgery	Surgical	Mod	II
Riihimäki 1994 [110] Pietri-Taleb 1995 [108] Incidence and risk	Finland	Cohort	Occupational	Male machine operators, carpenters and office workers responding to a questionnaire Follow-up: 3y	25-49y (37y)	1,149 (0%); 83%	Sciatica	Clinical	Mod	II
Leclerc 2003 [83] Incidence and risk	France	Cohort	Occupational	Male workers in the national electricity and gas company responding to a questionnaire Follow-up: 2y	40-50y at baseline (NR)	841 (0%); 65%	Sciatica	Clinical	Mod	II
^a^ Jarvik 2005 [59] ^b^ Suri 2014 [127] Incidence and risk	USA	Cohort	Healthcare	Veterans Affairs outpatients at the Puget Sound Health Care System, Seattle Division Follow-up: 3y	35-70y (median, 53y)	148 (13%); 89%	Clinical LDH and sciatica	Clinical	Mod	II
^a^ Sørensen 2011 [123] ^b^ Jørgensen 2013 [65] Incidence and risk	Denmark	Cohort	Occupational	Male workers in 1970 to 1971, at 14 private and public companies (railway, telephone, insurance, postal and firefighting) in Copenhagen, linked to Danish National Hospital Register Follow-up: 6-33y	40-59y (NR)	3,833 (0%); 87%	Hospitalised LDH	Hospital	Mod	II
Wahlström 2012 [135] Incidence and risk	Sweden	Cohort	Occupational	Male construction workers who participated in a national occupational health surveillance program Follow-up: 1-32y	20-65y (NR)	263,529 (0%)	Hospitalised LDH	Hospital	Mod	II
Zhang 2016 [ 79] Risk	China	Case–control	Healthcare	Cases: 396 patients with single-level LDH surgery, from 2013–2014 Controls: 394 age and sex matched controls Follow-up: NA	18-82y (42y)	790 (42%)	LDH surgery	Surgical	Mod	II
Bjornsdottir 2017 [6] Risk	Iceland	Case–control	Healthcare	Cases: 4,748 patients with LDH surgery, 1997–2015 Controls: 282,590 controls without diagnosis of LDH Follow-up: NA	NR (45y among cases)	287,338 (NR)	LDH surgery	Surgical	Mod	II
Chan 2018 [12] Incidence and Risk	Taiwan	Cohort	Occupational	Physicians and Non-Physicians linked to the Taiwan National Health Research Database Follow-up: 5y	NR (47y)	115,488 (42%)	Clinical LDH	Clinical	Mod	II
Dong 2018 [20] Zhu 2018 [84] Risk	China	Case–Control	Healthcare	Cases: 380 patients with clinical LDH, 2015–2019 Controls: 692 unrelated healthy controls Follow-up: NA	≥ 18y (49y)	1072 (42%)	Clinical LDH and sciatica	Clinical	Mod	II
Fouquet 2018 [27] Incidence and risk	France	Cohort	Occupational	^a^ LDS: French workers with LDH surgery ^b^ OD-DRS: French workers compensated for disc related sciatica Follow-up: 2y	20-59y	^a^ 1,489 (NR) ^b^ 1,009 (NR	^a^ LDH surgery ^b^ Clinical LDH and sciatica	^a^ Surgical ^b^ Clinical	Mod	II
Han 2018 [35] Incidence and Risk	South Korea	Cohort	Occupational	Nationwide cohort of public officers linked to the National Health Insurance Service Follow-up: 12y	NR (40y)	860,221 (36%)	Clinical LDH	Clinical	Mod	II
Jing 2018 [63] Risk	China	Case–control	Healthcare	Cases: 845 patients with LDH Controls: 1,751 healthy controls Follow-up: NA	32-62y (48y)	2,596 (31%)	Clinical LDH	Clinical	Mod	II
Kim 2018 [79] Incidence and risk	South Korea	Cohort	General	Nationwide cohort of South Korean residents linked to the National Health Insurance Service Follow-up: 7y	20-69y (NR)	18,786,256 (33%)	Clinical LDH	Clinical	Mod	II
Knox 2018 [80] Incidence and risk	USA	Cohort	Occupational	Military helicopter pilots and active-duty military service members linked to the Defense Medical Epidemiology Database Follow-up: 10y	≥ 20y (NR)	NR (NR)	First-time LDH diagnosis	Clinical	Mod	II
Li 2018 [89] Risk	China	Case–control	Healthcare	Cases: 120 patients with LDH, from 2015 Controls: 120 controls without LDH Follow-up: NA	≥ 18y (55y)	240 (30%)	Clinical LDH	Clinical	Mod	II
Hu 2019 [51] Ji 2019 [61] Liu 2020 [91] Wu 2020 [140] Yang 2020 [144] ^a^ Hu 2022 [52] ^b^ Han 2023 [36] ^c^ Wu 2023 [141] Risk	China	Case–control	Healthcare	Cases: 508 clinical LDH patients, from 2015–2017 Controls: 508 age and sex matched controls from the health check-up ^a^504 cases, 503 controls ^b^509 cases, 510 controls ^c^504 cases, 500 controls Follow-up: NA	NR (49y)	1,016 (42%) ^a^1,007 (42%) ^b^1,019 (42%) ^c^1,004 (42%)	Clinical LDH and sciatica	Clinical	Mod	II
Yang 2019 [143] Risk	China	Case–control	Healthcare	Cases: 380 patients with LDH Controls: 400 unrelated healthy controls Follow-up: NA	NR (51y)	780 (41%)	Clinical LDH	Clinical	Mod	II
Zhu 2019 [96] Tai 2020 [129] Risk	China	Case–control	Healthcare	Cases: 498 LDH patients Controls: 463 healthy unrelated controls Follow-up: NA	≥ 18y (50y)	961(41%)	Clinical LDH and sciatica	Clinical	Mod	II
Luo 2020 [92] Risk	China	Case–control	Healthcare	Cases: 231 patients with LDH recruited from 2012–2018 Controls: 312 matched healthy controls Follow-up: NA	25-84y (46y)	543 (40%)	Clinical LDH	Clinical	Mod	II
Fidan 2022 [26] Risk	Turkey	Case–control	Healthcare	Cases: 651 patients with LDH recruited from January 2021 till August 2021 Controls: 651 age and sex matched patients with LBP without LDH Follow-up: NA	18-65y (43y)	1,302 (63%)	Clinical LDH	Clinical	Mod	II
Nyrhi 2023 [103] Incidence and risk	Finland	Cohort	General	Nationwide cohort of Finnish women from the Finnish Care Register fir Health and Finnish Medical Birth Register from 1999 and 2017 Follow-up: 19y	15-49y (NR)	13,912 (100%)	LDH surgery	Surgical	Mod	II
Hurme 1983 [55] Incidence and risk	Finland	Cohort	Healthcare	AII LDH surgeries from surgical registers of the Turku University Central Hospital area Follow-up: 5y	15-80y among cases (42y at surgery)	1,011 surgeries (44%); 79% incident surgery	LDH surgery	Surgical	Mod	I
Noponen-Hietela 2005 [102] Risk	Finland	Case–control	Healthcare	Cases: 155 unrelated Finnish patients with sciatica from the Oulu University Hospital area, 1997–1998 Controls: 179 unrelated University of Oulu employees and students (all Finnish) Follow-up: NA	Cases: 19-78y (44y) Controls: 20-69y (39y)	334 (39% among cases, 69% among controls); NR	Sciatica	Clinical	Mod	I
Mio 2007 [96] Risk	Japan	Case–control	Healthcare	Cases: 823 hospital patients of Japanese origin with LDH Controls: 841 hospital controls of Japanese origin Follow-up: NA	Cases: 11-83y (36y) Controls: 13-87y (61y)	1,664 (41% among cases, 63% among controls; NR	Clinical LDH	Clinical	Mod	I
Virtanen 2007 [133] Risk	Finland	Case–control	Healthcare	Cases: 243 unrelated Finnish patients with sciatica from the Oulu University Hospital area Controls: 259 unrelated Finnish persons from the same catchment area Follow-up: NA	NR (NR)	502 (45%); NR	Sciatica	Clinical	Mod	I
Hirose 2008 [49] Risk	Japan	Case–control	Healthcare	Cases: 847 hospital patients of Japanese origin with LDH, 2001–2007 Controls: 896 Japanese persons from the same catchment area Follow-up: NA	Cases: NR (39y) Controls: NR (62y)	1,743 (38%); NR	Clinical LDH	Clinical	Mod	I
Karasugi 2009 [69] Risk	Japan and Finland	Case–control	Healthcare	Japan Cases: 862 hospital patients of Japanese origin with LDH, 2001–2007 Controls: 896 Japanese persons from the same catchment area Finland Cases: 257 unrelated Finnish patients with sciatica from the Oulu University Hospital area Controls: 249 unrelated Finnish persons from the same area Follow-up: NA	Japan Cases: NR (39y) Controls: NR (62y) Finland NR (NR)	Japan 1,758 (38%); NR Finland 506 (NR); NR	Clinical LDH and sciatica	Clinical	Mod	I
Cong 2010 [16] Risk	China	Case–control	Healthcare	Cases: 70 male hospital patients of Chinese Han origin with LDH Controls: 14 male spinal trauma controls and 113 male healthy blood donor controls Follow-up: NA	Cases: 14-41y (33y) Controls: 20-49y (38y)	197 (0%); NR	Clinical LDH	Clinical	Mod	I
Mu 2014 [98] Risk	China	Case–control	Healthcare	Cases: 231 Han Chinese patients with LDH, from 2011–2012 Controls: 770 Han Chinese patients who never experienced symptoms suggesting LDH Follow-up: NA	NR (48y)	601 (45%)	Clinical LDH	Clinical	Mod	I
Huang 2017 [54] Risk	China	Case–control	Healthcare	Cases: 267 patients with clinical LDH, from 2014–2015 Controls: 300 healthy control from the same geographical location Follow-up: NA	18-60y (44y)	567 (39%)	Clinical LDH	Clinical	Mod	I
Jiang 2017 [62] Risk	China	Case–control	Healthcare	Cases: 156 LDH patients, from 2012–2016 Controls: 400 controls without LDH Follow-up: NA	NR (45y)	556 (45%)	Clinical LDH	Clinical	Mod	I
Ghandhari 2018 [33] Risk	Iran	Case–control	Healthcare	Cases: 129 patients with LDH and signs of radiculopathy Controls: 61 controls without LBP or radiculopathy Follow-up: NA	NR (41y)	190 (37%)	Clinical LDH	Clinical	Mod	I
Wang 2018 [137] Risk	China	Case–control	Healthcare	Cases: 134 LDH patients who received treatment, from 2010–2015 Controls: 100 healthy controls matched for age, sex, BMI and occupation Follow-up: NA	18-39y (30y)	234 (35%)	Hospitalised LDH	Hospital	Mod	I
Withanage 2018 [139] Risk	Sri Lanka	Case–control	Healthcare	Cases: 51 patients with LDH from the district of Colombo Controls: 68 controls from several districts of Sri Lanka Follow-up: NA	18-74y (42y)	119 (52%)	Clinical LDH	Clinical	Mod	I
Zhou 2018 [87] Risk	China	Case–control	Healthcare	Cases: 53 LDH patients Controls: 129 controls Follow-up: NA	18-35y (28y)	182 (42%)	Clinical LDH	Clinical	Mod	I
Makovicka 2019 [93] Incidence	USA	Cohort	Occupational	Collegiate football players followed with NCAA Injury Surveillance Program Follow-up: 5y	NR (NR)	NR (NR)	Clinical LDH and sciatica	Clinical	High	NA
Hrubec 1975 [50] Risk	USA	Case–control	Occupational	Cases: 1,132 first admission records to Army hospitals for LDH, 1944–1945 Controls: 1,095 records of Army National Service Life Insurance policyholders matched on age and period of military service Follow-up: NA	18-56y (NR)	1,095 case–control pairs (0%); 97%	Hospitalised LDH	Hospital	High	II
Bongers 1988 [7] Incidence and risk	The Netherlands	Cohort	Occupational	Disability pension due to LDH in male crane workers and floor workers at a steel company Follow-up: 10y	NR (NR)	1,405 (0%); 71%	Disability pension due to LDH	Clinical	High	II
Chibnall 2006 [14] Risk	USA	Cohort	Occupational	African American and non-Hispanic white workers’ compensation claimants who filed low back injury claims Follow-up: NA	18-55y (NR)	2,934 (38%); 50%	Clinical and surgical LDH	Clinical and surgical	High	II
Zhang 2009 [87] Risk	China	Case–control	Healthcare	Cases: 2,010 orthopaedic hospital patients with LDH, 2005–2007 Controls: 2,170 randomly selected hospital controls matched on race, sex, age, and living area Follow-up: NA	NR (46y)	4,180 (40%); NR	Clinical LDH	Clinical	High	II
Rivinoja 2011 [112] Incidence and risk	Finland	Cohort	General	1966 Northern Finland Birth Cohort linked to the Finnish Hospital Discharge Register Follow-up: 28y	NR (NR)	9,016 (50%)	Hospitalised sciatica and LDH surgery	Hospital and surgical	High	II
Roquelaure 2011 [113] Incidence and risk	France	Cohort	General	All residents of the Loire-Atlantique region discharged in 2002–2003 following LDH surgery Follow-up: 2y	20-59y (42y)	272 (56%); 49–60%	Surgical LDH	Surgical	High	II
Chung 2013 [15] Incidence and risk	Taiwan	Cohort	Occupational	Nurses and referent participants from the Taiwanese National Health Insurance Research Database between 2004–2010 Follow-up: 7y	NR (34y for women, 31y for men)	Nurses: 3,914 (99%) Referents: 11,744 (99%); NR	Hospitalised LDH	Hospital	High	II
Zhang 2013 [114] Risk	China	Case–control	Healthcare	Cases: Orthopaedic and spine surgery hospital patients with LDH, 2005–2007 Controls: Hospital inpatients or participants of medical exams matched for race, sex, age, living location Follow-up: NA	Cases: NR (46y) Controls: NR (47y)	268 (38%); 97%	Hospitalised LDH	Hospital	High	II
Chiang 2014 [13] Risk	China	Case–control	Healthcare	Cases: Hospital patients with LDH Controls: Hospital patients without LDH from a medical or surgery department Follow-up: NA	Cases: NR (36y) Controls: NR (42y)	822 (31%); NR	Hospitalised LDH	Hospital	High	II
Lee 2015 [85] Risk	South Korea	Case–control	Healthcare	Cases: Hospital patients with LDH treated conservatively, with selective nerve root block or surgery, from 2010–2011 Controls: Patients without LDH from an outpatient clinic matched for age, race and BMI Follow-up: NA	20-30y (23y)	565 (NA); NR	Clinical, hospital or surgical LDH	Clinical, hospital or surgical	High	II
Yang 2020 [144] China	China	Case–control	Healthcare	Cases: 384 Han Chinese patients with LDH Controls: 384 Han Chinese controls without back pain Follow-up: NA	NR (50y)	768 (41%)	LDH	Clinical	High	II
Zhou 2021 [117] Risk	China	Case–control	General	Cases: 6,827 patients with sciatica Controls: 134,889 controls Follow-up: NA	NR (NR)	176,899 (NR)	Sciatica	Hospital	High	II
^a^ Sun 2013 [125] ^b^ Sun 2011 [126] Risk	China	Case–control	Healthcare	Cases: Orthopaedic and spine surgery hospital patients with LDH, 2006–2009 Controls: Healthy hospital patients with medical check-up Follow-up: NA	NR (NR)	^a^ 1,008 (40%); NR ^b^ 799 (40%); NR	Clinical LDH	Clinical	High	^a^ II ^b^ I
^a^ Jacobsen 2013 [56] ^b^ Jacobsen 2012 [57] Risk	Norway	Case–control	Healthcare	Cases: Patients with LDH from two university hospitals from 2007–2009 Controls: Participants without a history of back disease from a general health survey (Nord-Trøndelag Health Study), matched for age, sex and smoking status Follow-up: NA	18-60y (41y)	510 (47%); 89%	Clinical LDH	Clinical	High	^a^ II ^b^ I
Netterstrøm 1989 [101] Incidence and risk	Denmark	Cohort	Occupational	All full-time male bus drivers employed by 3 urban bus companies linked to the Danish National Patient Register Follow-up: 7y	20-69y (NR)	2,465 (0%); 100%	Hospitalised LDH	Hospital	High	I
An 1994 [2] Risk	USA	Case–control	Healthcare	Cases: 163 consecutive surgical patients with LDH, 1987–1988 Controls: 205 inpatient controls matched on sex and age Follow-up: NA	16-78y (45y)	368 (39%); NR	LDH surgery	Surgical	High	I
Lee 2006 [84] Risk	South Korea	Case–control	Healthcare	Cases: 119 LDH levels in 111 adult patients who underwent LDH surgery, 2000–2002 Controls: 82 normal disc levels adjacent to the herniated levels in the same patients Follow-up: NA	40-49y (NR)	201 adult disc levels (37%); NR	LDH surgery	Surgical	High	I
Saftic 2006 [114] Risk	Croatia	Case–control	Healthcare	Cases: 67 adults from 9 villages on the Croatian islands with a history of LDH surgery Controls: 268 adults matched on age, sex, and village of residence/immigrant status Follow-up: NA	≥ 18y (NR)	365 (NR); NR	LDH surgery	Surgical	High	I
Kunakornsawat 2007 [82] Risk	Thailand	Case–control	Healthcare	Cases: 34 LDH levels in 34 adult patients who underwent LDH surgery, 2001–2003 Controls: 34 normal disc levels adjacent to the herniated levels in the same patients Follow-up: NA	23-45y (34y)	68 disc levels (35%); NR	LDH surgery	Surgical	High	I
Paz Aparicio 2011 [107] Risk	Spain	Case–control	Healthcare	Cases: 50 patients with clinical LDH, 2007–2008 Controls: 129 orthopaedic patients admitted for primary hip or knee arthroplasty Follow-up: NA	Cases: 23-77y (44y) Controls: 25-85y (69y)	179 (60%); NR	Clinical LDH	Clinical	High	I
Song 2013 [122] Risk	China, Finland, Japan	Case–control	General	Cases: 4,043 patients with lumbar degenerative disease Controls: 28,599 controls Follow-up: NA	NR (NR)	32,642 (NR)	LDH and Sciatica	Clinical	High	I
Cong 2014 [17] Risk	China	Case–control	Healthcare	Cases: Hospital patients with surgically managed LDH Controls: Healthy blood donors without LDH Follow-up: NA	14-49y (37y)	259 (0%); NR	Surgical LDH	Surgical	High	I
Fei 2017 [24] Risk	China	Case–control	Healthcare	Cases: 100 patients with LDH, from 2012–2015 Controls: 100 age and sex matched asymptomatic controls Follow-up: NA	18-35y (27y)	200 (34%)	Clinical LDH	Clinical	High	I
Keser 2017 [77] Risk	Turkey	Case–control	Healthcare	Cases: 50 LDH patients, from 2015–2016 Controls: 50 age and sex matched controls with no LDH on imaging Follow-up: NA	NR (38y)	100 (50%)	Hospitalised LDH	Hospital	High	I
Yaltirik 2019 [142] Risk	Turkey	Case–control	Healthcare	Cases: 108 patients with LDH followed by the Neurosurgery Department Controls: 103 healthy individuals who never experienced LBP Follow-up: NA	NR (40y)	211 (52%)	Clinical LDH	Clinical	High	I
Wang 2020 [136] Risk	China	Case–control	Healthcare	Cases: 100 patients with single-level LDH surgery, from 2015–2019 Controls: 100 sex and age matched with CT of lumbar spine Follow-up: NA	NR (49y)	200 (45%)	LDH surgery	Surgical	High	I
Bailey 2022 [3] Incidence and risk	USA	Cohort	Occupational	NASA astronauts spending time in space, from 2011–2018	NR (51y)	12 (17%)	Clinical LDH	Clinical	High	I

BMI, body mass index; LDH, lumbar disc herniation; Mod, Moderate; N, study size; NA, not applicable; NR, not reported; ROB, risk of bias; y, years

#### What is the incidence of lumbar disc herniation (LDH) with radiculopathy in adults?

We accepted 31 cohort studies on the incidence of LDH with radiculopathy in adults (summarised in Tables 1 and 2, and fully detailed in Appendix 4 [Online Supplementary Material]).

The incidence of LDH with radiculopathy varied by source population and case definition. Among nine general population studies using a surgical or hospital-based case definition [23–27, 30, 33, 47, 51, 57, 58], the annual incidence ranged from 0.2 to 2.5 per 1,000 persons, whereas one study with a clinical case definition reported an annual incidence of 13.4 per 1,000 persons [49]. In seven studies investigating occupational populations using a case definition based on surgery or hospitalisation, the annual incidence of LDH with radiculopathy ranged between 0.04 and 2.5 per 1,000 persons [35, 38, 39, 43, 45, 48, 52, 54]. One study from Finland found a higher incidence density of 7.8 per 1,000 person-years of hospitalised LDH in male military conscripts performing compulsory service between 1990 and 2002 [32].

Studies using clinical signs and symptoms as the basis for case definition of LDH with radiculopathy found higher estimates of incidence. The definition of clinical signs and symptoms varied among studies, most studies defined it as low back pain and pain with a radicular pattern with or without accompanying neurological deficits such as motor or sensory deficits. Three studies among females from the US-based Nurses Health’ Study [29], military helicopter pilots in the United States [56], and Korean public officers [53], reported an annual incidence ranging from 4.1 to 26.6 per 1,000 person-years. Eight other studies described the incidence of clinical LDH with radiculopathy in electrical and gas workers [40], Veterans Affairs outpatients [41, 42], house painters and concrete workers [34], forest industry workers [28], office workers, machine operators, and carpenters [36], Taiwanese healthcare professionals [50, 55], and professional drivers from various industries [46]. These reported annual incidence estimates ranging from a low of 2.8 per 1,000 persons among healthcare professionals in Taiwan [50], to a high of 218 per 1,000 persons among male earth-moving machine drivers in Italy [46].

#### What are the risk factors for LDH with radiculopathy in adults?

**Fig. 2:**
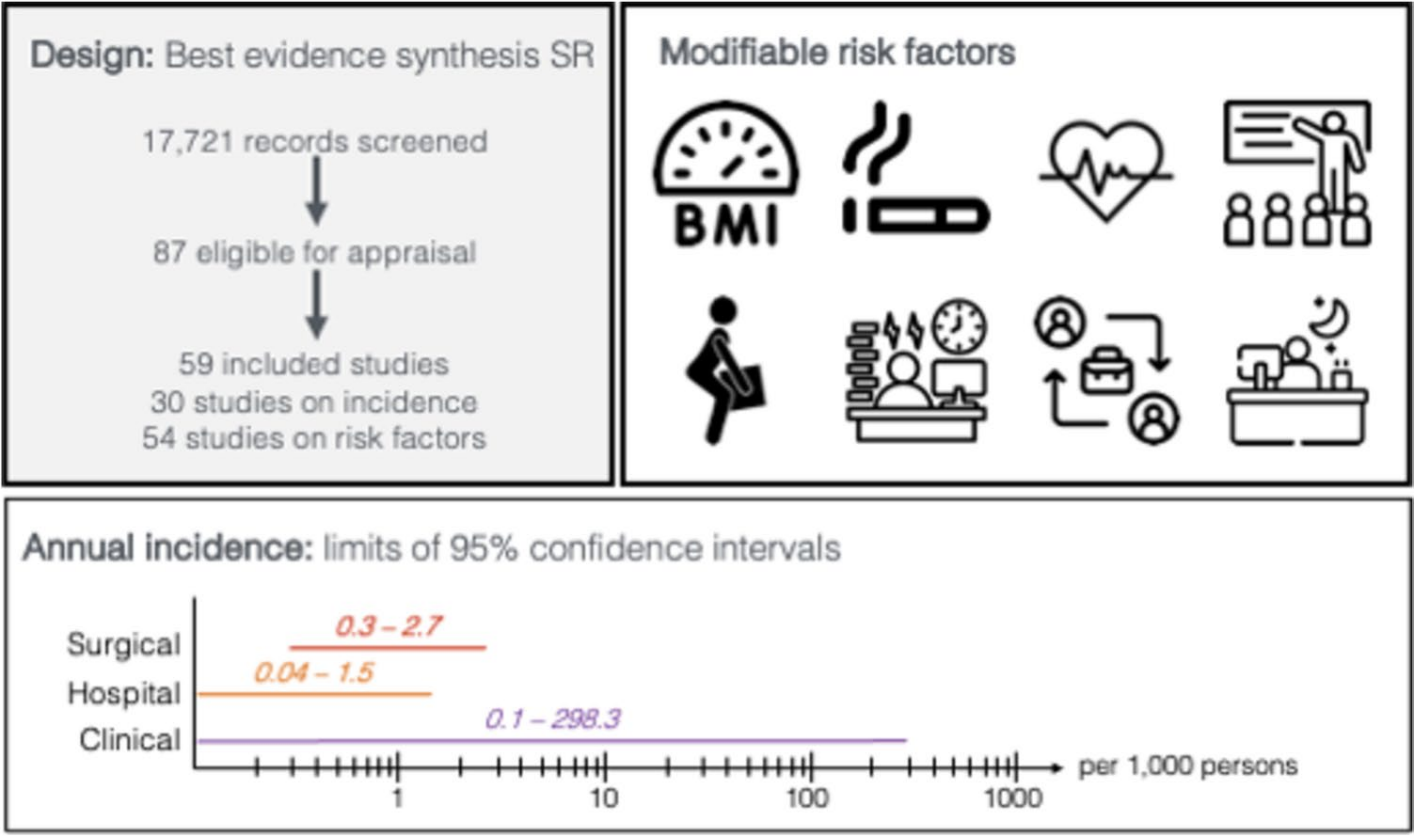
Infographic summarising the main findings of the systematic review showing limits of annual incidence 95% CIs and modifiable risk factors for LDH with radiculopathy in adults [28–30, 32, 87, 105, 130, 131]. The lower and upper bound limits of the 95% CIs ranged from 0.3 to 2.7 per 1,000 persons for surgical case definitions, from 0.04 to 1.5 per 1,000 persons for hospital-based case definitions, and from 0.1 to 298.3 per 1,000 persons for clinical case definitions. Potential modifiable risk factors: higher BMI, smoking, cardiovascular risk factors (in women), lower education, greater cumulative occupational lumbar load by forward bending postures and manual materials handling, increased time pressure at work and lower decision latitude at work, regular or irregular three-shift work, or regular night work (in women)

Our best evidence synthesis includes 25 cohort studies and 28 case–control studies informing on risk factor evidence (summarised in Tables 1 and 2, and fully detailed in Appendix 5 [Online Supplementary Material]). We report findings according to hierarchical phases of evidence, with phase III studies presenting the strongest (confirmatory) evidence of association. Throughout this section, we highlight risk factor estimates (point and 95% CI estimates) from low risk of bias phase III studies (Fig. 2).

### Phase III studies

We found evidence that mid-age (30–50 years compared with younger adults) was associated with the development of LDH with radiculopathy with the strength of associations ranging from 1.3 (1.2–1.5) to 1.8 (1.5–2.0) [39]. Jhawar et al. reported an association of LDH with radiculopathy and smoking in women (vs. nonsmokers), with a risk ratio of 1.4 (1.3–1.5) [29]. High cholesterol, diabetes mellitus, hypertension, a higher BMI, and a positive family history of coronary heart disease were also associated with the risk of LDH with radiculopathy in women with associations ranging from 1.1 (1.0–1.3) to 1.5 (1.2–2.0) [29]. The associations between LDH with radiculopathy and greater cumulative occupational lumbar load by forward bending postures and manual materials handling ranged from 1.6 (1.1–2.7) to 3.7 (2.3–6.0) [74, 100]. Two studies found weak to moderate associations of LDH with radiculopathy and whole-body vibration, with 95% CIs indicating both narrow (OR 1.4 [1.1–1.6]) [54] and wide (OR 1.8 [0.4–9.0]) [100] ranges of plausible true risk estimates. Prolonged sitting combined with moderate-to-vigorous physical activity in leisure time was also associated with LDH, with risk estimates ranging from 1.3 (1.1–1.6) to 2.2 (1.1–4.2) [47].

### Phase II studies

The strength of associations for LDH with radiculopathy in males (vs. females) ranged from 1.3 to 2.2 (1.3–6.3) in low risk of bias studies [23–25, 27]. Mattila et al. reported an increased risk for LDH in persons participating 4–5 times in sports per week (vs. never; HR 2.7 [1.1–6.3]) [32]. Mental stress and number of psychological distress symptoms were associated with LDH with radiculopathy (compared with none), with associations ranging from 1.6 to 3.0 (0.9–5.9) [23–25, 28].

## Discussion

Lumbar disc herniation (LDH) with radiculopathy is associated with greater pain, disability, healthcare use, and costs compared with nonspecific low back pain. To improve healthcare and prevention efforts a better understanding of the incidence and causal risk factors of this condition are needed. Our objective was to synthesise the best available evidence on the incidence of and risk factors for LDH with radiculopathy in adults.

### Limitations

This literature has many limitations making it difficult to draw consistent conclusions. Of the 87 eligible studies, only 12 (14%) were deemed to be low risk of bias. It is challenging to compare incidence rates and risk factors due to the amount of heterogeneity in the body of evidence. We found no universally accepted definition of LDH with radiculopathy. We tried to apply a prespecified, consistent definition of LDH with radiculopathy across different settings with varying levels of diagnostic investigation and differing susceptibilities to detection or ascertainment bias. The lack of a standard definition for the lower threshold of severity of LDH with radiculopathy leads to potential misclassification of cases. Many studies included hospital admissions only. This is problematic since hospital admission policies for LDH vary over time and jurisdictions [23]. Even studies that capture emergency department cases miss the majority that are treated at outpatient clinics or receive no treatment at all, underestimating the true incidence of LDH with radiculopathy.

Our systematic review has two main limitations. First, the strength of our findings is limited by the considerable variation in the methods and quality of the primary literature including variation in the source populations, case definitions, and assessment and control of confounding. We minimised the effect of these potential sources of bias by rating the overall risk of bias as low, moderate, and high; and by classifying studies as phase I, II and III and giving greater weight to confirmatory phase III studies and studies rated as low risk of bias. Second, when assessing evidence on certain risk factors, we observed varying results that may be due to population-specific effects rather than contradictory findings, as well as mostly weak associations, which also limits our ability to draw conclusions.

### What is the incidence of lumbar disc herniation (LDH) with radiculopathy in adults?

Annual incidence estimates for hospitalised or surgically managed LDH, ranged between 0.2 to 1.3 per 1,000 persons among the general population. However, most LDH with radiculopathy is not treated in hospitals. The annual incidence of LDH with radiculopathy defined based on clinical signs and symptoms varied from 2.8 per 1,000 persons (0.3%) among healthcare professionals in Taiwan [50], to 218 per 1,000 persons (22%) among male earthmoving machine drivers in Italy [46].

### What are the risk factors for LDH with radiculopathy in adults?

We identified a range of risk factors whose individual contribution is unknown. While there is evidence that occupational factors such as high cumulative lumbar load from forward bending may be a strong risk factor for LDH with radiculopathy, likely combinations of risk factors including individual and behavioural factors interact to cause LDH with radiculopathy, and the specific combination of risk factors varies between persons.

We found evidence from low risk of bias phase III studies that mid-age (30–50 years), higher BMI and cardiovascular risk factors (in women), smoking, and greater cumulative occupational lumbar load by forward bending postures and manual materials handling are associated with the development of LDH with radiculopathy. Furthermore, evidence from low risk of bias phase II studies suggests that male sex, lower education, higher perceived risk of work injury, lower decision latitude at work, regular or irregular three-shift work or regular night work in women, and increased time pressure at work are associated with the development of LDH with radiculopathy.

Despite the limitations of this literature, there are some important conclusions that we can make. Our findings suggest that the incidence is higher among occupational populations and is highly dependent on the source population, case definition, and ascertainment method. Little is known about the incidence of clinically defined LDH with radiculopathy in the general population—only one admissible study [49] used a clinical case definition of LDH with radiculopathy to investigate incidence and risk factors among a general population. Overall, the limited information on risk factors suggests individual, behavioural, and occupational domains as potential sources of causal components for LDH with radiculopathy in adults.

### Recommendations

Our review highlights existing gaps in knowledge of the etiology of LDH with radiculopathy. Better quality (low risk of bias) descriptive and analytic investigations are needed as the incidence and risk factors of this disabling and costly condition have not been adequately described or examined in the literature, especially in the general population [6, 8]. Furthermore, incidence density rates are preferable to cumulative incidence estimates since they provide a more accurate measure of the population-time at risk and a more accurate estimate of the rate at which an outcome occurs.

Future research should clearly and explicitly describe their source and target populations and issues related to their sampling frames. Accurate and complete ascertainment of both the cases developing the outcome and the at-risk population (i.e., the population at risk of experiencing the outcome) are required to produce valid incidence estimates. To reduce the amount of information bias surrounding the diagnosis and case definitions of LDH with radiculopathy, there is a need for workable clinical and surveillance definitions of LDH with radiculopathy and subsequent validation studies. For example, in their 1997 study evaluating the accuracy of diagnostic codes for identifying spinal disorders in health administrative databases, Faciszewski et al. reported that hospital discharge codes for LDH had a high positive predictive value (93%) compared with physician chart review [135]. More recently, Genevay and colleagues developed and validated clinical classification criteria to identify patients with radicular pain caused by LDH (RAPIDH score ≥ 11; specificity 90%, sensitivity 71%, area under curve 0.91) [136]. These diagnostic studies aiming to improve the evidence base for the reliable and valid diagnosis and classification of LDH with radiculopathy are promising and more research along this line of inquiry is warranted and needed [137].

Risk factors should be studied in well-designed cohort or case–control study designs using multivariable statistical analysis allowing the identification of causal risk factors after adjustment for important confounders. Future etiological research on LDH with radiculopathy ought to be more explicitly conceptualised and designed to estimate causal associations between modifiable exposures of interest and LDH. Regrettably, Gleadhill and colleagues found that more than 6 in 10 observational studies in spinal pain are misconceived, misaligned, or report mixed messages, with researchers using methods that signal causal intent but shrouding their intent in noncausal language [138]. There is an entire body of literature advancing causal modelling and inference [139–141] informed by causal directed acyclic graphs [142] and causal mediation analysis [143]. In sum, better understanding of the incidence and determinants of LDH with radiculopathy demands more careful attention to issues of causality, bias, methodology, and reporting [144].

## Conclusions

The annual incidence of LDH with radiculopathy varies widely, reflecting the variability in evidence due to differences in case definitions and study populations. From phase III and low risk of bias studies, key risk factors have been identified. These include occupational physical factors, particularly cumulative lumbar load from activities like forward bending and manual materials handling, which show a strong association with LDH. Lifestyle factors such as smoking and high BMI are also contributors. Additionally, mental stress has been highlighted as a potential risk factor.

This evidence underscores the need for future research to focus on establishing clear, standardised case definitions and employing robust methodologies to elucidate causal relationships. Understanding these relationships is vital for developing effective prevention strategies and guiding clinical practices to mitigate the incidence and impact of LDH with radiculopathy. The findings from high-quality studies will provide a more reliable basis for formulating targeted interventions, particularly in occupational settings and through lifestyle modifications.

## Supplementary Information

Below is the link to the electronic supplementary material.

Supplementary file1 (DOCX 520 KB)

Supplementary file2 (DOCX 294 KB)

Supplementary file3 (DOCX 177 KB)
