# Supplementary material for: Incidence of and risk factors for lumbar disc herniation with radiculopathy in adults: a systematic review
Source: Eur Spine J. 2024 Oct 25;34(1):263–94. doi: 10.1007/s00586-024-08528-8 (PMC11754359; doi:10.1007/s00586-024-08528-8)
Supplement: Supplementary file 1 — Supplementary file1 (DOCX 520 KB) [file 586_2024_8528_MOESM1_ESM.docx]

Online Supplementary Material

**Incidence of and risk factors for lumbar disc herniation with radiculopathy in adults: a systematic review**

**Authors:** Cesar A. Hincapié DC PhD, Daniela Kroismayr MChiroMed MMed, Léonie Hofstetter DCM, Astrid Kurmann MChiroMed, Carol Cancelliere DC PhD, Y. Raja Rampersaud MD MSc, Eleanor Boyle PhD, George A. Tomlinson PhD, Alejandro R. Jadad MD DPhil, Jan Hartvigsen DC PhD, Pierre Côté DC PhD, J. David Cassidy PhD DrMedSc. Submitted to Clinical Orthopeadics and Related Research, 2024.

Table of contents

[Appendix 1. Detailed search strategies 2](#_Toc166869722)

[Appendix 2. Reasons for exclusion of excluded reports 7](#_Toc166869723)

[Appendix 3. Summary risk of bias ratings for all eligible studies (n = 86) 14](#_Toc166869724)

[Appendix 4. Admissible studies examining the incidence of LDH with radiculopathy in adults (n = 30) 21](#_Toc166869725)

[Appendix 5. Admissible studies examining risk factors for LDH with radiculopathy in adults (n = 53) 33](#_Toc166869726)

[Appendix 6. Inadmissible studies examining the incidence of LDH with radiculopathy (n = 7) 67](#_Toc166869727)

[Appendix 7. Inadmissible studies examining risk factors for LDH with radiculopathy (n = 27) 69](#_Toc166869728)

[References 80](#_Toc166869729)

**Appendix 1.** Detailed search strategies

**Medline search strategy**

| 1 | Intervertebral Disk Displacement/ |
| --- | --- |
| 2 | Intervertebral Disk Displacement?.mp. |
| 3 | Intervertebral Disc Displacement?.mp. |
| 4 | Inter-vertebral Disk Displacement?.mp. |
| 5 | Inter-vertebral Disc Displacement?.mp. |
| 6 | Sciatica/ |
| 7 | sciatica.mp. |
| 8 | ((hernia* or ruptur* or prolaps* or protru* or bulg* or slip* or displac*) adj3 (disc? or disk? or nuclei or nucleus)).mp. |
| 9 | ((extru* or sequestra* or migrat*) adj2 (disc? or disk? or nuclei or nucleus)).mp. |
| 10 | (discal adj1 hernia*).mp. |
| 11 | (discus adj1 hernia*).mp. |
| 12 | ischialgi*.mp. |
| 13 | lumboischialgi*.mp. |
| 14 | lumbo-ischialgi*.mp. |
| 15 | cauda equina syndrome?.mp. |
| 16 | or/1-15 |
| 17 | Lumbar Vertebrae/ |
| 18 | Intervertebral Disk/ |
| 19 | Lumbosacral Region/ |
| 20 | ((lumbar or lumbosacral or lumbo-sacral) adj1 (spine or region or area or vertebr* or disk? or disc?)).mp. |
| 21 | discus intervertebra*.mp. |
| 22 | (spinal adj1 (disk? or disc?)).mp. |
| 23 | (low back adj1 (spine or region or area or vertebr*)).mp. |
| 24 | Intervertebral Disk?.mp. |
| 25 | Intervertebral Disc?.mp. |
| 26 | Inter-vertebral Disk?.mp. |
| 27 | Inter-vertebral Disc?.mp. |
| 28 | or/17-27 |
| 29 | 16 and 28 |
| 30 | incidence/ |
| 31 | exp risk/ |
| 32 | exp Cohort Studies/ |
| 33 | exp Case-Control Studies/ |
| 34 | epidemiologic studies/ |
| 35 | incidence.mp. |
| 36 | risk factor?.mp. |
| 37 | cohort?.mp. |
| 38 | case control?.mp. |
| 39 | risk*.mp. |
| 40 | between group*.tw. |
| 41 | relative risk*.tw. |
| 42 | etiolog*.mp. |
| 43 | exp Epidemiology/ |
| 44 | Epidemiologic Methods/ |
| 45 | epidemiolog*.mp. |
| 46 | ep.fs. |
| 47 | or/30-46 |
| 48 | 29 and 47 |
| 49 | limit 48 to yr="1970-Current" |
| 50 | limit 49 to human |
| 51 | remove duplicates from 50 |
| 52 | animals/ not (animals/ and humans/) |
| 53 | 51 not 52 |
| 54 | 51 or 53 |
| 55 | limit 54 to (english or french or spanish) |

**Embase search strategy**

| #1. | 'intervertebral disk hernia'/exp OR ‘intervertebral disk hernia' |
| --- | --- |
| #2. | 'intervertebral disk displacement' OR 'intervertebral disc displacement' OR 'inter-vertebral disk displacement' OR ‘inter-vertebral disk displacement’ |
| #3. | 'sciatica' |
| #4. | (hernia* OR ruptur* OR prolaps* OR protru* OR bulg* OR slip* OR displac*) NEAR/1 (disc OR disk OR nuclei OR nucleus) |
| #5. | (extru* OR sequestra* OR migrat*) NEAR/1 (disc OR disk OR nuclei OR nucleus) |
| #6. | discal NEAR/1 hernia* |
| #7. | discus NEAR/1 hernia* |
| #8. | ischialgi* |
| #9. | lumboischialgi* |
| #10. | 'lumbo ischialgi*' |
| #11. | 'cauda equina syndrome' |
| #12. | #1 OR #2 OR #3 OR #4 OR #5 OR #6 OR #7 OR #8 OR #9 OR #10 OR #11 |
| #13. | 'lumbar vertebrae' |
| #14. | 'lumbar vertebra' |
| #15. | 'intervertebral disk' |
| #16. | 'lumbosacral region' |
| #17. | (lumbar OR lumbosacral OR 'lumbo sacral') NEAR/1 (spine OR region OR area OR vertebr* OR disk OR disc) |
| #18. | discus NEAR/1 intervertebra* |
| #19. | spinal NEAR/1 (disc OR disk) |
| #20. | 'low back' NEAR/1 (spine OR region OR area OR vertebr*) |
| #21. | 'intervertebral disc' OR 'intervertebral disk' OR 'inter-vertebral disk' OR 'inter-vertebral disc' |
| #22. | #13 OR #14 OR #15 OR #16 OR #17 OR #18 OR #19 OR #20 OR #21 |
| #23. | #12 AND #22 |
| #24. | incidence |
| #25. | risk |
| #26. | 'case control study' |
| #27. | 'case control studies' |
| #28. | 'cohort study' |
| #29. | 'epidemiologic studies' |
| #30. | 'risk factor' |
| #31. | cohort |
| #32. | 'case control' |
| #33. | risk* |
| #34. | 'between group' |
| #35. | ‘relative risk*' |
| #36. | etiolog* |
| #37. | 'epidemiology' |
| #38. | 'epidemiologic methods' |
| #39. | #24 OR #25 OR #26 OR #27 OR #28 OR #29 OR #30 OR #31 OR #32 OR #33 OR #34 OR #35 OR #36 OR #37 OR #38 |
| #40. | #23 AND #39 |
| #41. | #40 AND [humans]/lim |
| #42. | #41 AND ([english]/lim OR [french]/lim OR spanish]/lim) |
| #43. | #42 AND [1970-2023]/py |

**Cochrane Database of Systematic Reviews (CDSR) search strategy**

| #1 | MeSH descriptor: [Intervertebral Disc Displacement] explode all trees |
| --- | --- |
| #2 | Intervertebral disk displacement or Intervertebral disc displacement or Inter-vertebral disk displacement or Inter-vertebral disk displacement |
| #3 | sciatica |
| #4 | MeSH descriptor: [Sciatica] explode all trees |
| #5 | (hernia* or ruptur* or prolaps* or protru* or bulg* or slip* or displac*) and (disc or disk or nuclei or nucleus) |
| #6 | (extru* or sequestra* or migrat*) and (disc or disk or nuclei or nucleus) |
| #7 | discal hernia* |
| #8 | discus hernia* |
| #9 | ischialgi* |
| #10 | lumboischialgi* |
| #11 | lumbo-ischialgi* |
| #12 | cauda equina syndrome |
| #13 | {OR #1-#12} |
| #14 | lumbar vertebrae |
| #15 | intervertrebral Disc |
| #16 | lumbosacral region |
| #17 | (lumbar or lumbosacral or lumbo-sacral) and (spine or region or area or vertebr* or disk or disc) |
| #18 | discus intervertebra* |
| #19 | spinal and (disc or disk) |
| #20 | low back and (spine or region or area or vertebr*) |
| #21 | intervertebral disk |
| #22 | intervertebral disc |
| #23 | Inter-vertebral disk |
| #24 | inter-vertebral disc |
| #25 | {OR #14-#24} |
| #26 | {AND #13, #25} |
| #27 | incidence |
| #28 | risk |
| #29 | cohort studies |
| #30 | case-control studies |
| #31 | epidemiologic studies |
| #32 | risk factor |
| #33 | cohort |
| #34 | case control |
| #35 | risk* |
| #36 | between group* |
| #37 | relative risk* |
| #38 | etiolog* |
| #39 | Epidemiology |
| #40 | Epidemiologic Methods |
| #41 | epidemiolog* |
| #42 | {OR #27-#41} |
| #43 | {AND #26, #42} with Cochrane Library publication date between inception and Sep 2023, in Cochrane Reviews |

**Cochrane Central Register of Controlled Trials (CENTRAL) search strategy**

| #1 | MeSH descriptor: [Intervertebral Disc Displacement] explode all trees |
| --- | --- |
| #2 | "Intervertebral disk displacement" or "Intervertebral disc displacement" or "Inter-vertebral disk displacement" or "Inter-vertebral disk displacement" |
| #3 | sciatica |
| #4 | MeSH descriptor: [Sciatica] explode all trees |
| #5 | (hernia* or ruptur* or prolaps* or protru* or bulg* or slip* or displac*) NEXT (disc or disk or nuclei or nucleus) |
| #6 | (extru* or sequestra* or migrat*) NEXT (disc or disk or nuclei or nucleus) |
| #7 | discal NEXT hernia* |
| #8 | discus NEXT hernia* |
| #9 | ischialgi* |
| #10 | lumboischialgi* |
| #11 | lumbo-ischialgi* |
| #12 | "cauda equina syndrome" |
| #13 | {OR #1-#12} |
| #14 | "lumbar vertebrae" |
| #15 | "Intervertrebral Disc" |
| #16 | "lumbosacral region" |
| #17 | (lumbar or lumbosacral or lumbo-sacral) NEXT (spine or region or area or vertebr* or disk or disc) |
| #18 | discus NEXT intervertebra* |
| #19 | spinal NEXT (disc or disk) |
| #20 | low back NEXT (spine or region or area or vertebr*) |
| #21 | "intervertebral disk" |
| #22 | "intervertebral disc" |
| #23 | "Inter-vertebral disk" |
| #24 | "inter-vertebral disc" |
| #25 | {OR #14-#24} |
| #26 | {AND #13, #25} |
| #27 | incidence |
| #28 | risk |
| #29 | cohort studies |
| #30 | "case-control studies" |
| #31 | "epidemiologic studies" |
| #32 | "risk factor" |
| #33 | cohort |
| #34 | "case control" |
| #35 | risk* |
| #36 | "between group*" |
| #37 | "relative risk*" |
| #38 | etiolog* |
| #39 | Epidemiology |
| #40 | "Epidemiologic Methods" |
| #41 | epidemiolog* |
| #42 | {OR #27-#41} |
| #43 | {AND #26, #42} with Cochrane Library publication date between Jan 1970 and Sep 2023, in Trials |

**Database of Abstracts of Reviews of Effects (DARE) search strategy**

| 1 | [Intervertebral Disc Displacement/ [NB: disk changed to DISK to match heading]] |
| --- | --- |
| 2 | Intervertebral Disk Displacement?.mp. |
| 3 | Intervertebral Disc Displacement?.mp. |
| 4 | Inter-vertebral Disk Displacement?.mp. |
| 5 | Inter-vertebral Disc Displacement?.mp. |
| 6 | [Sciatica/] |
| 7 | sciatica.mp. |
| 8 | ((hernia* or ruptur* or prolaps* or protru* or bulg* or slip* or displac*) adj3 (disc? or disk? or nuclei or nucleus)).mp. |
| 9 | ((extru* or sequestra* or migrat*) adj2 (disc? or disk? or nuclei or nucleus)).mp. |
| 10 | (discal adj1 hernia*).mp. |
| 11 | (discus adj1 hernia*).mp. |
| 12 | ischialgi*.mp. |
| 13 | lumboischialgi*.mp. |
| 14 | lumbo-ischialgi*.mp. |
| 15 | cauda equina syndrome?.mp. |
| 16 | or/1-15 |
| 17 | [Lumbar Vertebrae/] |
| 18 | [Intervertebral Disc/ [NB: disk changed to DISK to match heading]] |
| 19 | [Lumbosacral Region/] |
| 20 | ((lumbar or lumbosacral or lumbo-sacral) adj1 (spine or region or area or vertebr* or disk? or disc?)).mp. |
| 21 | discus intervertebra*.mp. |
| 22 | (spinal adj1 (disk? or disc?)).mp. |
| 23 | (low back adj1 (spine or region or area or vertebr*)).mp. |
| 24 | Intervertebral Disk?.mp. |
| 25 | Intervertebral Disc?.mp. |
| 26 | Inter-vertebral Disk?.mp. |
| 27 | Inter-vertebral Disc?.mp. |
| 28 | or/17-27 |
| 29 | 16 and 28 |
| 30 | [incidence/] |
| 31 | [exp risk/] |
| 32 | [exp Cohort Studies/] |
| 33 | [exp Case-Control Studies/] |
| 34 | [epidemiologic studies/] |
| 35 | incidence.mp. |
| 36 | risk factor?.mp. |
| 37 | cohort?.mp. |
| 38 | case control?.mp. |
| 39 | risk*.mp. |
| 40 | between group*.tw. |
| 41 | relative risk*.tw. |
| 42 | etiolog*.mp. |
| 43 | [exp Epidemiology/] |
| 44 | [Epidemiologic Methods/] |
| 45 | epidemiolog*.mp. |
| 46 | [ep.fs.] |
| 47 | or/30-46 |
| 48 | 29 and 47 with publication date between inception and 2015, in EBM Reviews |

**Appendix 2.** Reasons for exclusion of excluded reports

Excluded articles after full-text screen and the primary reason for exclusion (sorted by reason for exclusion, chronologically, and author [alphabetically]).

| Exclusion reason: Ineligible outcome of interest (N=41) |
| --- |
| 1. Zawilla NH, Darweesh H, Mansour N, et al. Matrix metalloproteinase-3, vitamin D receptor gene polymorphisms, and occupational risk factors in lumbar disc degeneration. J Occup Rehabil. 2014;24(2):370-381. doi:10.1007/s10926-013-9472-7 2. Duran S, Cavusoglu M, Hatipoglu HG, Sozmen Cılız D, Sakman B. Association between measures of Vertebral Endplate Morphology and Lumbar Intervertebral Disc Degeneration. *Canadian Association of Radiologists Journal*. 2017;68(2):210-216. doi:10.1016/j.carj.2016.11.002 3. Vinas-Rios JM, Sanchez-Aguilar M, Medina-Govea FA, Meyer F. Early recurrent lumbar disc herniation-data from the German spine registry. European Spine Journal. 2017;26(11):2978-3057. doi:10.1007/s00586-017-5336-8 4. Zhu Y, Li S, Niu F, et al. Association between IL4, IL6 gene polymorphism and lumbar disc degeneration in Chinese population. Oncotarget. 2017;8(51):89064-89071. doi:10.18632/oncotarget.21650 5. Chen Y, Ma H, Bi D, Qiu B. Association of interleukin 1 gene polymorphism with intervertebral disc degeneration risk in the Chinese Han population. Biosci Rep. 2018;38(4). doi:10.1042/BSR20171627 6. Huang WC, Kuo CH, Wu JC, Chen YC. Higher risk of intervertebral disc herniation among neurosurgeons than neurologists: 15 year-follow-up of a physician cohort. J Clin Med. 2018;7(8):198. doi:10.3390/jcm7080198 7. Kitis S, Coskun ZM, Tasdemir P, Tuncez E, Zamani AG, Acar A. Analysis of genetic polymorphisms associated with intervertebral disc degeneration. Cell Mol Biol (Noisy-le-grand). 2018;64(10):61-65. 8. Korshøj M, Jørgensen MB, Hallman DM, Lagersted-Olsen J, Holtermann A, Gupta N. Prolonged sitting at work is associated with a favorable time course of low-back pain among blue-collar workers: a prospective study in the DPhacto cohort. Scand J Work Environ Health. 2018;44(5):530-538. doi:10.5271/sjweh.3726 9. Zhou X, Cheung CL, Karasugi T, et al. Trans-ethnic polygenic analysis supports genetic overlaps of lumbar disc degeneration with height, body mass index, and bone mineral density. Front Genet. 2018;9. doi:10.3389/fgene.2018.00267 10. Schistad EI, Bjorland S, Røe C, et al. Five-year development of lumbar disc degeneration—a prospective study. Skeletal Radiol. 2019;48(6):871-879. doi:10.1007/s00256-018-3062-x 11. Akarirmak U, Sari H. Lumbar disc herniation and vitamin d receptor gene polymorphisms in Turkish patients. Osteoporosis International. 2020;31(S1):133-621. doi:10.1007/s00198-020-05696-3 12. Ding Y, Lv S, Li G, Dong S, Sun X, Chen Y. Scheuermann’s disease as a risk factor for lumbar disc herniation recurrence. J Coll Physicians Surg Pak. 2020;30(06):584-589. doi:10.29271/jcpsp.2020.06.584 13. Hung IYJ, Shih TTF, Chen BB, Liou SH, Ho IK, Guo YL. The roles of lumbar load thresholds in cumulative lifting exposure to predict disk protrusion in an Asian population. BMC Musculoskelet Disord. 2020;21(1):169. doi:10.1186/s12891-020-3167-y 14. Kiraz M, Demir E. Relationship of lumbar disc degeneration with hemoglobin value and smoking. Neurochirurgie. 2020;66(5):373-377. doi:10.1016/j.neuchi.2020.06.133 15. Öz T, Kaya İ, Nursal AF, Aydın HE, Demir O, Yiğit S. ACAN gene VNTR polymorphism and intervertebral disc degeneration in a Turkish population. Medical Bulletin of Haseki. 2020;58(4):309-314. doi:10.4274/haseki.galenos.2020.6006 16. Wirries N, Schwarze M, Daentzer D, Skutek M. Total hip arthroplasty and lumbar spine disorders: Plain co-existence or mutual influence? Orthop Rev (Pavia). 2020;12(2). doi:10.4081/or.2020.8546 17. Baker HP, Mosenthal W, Qin C, Volchenko E, Athiviraham A. Is average club head speed a risk factor for lower back injuries in professional golfers? A retrospective case control study. Phys Sportsmed. 2021;49(2):214-218. doi:10.1080/00913847.2020.1809968 18. Abdallah A, Emel E, Güler Abdallah B. Factors associated with the recurrence of lumbar disk herniation: biomechanical–radiological and demographic factors. Neurol Res. 2022;44(9):830-846. doi:10.1080/01616412.2022.2056340 19. Choi TY, Chang MY, Lee SH, Cho JG, Lee S. Psoas muscle measurement as a predictor of recurrent lumbar disc herniation: A retrospective blind study. Medicine. 2022;101(26):e29778. doi:10.1097/MD.0000000000029778 20. Guo J, Li G, Ji X, et al. Clinical and radiological risk factors of early recurrent lumbar disc herniation at six months or less: a clinical retrospective analysis in one medical center. Pain Physician. 2022;25(7):E1039-E1045. 21. Iachina M, Ljungdalh P, Nørgård BM, Garvik O, Stenager E, Schiøttz-Christensen B. Psychiatric disorders, diagnosed in psychiatric clinics, in patients with back pain: A cohort study. Scand J Public Health. 2023;51(8):1153-1160. doi:10.1177/14034948221100105 22. Kao YC, Chen JY, Chen HH, Liao KW, Huang SS. The association between depression and chronic lower back pain from disc degeneration and herniation of the lumbar spine. The International Journal of Psychiatry in Medicine. 2022;57(2):165-177. doi:10.1177/00912174211003760 23. Kasch R, Truthmann J, Hancock MJ, et al. Association of lumbar MRI findings with current and future back pain in a population-based cohort study. Spine. 2022;47(3):201-211. doi:10.1097/BRS.0000000000004198 24. Konovalov NA, Nazarenko AG, Brinyuk ES, Kaprovoy SV, Beloborodov VA, Stepanov IA. Risk factors for recurrent lumbar disk herniation. Coluna/Columna. 2022;21(4). doi:10.1590/s1808-185120222104263325 25. Ono K, Ohmori K, Yoneyama R, Matsushige O, Majima T. Risk factors and surgical management of recurrent herniation after full-endoscopic 26. Siccoli A, Staartjes VE, Klukowska AM, Muizelaar JP, Schröder ML. Overweight and smoking promote recurrent lumbar disk herniation after discectomy. European Spine Journal. 2022;31(3):604-613. doi:10.1007/s00586-022-07116-y 27. Thakar S, Raj V, Neelakantan S, et al. Spinal morphometry as a novel predictor for recurrent lumbar disc herniation requiring revision surgery: results of a case control study. Neurol India. 2022;70(8):211. doi:10.4103/0028-3886.360932 28. Wang F, Chen K, Lin Q, et al. Earlier or heavier spinal loading is more likely to lead to recurrent lumbar disc herniation after percutaneous endoscopic lumbar discectomy. J Orthop Surg Res. 2022;17(1):356. doi:10.1186/s13018-022-03242-x 29. Wang X, Liu H, Wang W, et al. Comparison of multifidus degeneration between scoliosis and lumbar disc herniation. BMC Musculoskelet Disord. 2022;23(1):891. doi:10.1186/s12891-022-05841-5 30. Abdallah A, Güler Abdallah B. Factors associated with the recurrence of lumbar disk herniation: non-biomechanical–radiological and intraoperative factors. Neurol Res. 2023;45(1):11-27. doi:10.1080/01616412.2022.2116525 31. Borja AJ, Connolly J, Kvint S, et al. Household income is associated with return to surgery following discectomy for far lateral disc herniation. J Neurosurg Sci. 2023;67(3). doi:10.23736/S0390-5616.21.05246-2 32. Geere JH, Swamy GN, Hunter PR, et al. Incidence and risk factors for five-year recurrent disc herniation after primary single-level lumbar discectomy. Bone Joint J. 2023;105-B(3):315-322. doi:10.1302/0301-620X.105B3.BJJ-2022-1005.R2 33. He H, Ma J, Xiong C, et al. Development and validation of a nomogram to predict the risk of lumbar disk reherniation within 2 years after percutaneous endoscopic lumbar discectomy. World Neurosurg. 2023;172:e349-e356. doi:10.1016/j.wneu.2023.01.026 34. Kızılgöz V, Aydın S, Karavaş E, Kantarcı M, Kahraman Ş. Are paraspinal muscle quantity, lumbar indentation value, and subcutaneous fat thickness related to disc degeneration? An MRI-based study. Radiography. 2023;29(2):428-435. doi:10.1016/j.radi.2023.02.004 35. Lastra-Power J, Nieves-Ríos C, Baralt-Nazario F, et al. Predictors of reoperation in hispanic-americans with recurrent lumbosacral disc herniation following primary hemilaminectomy and discectomy surgery. World Neurosurg X. 2023;18:100172. doi:10.1016/j.wnsx.2023.100172 36. Li X, Pan B, Cheng L, Li G, Liu J, Yuan F. Development and validation of a prognostic model for the risk of recurrent lumbar disc herniation after percutaneous endoscopic transforaminal discectomy. Pain Physician. 2023;26(1):81-90. 37. Mäntymäki H, Ponkilainen VT, Huttunen TT, Mattila VM. Regional variations in lumbar spine surgery in Finland. Arch Orthop Trauma Surg. 2021;143(3):1451-1458. doi:10.1007/s00402-021-04313-0 38. Monticelli M, Gelmi CAE, Scerrati A, Cavallo MA, De Bonis P. Recurrent or junctional lumbar foraminal herniated disc in patients operated with trans pars microscopic approach. Neurosurg Rev. 2023;46(1):211. doi:10.1007/s10143-023-02109-x 39. Shan ZM, Ren XS, Shi H, et al. Machine learning prediction model and risk factor analysis of reoperation in recurrent lumbar disc herniation patients after percutaneous endoscopic lumbar discectomy. Global Spine J. Published online May 10, 2023:219256822311733. doi:10.1177/21925682231173353 40. Zhong D, Wang Y, Lin L, et al. Development and validation of a nomogram to predict the risk of recurrent lower extremity radiating pain within 1 week following full-endoscopic lumbar discectomy. World Neurosurg. 2023;179:e348-e358. doi:10.1016/j.wneu.2023.08.090 41. Zhu F, Jia D, Zhang Y, et al. Moderate to severe multifidus fatty atrophy is the risk factor for recurrence after microdiscectomy of lumbar disc herniation. Neurospine. 2023;20(2):637-650. doi:10.14245/ns.2346054.027 |
| Exclusion reason: Ineligible design (N=23) |
| 1. Chadha M, Sharma G, Arora SS, Kochar V. Association of facet tropism with lumbar disc herniation. European Spine Journal. 2013;22(5):1045-1052. doi:10.1007/s00586-012-2612-5 2. Pan J, Lu X, Yang G, Han Y, Tong X, Wang Y. Lumbar disc degeneration was not related to spine and hip bone mineral densities in Chinese: facet joint osteoarthritis may confound the association. Arch Osteoporos. 2017;12(1):20. doi:10.1007/s11657-017-0315-6 3. Papic M, Papic V, Kresoja M, Munteanu V, Mikov I, Cigic T. Relation between grades of intervertebral disc degeneration and occupational activities of patients with lumbar disc herniation. Vojnosanit Pregl. 2017;74(12):1121-1127. doi:10.2298/VSP151112306P 4. Shahlaee A, Rahimi-Movaghar V. A familial incidence of L1-L2 disc herniation. J Neurosurg Sci. 2016;61(2). doi:10.23736/S0390-5616.16.03262-8 5. Ravindra VM, Senglaub SS, Rattani A, et al. Degenerative lumbar spine disease: estimating global incidence and worldwide volume. Global Spine J. 2018;8(8):784-794. doi:10.1177/2192568218770769 6. Reito A, Kyrölä K, Pekkanen L, Paloneva J. Specific spinal pathologies in adult patients with an acute or subacute atraumatic low back pain in the emergency department. Int Orthop. 2018;42(12):2843-2849. doi:10.1007/s00264-018-3983-y 7. Läubli R, Brugger R, Pirvu T, et al. Disproportionate vertebral bodies and their impact on lumbar disc herniation. J Clin Med. 2021;10(14):3174. doi:10.3390/jcm10143174 8. Zehra U, Cheung JPY, Bow C, et al. Spinopelvic alignment predicts disc calcification, displacement, and Modic changes: Evidence of an evolutionary etiology for clinically‐relevant spinal phenotypes. JOR Spine. 2020;3(1). doi:10.1002/jsp2.1083 9. Walter SS, Lorbeer R, Hefferman G, et al. Correlation between thoracolumbar disc degeneration and anatomical spinopelvic parameters in supine position on MRI. PLoS One. 2021;16(6):e0252385. doi:10.1371/journal.pone.0252385 10. Cha E woo, Jung S mi, Lee I ho, et al. Approval status and characteristics of work-related musculoskeletal disorders among Korean workers in 2020. Ann Occup Environ Med. 2022;34(1). doi:10.35371/aoem.2022.34.e31 11. Dittmar-Johnson HM, Cruz-López F, González-Camacho E, et al. Prevalence and characteristics of upper lumbar disc herniations in our practice: a retrospective analysis. Coluna/Columna. 2022;21(1). doi:10.1590/s1808-185120222101259474 12. Pourabbas Tahvildari B, Masroori Z, Erfani MA, Solooki S, Vosoughi AR. The impact of spino-pelvic parameters on pathogenesis of lumbar disc herniation. Musculoskelet Surg. 2022;106(2):195-199. doi:10.1007/s12306-020-00693-5 13. Raymaekers V, Bamps S, Duyvendak W, et al. Real world data collection and cluster analysis in patients with sciatica due to lumbar disc herniation. Clin Neurol Neurosurg. 2022;217:107246. doi:10.1016/j.clineuro.2022.107246 14. Azemi ES, Kola S, Kola I, Tanka M, Bilaj F, Abazaj E. Lumbar disk herniation: a clinical epidemiological and radiological evaluation. Open Access Maced J Med Sci. 2022;10(B):1588-1594. doi:10.3889/oamjms.2022.8828 15. Tarabeih N, Shalata A, Higla O, Kalinkovich A, Livshits G. The search for systemic biomarkers for monitoring degenerative lumbar spinal disorders. Osteoarthr Cartil Open. 2022;4(4):100323. doi:10.1016/j.ocarto.2022.100323 16. Wang P, Chen C, Liu F, et al. The effects of ambient temperature on lumbar disc herniation: a retrospective study. Front Med (Lausanne). 2022;9. doi:10.3389/fmed.2022.811237 17. Yazici A, Yerlikaya T. The relationship between the degeneration and asymmetry of the lumbar multifidus and erector spinae muscles in patients with lumbar disc herniation with and without root compression. J Orthop Surg Res. 2022;17(1):541. doi:10.1186/s13018-022-03444-3 18. Zhao X, Liang H, Hua Z, et al. The morphological characteristics of paraspinal muscles in young patients with unilateral neurological symptoms of lumbar disc herniation. BMC Musculoskelet Disord. 2022;23(1):994. doi:10.1186/s12891-022-05968-5 19. Chen X, Li Y, Wang W, Cui P, Wang Y, Lu S. Correlation between inflammatory cytokine expression in paraspinal tissues and severity of disc degeneration in individuals with lumbar disc herniation. BMC Musculoskelet Disord. 2023;24(1):193. doi:10.1186/s12891-023-06295-z 20. Ke S, Sun T, Zhang W, Zhang J, Li Z. Are there correlations between facet joint parameters and lumbar disk herniation laterality in young adults? Journal of Clinical Neuroscience. 2023;109:50-56. doi:10.1016/j.jocn.2023.01.013 21. Ordaz A, Anderson B, Zlomislic V, et al. Paraspinal muscle gene expression across different aetiologies in individuals undergoing surgery for lumbar spine pathology. European Spine Journal. 2023;32(4):1123-1131. doi:10.1007/s00586-023-07543-5 22. Takahashi M, Iwamoto K, Tomita K, Ueda S, Igawa T, Miyauchi Y. Factors associated with spinal instability in low back lumbar diseases with leg pain: Analysis of sagittal translation and segmental angulation. J Back Musculoskelet Rehabil. 2023;36(2):437-444. doi:10.3233/BMR-220067 23. Tian G, Wang Y, Xia J, et al. Correlation of multifidus degeneration with sex, age and side of herniation in patients with lumbar disc herniation. BMC Musculoskelet Disord. 2023;24(1):652. doi:10.1186/s12891-023-06783-2 |
| Exclusion reason: Ineligible population (N=7) |
| 1. Rajasekaran S, Kanna RM, Reddy RR, et al. How reliable are the reported genetic associations in disc degeneration? Spine. 2016;41(21):1649-1660. doi:10.1097/BRS.0000000000001847 2. Chiu CD, Chen HJ, Saw HP, Yao NW, Yen HR, Kao CH. Asthma and early herniated intervertebral disc disease. Curr Med Res Opin. 2017;33(11):2019-2025. doi:10.1080/03007995.2017.1330260 3. Botti B, Rodriguez J, Mehallo C, Simon J, Close J, Rhoden I. Incidence of causes of adolescent back pain. Clinical Journal of Sport Medicine. 2019;29(2):154-161. doi:10.1097/JSM.0000000000000726 4. Aydin HE, Yigit S, Kaya I, Tural E, Tuncer S, Nursal AF. VEGF and eNOS variants may influence intervertebral disc degeneration. Nucleosides Nucleotides Nucleic Acids. 2022;41(10):982-993. doi:10.1080/15257770.2022.2093363 5. Sayin Gülensoy E, Gülensoy B. A 9-year retrospective cohort of patients with lumbar disc herniation: Comparison of patient characteristics and recurrence frequency by smoking status. Medicine. 2022;101(51):e32462. doi:10.1097/MD.0000000000032462 6. Ishihama Y, Tezuka F, Manabe H, et al. Facet joint morphology and tropism in adolescents. Spine . Published online September 4, 2023. doi:10.1097/BRS.0000000000004818 7. Ovcharov ME, Mladenovski MN, Mladenovski IN, Valkov I V., Vasilkova SB. Lumbar disc herniation in children and elderly patients. Folia Med (Plovdiv). 2023;65(4):631-637. doi:10.3897/folmed.65.e97233 |
| Exclusion reason: Ineligible publication type (N=8) |
| 1. Doraiswamy R, Ramaswami K, Subramanian R, Srinivasan DK, Sivasankaran B. Association of Vitamin‐D receptor polymorphisms with degenerative disc disease in Indian population. The FASEB Journal. 2017;31(S1). doi:10.1096/fasebj.31.1_supplement.902.19 2. Tarnoki AD, Tárnoki DL, Szily M, et al. Relationship between obstructive sleep apnea and lumbal disc protrusion: A twin study. Twin Research and Human Genetics. 2017;20(6):564-642. doi:10.1017/thg.2017.63 3. Kim H doo, Kim DH, An YS, Jeong KS, Ahn YS, Yoon J ha. 1314 Risk assessment for back pain and lumbar degenerative disease in korean firefighters. In: Musculoskeletal Disorders. BMJ Publishing Group Ltd; 2018:A266.1-A266. doi:10.1136/oemed-2018-ICOHabstracts.760 4. Lener S, Hartmann S, Thoḿe C, Tschugg A. The impact of obesity on young individuals suffering from lumbar disc herniation: A retrospective analysis of 97 cases. Global Spine J. 2018;8(1_suppl):174S-374S. doi:10.1177/2192568218771072 5. Vidal Rodriguez S, Sánchez Benitez De Soto J. Genetic polymorphism analysis of VDR, COL1A1, GDF5, THSB2, CHST3, rank, and opg in patients with symptomatic lumbar herniated disc and surgical indication. European Spine Journal. 2018;27(10):2665-2690. doi:10.1007/s00586-018-5769-8 6. Abid M, Ullah Khan H, Huzea Abid M, et al. association of occupational risk factors with the level of lumbar disc nucleus pulposus herniation. Pakistan Journal of Medical and Health Sciences. 2021;15(10):2863-2864. doi:10.53350/pjmhs2115102863 7. Unsal D, Subasi F, Kaya AH, Gulec Yilmaz S, Yaltirik CK, Isbir T. Association of IL 1-P (rs 1143627 T/C) gene polymorphism with lumbar disc degeneration in Turkish population: a case-control study. European Spine Journal. 2021;30(11):3328-3414. doi:10.1007/s00586-021-07017-6 8. Hur J, Hong JT. Risk factor analysis for inferior clinical outcome and recurrence after full-endoscopic interlaminar discectomy (FEID) for lumbar disc herniations (LDH); A prospective observational study. Global Spine J. 2022;12(3_suppl):205S-355S. doi:10.1177/21925682221096075 |

**Appendix 3.** Summary risk of bias ratings for all eligible studies (n = 86)

**Risk of bias – cohort studies examining incidence of LDH with radiculopathy in adults (n = 38)**

|  | 1. Question | 2. Comparable populations | 3. Group participation | 4. Outcome at enrolment | 5. Attrition | 6. Participant comparison | 7. Outcome definition | 8. Blinding | 9. Knowledge of exposure | 10. Exposure assessment | 11. Outcome assessment | 12. Repeat exposure | 13. Confounding | 14. Confidence intervals | 15. Certainty of evidence | 16. Applicability | Overall risk of bias* |
| --- | --- | --- | --- | --- | --- | --- | --- | --- | --- | --- | --- | --- | --- | --- | --- | --- | --- |
| Heliövaara, 1987† |  |  |  |  |  |  |  |  |  |  |  |  |  |  |  |  | Low |
| Heliövaara, 1987† |  |  |  |  |  |  |  |  |  |  |  |  |  |  |  |  | Low |
| Bruske-Hohlfeld, 1990 |  |  |  |  |  |  |  |  |  |  |  |  |  |  |  |  | Low |
| Zitting, 1998 |  |  |  |  |  |  |  |  |  |  |  |  |  |  |  |  | Low |
| Miranda, 2002 |  |  |  |  |  |  |  |  |  |  |  |  |  |  |  |  | Low |
| Jhawar, 2006 |  |  |  |  |  |  |  |  |  |  |  |  |  |  |  |  | Low |
| Mattila, 2008 |  |  |  |  |  |  |  |  |  |  |  |  |  |  |  |  | Low |
| Mattila, 2009 |  |  |  |  |  |  |  |  |  |  |  |  |  |  |  |  | Low |
| Hincapié, 2018 |  |  |  |  |  |  |  |  |  |  |  |  |  |  |  |  | Low |
| Wahlström, 2018 |  |  |  |  |  |  |  |  |  |  |  |  |  |  |  |  | Low |
| Balling, 2019 |  |  |  |  |  |  |  |  |  |  |  |  |  |  |  |  | Low |
| Brauer, 2020 |  |  |  |  |  |  |  |  |  |  |  |  |  |  |  |  | Low |
| Jung, 2020 |  |  |  |  |  |  |  |  |  |  |  |  |  |  |  |  | Low |
| Hurme, 1983 |  |  |  |  |  |  |  |  |  |  |  |  |  |  |  |  | Moderate |
| Heikkilä, 1989 |  |  |  |  |  |  |  |  |  |  |  |  |  |  |  |  | Moderate |
| Riihimäki, 1989 |  |  |  |  |  |  |  |  |  |  |  |  |  |  |  |  | Moderate |
| Jørgensen, 1994 |  |  |  |  |  |  |  |  |  |  |  |  |  |  |  |  | Moderate |
| Riihimäki, 1994 |  |  |  |  |  |  |  |  |  |  |  |  |  |  |  |  | Moderate |
| Leino-Arjas, 2004‡ |  |  |  |  |  |  |  |  |  |  |  |  |  |  |  |  | Moderate |
| Leino-Arjas, 2002‡ |  |  |  |  |  |  |  |  |  |  |  |  |  |  |  |  | Moderate |
| Leclerc, 2003 |  |  |  |  |  |  |  |  |  |  |  |  |  |  |  |  | Moderate |
| Jarvik, 2005§ |  |  |  |  |  |  |  |  |  |  |  |  |  |  |  |  | Moderate |
| Suri, 2014§ |  |  |  |  |  |  |  |  |  |  |  |  |  |  |  |  | Moderate |
| Sørensen, 2011# |  |  |  |  |  |  |  |  |  |  |  |  |  |  |  |  | Moderate |
| Jørgensen, 2013# |  |  |  |  |  |  |  |  |  |  |  |  |  |  |  |  | Moderate |
| Wahlström, 2012 |  |  |  |  |  |  |  |  |  |  |  |  |  |  |  |  | Moderate |
| Bovenzi, 2015 |  |  |  |  |  |  |  |  |  |  |  |  |  |  |  |  | Moderate |
| Chan, 2018 |  |  |  |  |  |  |  |  |  |  |  |  |  |  |  |  | Moderate |
| Fouquet, 2018 |  |  |  |  |  |  |  |  |  |  |  |  |  |  |  |  | Moderate |
| Han, 2018 |  |  |  |  |  |  |  |  |  |  |  |  |  |  |  |  | Moderate |
| Kim, 2018 |  |  |  |  |  |  |  |  |  |  |  |  |  |  |  |  | Moderate |
| Knox, 2018 |  |  |  |  |  |  |  |  |  |  |  |  |  |  |  |  | Moderate |
| Huang, 2019 |  |  |  |  |  |  |  |  |  |  |  |  |  |  |  |  | Moderate |
| Jäntti, 2022 |  |  |  |  |  |  |  |  |  |  |  |  |  |  |  |  | Moderate |
| Nyrhi, 2023 |  |  |  |  |  |  |  |  |  |  |  |  |  |  |  |  | Moderate |
| Bongers, 1988 |  |  |  |  |  |  |  |  |  |  |  |  |  |  |  |  | High |
| Netterstrøm, 1989 |  |  |  |  |  |  |  |  |  |  |  |  |  |  |  |  | High |
| Rivinoja, 2011 |  |  |  |  |  |  |  |  |  |  |  |  |  |  |  |  | High |
| Roquelaure, 2011 |  |  |  |  |  |  |  |  |  |  |  |  |  |  |  |  | High |
| Chung, 2013 |  |  |  |  |  |  |  |  |  |  |  |  |  |  |  |  | High |
| Makovicka, 2019 |  |  |  |  |  |  |  |  |  |  |  |  |  |  |  |  | High |
| Bailey, 2022 |  |  |  |  |  |  |  |  |  |  |  |  |  |  |  |  | High |
| SIGN checklist items for **cohort studies**:  1. The study addresses an appropriate and clearly focused question.  2. The two groups being studied are selected from source populations that are comparable in all respects other than the factor under investigation.  3. The study indicates how many of the people asked to take part did so, in each of the groups being studied.  4. The likelihood that some eligible subjects might have the outcome at the time of enrolment is assessed and taken into account in the analysis.  5. What percentage of individuals or clusters recruited into each arm of the study dropped out before the study was completed  (coded as ≤10% = green, 11% to 20% = amber, >20% = red).  6. Comparison is made between full participants and those lost to follow-up, by exposure status  7. The outcomes are clearly defined.  8. The assessment of outcome is made blind to exposure status.  9. Where blinding was not possible, there is some recognition that knowledge of exposure status could have influenced the assessment of outcome.  10. The measure of assessment of exposure is reliable.  11. Evidence from other sources is used to demonstrate that the method of outcome assessment is valid and reliable.  12. Exposure level or prognostic factor is assessed more than once.  13. The main potential confounders are identified and taken into account adequately in the design and analysis.  14. Confidence intervals are provided.  15. Taking into account clinical considerations, your evaluation of the methodology used, and the statistical power of the study, are you certain that the overall effect is due to the exposure being investigated?  16. Are the results of this study directly applicable to the objectives of this systematic review?  * How well was the study done to minimize the risk of bias or confounding, and to establish a causal relationship between exposure and effect?  Colour legend for risk of bias ratings:   \|  \| Yes / Well covered / Adequately addressed \| \| --- \| --- \| \|  \| Can’t say / Not reported / Not addressed \| \|  \| No / Poorly addressed \| \|  \| Not applicable \|   † Heliövaara 1987 and Heliövaara 1987 deemed one study represented by two publications. Ratings done at individual publication level.  ‡ Leino-Arjas 2004 and Leino-Arjas 2022 deemed one study represented by two publications.  § Jarvik 2005 and Suri 2014 deemed one study represented by two publications.  # Sørensen 2011 and Jørgensen 2013 deemed one study represented by two publications. | | | | | | | | | | | | | | | | | |

**Risk of bias – cohort studies examining risk factors for LDH with radiculopathy in adults (n = 33)**

|  | 1. Question | 2. Comparable populations | 3. Group participation | 4. Outcome at enrolment | 5. Attrition | 6. Participant comparison | 7. Outcome definition | 8. Blinding | 9. Knowledge of exposure | 10. Exposure assessment | 11. Outcome assessment | 12. Repeat exposure | 13. Confounding | 14. Confidence intervals | 15. Certainty of evidence | 16. Applicability | Overall risk of bias* |
| --- | --- | --- | --- | --- | --- | --- | --- | --- | --- | --- | --- | --- | --- | --- | --- | --- | --- |
| Jhawar, 2006 |  |  |  |  |  |  |  |  |  |  |  |  |  |  |  |  | Low |
| Hincapié, 2018 |  |  |  |  |  |  |  |  |  |  |  |  |  |  |  |  | Low |
| Wahlström, 2018 |  |  |  |  |  |  |  |  |  |  |  |  |  |  |  |  | Low |
| Balling, 2019 |  |  |  |  |  |  |  |  |  |  |  |  |  |  |  |  | Low |
| Brauer, 2020 |  |  |  |  |  |  |  |  |  |  |  |  |  |  |  |  | Low |
| Heliövaara, 1987a† |  |  |  |  |  |  |  |  |  |  |  |  |  |  |  |  | Low |
| Heliövaara, 1987b† |  |  |  |  |  |  |  |  |  |  |  |  |  |  |  |  | Low |
| Zitting, 1998 |  |  |  |  |  |  |  |  |  |  |  |  |  |  |  |  | Low |
| Miranda, 2002 |  |  |  |  |  |  |  |  |  |  |  |  |  |  |  |  | Low |
| Mattila, 2008 |  |  |  |  |  |  |  |  |  |  |  |  |  |  |  |  | Low |
| Jung, 2020 |  |  |  |  |  |  |  |  |  |  |  |  |  |  |  |  | Low |
| Huang, 2019 |  |  |  |  |  |  |  |  |  |  |  |  |  |  |  |  | Moderate |
| Leino-Arjas, 2004‡ |  |  |  |  |  |  |  |  |  |  |  |  |  |  |  |  | Moderate |
| Leino-Arjas, 2002‡ |  |  |  |  |  |  |  |  |  |  |  |  |  |  |  |  | Moderate |
| Riihimäki, 1989 |  |  |  |  |  |  |  |  |  |  |  |  |  |  |  |  | Moderate |
| Jørgensen, 1994 |  |  |  |  |  |  |  |  |  |  |  |  |  |  |  |  | Moderate |
| Riihimäki, 1994§ |  |  |  |  |  |  |  |  |  |  |  |  |  |  |  |  | Moderate |
| Pietri-Taleb, 1995§ |  |  |  |  |  |  |  |  |  |  |  |  |  |  |  |  | Moderate |
| Leclerc, 2003 |  |  |  |  |  |  |  |  |  |  |  |  |  |  |  |  | Moderate |
| Jarvik, 2005# |  |  |  |  |  |  |  |  |  |  |  |  |  |  |  |  | Moderate |
| Suri, 2014# |  |  |  |  |  |  |  |  |  |  |  |  |  |  |  |  | Moderate |
| Sørensen, 2011‖ |  |  |  |  |  |  |  |  |  |  |  |  |  |  |  |  | Moderate |
| Jørgensen, 2013‖ |  |  |  |  |  |  |  |  |  |  |  |  |  |  |  |  | Moderate |
| Wahlström, 2012 |  |  |  |  |  |  |  |  |  |  |  |  |  |  |  |  | Moderate |
| Chan, 2018 |  |  |  |  |  |  |  |  |  |  |  |  |  |  |  |  | Moderate |
| Fouquet, 2018 |  |  |  |  |  |  |  |  |  |  |  |  |  |  |  |  | Moderate |
| Han, 2018 |  |  |  |  |  |  |  |  |  |  |  |  |  |  |  |  | Moderate |
| Kim, 2018 |  |  |  |  |  |  |  |  |  |  |  |  |  |  |  |  | Moderate |
| Knox, 2018 |  |  |  |  |  |  |  |  |  |  |  |  |  |  |  |  | Moderate |
| Nyrhi, 2023 |  |  |  |  |  |  |  |  |  |  |  |  |  |  |  |  | Moderate |
| Hurme 1983 |  |  |  |  |  |  |  |  |  |  |  |  |  |  |  |  | Moderate |
| Bongers, 1988 |  |  |  |  |  |  |  |  |  |  |  |  |  |  |  |  | High |
| Chibnall, 2006 |  |  |  |  |  |  |  |  |  |  |  |  |  |  |  |  | High |
| Rivinoja, 2011 |  |  |  |  |  |  |  |  |  |  |  |  |  |  |  |  | High |
| Roquelaure, 2011 |  |  |  |  |  |  |  |  |  |  |  |  |  |  |  |  | High |
| Chung, 2013 |  |  |  |  |  |  |  |  |  |  |  |  |  |  |  |  | High |
| Netterstrøm, 1989 |  |  |  |  |  |  |  |  |  |  |  |  |  |  |  |  | High |
| Bailey, 2022 |  |  |  |  |  |  |  |  |  |  |  |  |  |  |  |  | High |
| SIGN checklist items for **cohort studies**:  1. The study addresses an appropriate and clearly focused question.  2. The two groups being studied are selected from source populations that are comparable in all respects other than the factor under investigation.  3. The study indicates how many of the people asked to take part did so, in each of the groups being studied.  4. The likelihood that some eligible subjects might have the outcome at the time of enrolment is assessed and taken into account in the analysis.  5. What percentage of individuals or clusters recruited into each arm of the study dropped out before the study was completed  (coded as ≤10% = green, 11% to 20% = amber, >20% = red).  6. Comparison is made between full participants and those lost to follow-up, by exposure status  7. The outcomes are clearly defined.  8. The assessment of outcome is made blind to exposure status.  9. Where blinding was not possible, there is some recognition that knowledge of exposure status could have influenced the assessment of outcome.  10. The measure of assessment of exposure is reliable.  11. Evidence from other sources is used to demonstrate that the method of outcome assessment is valid and reliable.  12. Exposure level or prognostic factor is assessed more than once.  13. The main potential confounders are identified and taken into account adequately in the design and analysis.  14. Confidence intervals are provided.  15. Taking into account clinical considerations, your evaluation of the methodology used, and the statistical power of the study, are you certain that the overall effect is due to the exposure being investigated?  16. Are the results of this study directly applicable to the objectives of this systematic review?  * How well was the study done to minimize the risk of bias or confounding, and to establish a causal relationship between exposure and effect?  Colour legend for risk of bias ratings:   \|  \| Yes / Well covered / Adequately addressed \| \| --- \| --- \| \|  \| Can’t say / Not reported / Not addressed \| \|  \| No / Poorly addressed \| \|  \| Not applicable \|   † Heliövaara 1987 and Heliövaara 1987 deemed one study represented by two publications. Ratings done at individual publication level.  ‡ Leino-Arjas 2004 and Leino-Arjas 2022 deemed one study represented by two publications. Ratings done at individual publication level.  § Rihiimäki 1994 and Pietri-Taleb 1995 deemed one study represented by two publications.  # Jarvik 2005 and Suri 2014 deemed one study represented by two publications.  ‖ Sørensen 2011 and Jørgensen 2013 deemed one study represented by two publications. | | | | | | | | | | | | | | | | | |

Risk of bias for case-control studies examining risk factors for LDH with radiculopathy in adults (n = 49)

|  | 1. Question | 2. Comparable populations | 3. Exclusion criteria | 4. Attrition | 5. Comparability | 6. Case definition | 7. Control ascertainment | 8. Case ascertainment | 9. Exposure measurement | 10. Confounding | 11. Confidence intervals | 12. Certainty of evidence | 13. Applicability | Overall risk of bias* |
| --- | --- | --- | --- | --- | --- | --- | --- | --- | --- | --- | --- | --- | --- | --- |
| Heliövaara, 1987a† |  |  |  |  |  |  |  |  |  |  |  |  |  | Moderate |
| Heliövaara, 1987b† |  |  |  |  |  |  |  |  |  |  |  |  |  | Moderate |
| Heliövaara, 1987c† |  |  |  |  |  |  |  |  |  |  |  |  |  | Moderate |
| Seidler, 2003 |  |  |  |  |  |  |  |  |  |  |  |  |  | Moderate |
| Seidler, 2009‡ |  |  |  |  |  |  |  |  |  |  |  |  |  | Moderate |
| Bergmann 2017‡ |  |  |  |  |  |  |  |  |  |  |  |  |  | Moderate |
| Schumann, 2010‡ |  |  |  |  |  |  |  |  |  |  |  |  |  | Moderate |
| Seidler, 2011‡ |  |  |  |  |  |  |  |  |  |  |  |  |  | Moderate |
| Kelsey, 1975a§ |  |  |  |  |  |  |  |  |  |  |  |  |  | Moderate |
| Kelsey, 1975b§ |  |  |  |  |  |  |  |  |  |  |  |  |  | Moderate |
| Kelsey, 1975c§ |  |  |  |  |  |  |  |  |  |  |  |  |  | Moderate |
| Kelsey, 1975d§ |  |  |  |  |  |  |  |  |  |  |  |  |  | Moderate |
| Kelsey, 1975e§ |  |  |  |  |  |  |  |  |  |  |  |  |  | Moderate |
| Kelsey, 1984a# |  |  |  |  |  |  |  |  |  |  |  |  |  | Moderate |
| Kelsey, 1984b# |  |  |  |  |  |  |  |  |  |  |  |  |  | Moderate |
| Mundt, 1993a‖ |  |  |  |  |  |  |  |  |  |  |  |  |  | Moderate |
| Mundt, 1993b‖ |  |  |  |  |  |  |  |  |  |  |  |  |  | Moderate |
| Zhang, 2016 |  |  |  |  |  |  |  |  |  |  |  |  |  | Moderate |
| Bjornsdottir, 2017 |  |  |  |  |  |  |  |  |  |  |  |  |  | Moderate |
| Dong, 2018Δ |  |  |  |  |  |  |  |  |  |  |  |  |  | Moderate |
| Zhu, 2018Δ |  |  |  |  |  |  |  |  |  |  |  |  |  | Moderate |
| Jing, 2018 |  |  |  |  |  |  |  |  |  |  |  |  |  | Moderate |
| Li, 2018 |  |  |  |  |  |  |  |  |  |  |  |  |  | Moderate |
| Hu, 2019◊ |  |  |  |  |  |  |  |  |  |  |  |  |  | Moderate |
| Ji, 2019◊ |  |  |  |  |  |  |  |  |  |  |  |  |  | Moderate |
| Liu, 2020◊ |  |  |  |  |  |  |  |  |  |  |  |  |  | Moderate |
| Wu, 2020◊ |  |  |  |  |  |  |  |  |  |  |  |  |  | Moderate |
| Yang, 2020◊ |  |  |  |  |  |  |  |  |  |  |  |  |  | Moderate |
| Hu, 2022◊ |  |  |  |  |  |  |  |  |  |  |  |  |  | Moderate |
| Han, 2023◊ |  |  |  |  |  |  |  |  |  |  |  |  |  | Moderate |
| Wu, 2023◊ |  |  |  |  |  |  |  |  |  |  |  |  |  | Moderate |
| Yang, 2019 |  |  |  |  |  |  |  |  |  |  |  |  |  | Moderate |
| Zhu, 2019↓ |  |  |  |  |  |  |  |  |  |  |  |  |  | Moderate |
| Tai, 2020↓ |  |  |  |  |  |  |  |  |  |  |  |  |  | Moderate |
| Luo, 2020 |  |  |  |  |  |  |  |  |  |  |  |  |  | Moderate |
| Fidan 2022 |  |  |  |  |  |  |  |  |  |  |  |  |  | Moderate |
| Noponen-Hietala, 2005 |  |  |  |  |  |  |  |  |  |  |  |  |  | Moderate |
| Mio, 2007 |  |  |  |  |  |  |  |  |  |  |  |  |  | Moderate |
| Virtanen, 2007 |  |  |  |  |  |  |  |  |  |  |  |  |  | Moderate |
| Hirose, 2008 |  |  |  |  |  |  |  |  |  |  |  |  |  | Moderate |
| Karasugi, 2009 |  |  |  |  |  |  |  |  |  |  |  |  |  | Moderate |
| Cong, 2010 |  |  |  |  |  |  |  |  |  |  |  |  |  | Moderate |
| Mu, 2014 |  |  |  |  |  |  |  |  |  |  |  |  |  | Moderate |
| Huang, 2017 |  |  |  |  |  |  |  |  |  |  |  |  |  | Moderate |
| Jiang, 2017 |  |  |  |  |  |  |  |  |  |  |  |  |  | Moderate |
| Ghandhari, 2018 |  |  |  |  |  |  |  |  |  |  |  |  |  | Moderate |
| Wang, 2018 |  |  |  |  |  |  |  |  |  |  |  |  |  | Moderate |
| Withanage, 2018 |  |  |  |  |  |  |  |  |  |  |  |  |  | Moderate |
| Zhou, 2018 |  |  |  |  |  |  |  |  |  |  |  |  |  | Moderate |
| Hrubec, 1975 |  |  |  |  |  |  |  |  |  |  |  |  |  | High |
| Zhang, 2009 |  |  |  |  |  |  |  |  |  |  |  |  |  | High |
| Zhang, 2013 |  |  |  |  |  |  |  |  |  |  |  |  |  | High |
| Chiang, 2014 |  |  |  |  |  |  |  |  |  |  |  |  |  | High |
| Lee, 2015 |  |  |  |  |  |  |  |  |  |  |  |  |  | High |
| Yang, 2020 |  |  |  |  |  |  |  |  |  |  |  |  |  | High |
| Zhou, 2021 |  |  |  |  |  |  |  |  |  |  |  |  |  | High |
| Sun, 2013¶ |  |  |  |  |  |  |  |  |  |  |  |  |  | High |
| Sun, 2011¶ |  |  |  |  |  |  |  |  |  |  |  |  |  | High |
| Jacobsen, 2013♦ |  |  |  |  |  |  |  |  |  |  |  |  |  | High |
| Jacobsen, 2012♦ |  |  |  |  |  |  |  |  |  |  |  |  |  | High |
| An, 1994 |  |  |  |  |  |  |  |  |  |  |  |  |  | High |
| Lee, 2006 |  |  |  |  |  |  |  |  |  |  |  |  |  | High |
| Saftic, 2006 |  |  |  |  |  |  |  |  |  |  |  |  |  | High |
| Kunakornsawat, 2007 |  |  |  |  |  |  |  |  |  |  |  |  |  | High |
| Paz Aparicio, 2011 |  |  |  |  |  |  |  |  |  |  |  |  |  | High |
| Song, 2013 |  |  |  |  |  |  |  |  |  |  |  |  |  | High |
| Cong, 2014 |  |  |  |  |  |  |  |  |  |  |  |  |  | High |
| Fei, 2017 |  |  |  |  |  |  |  |  |  |  |  |  |  | High |
| Keser, 2017 |  |  |  |  |  |  |  |  |  |  |  |  |  | High |
| Yaltirik, 2019 |  |  |  |  |  |  |  |  |  |  |  |  |  | High |
| Wang, 2020 |  |  |  |  |  |  |  |  |  |  |  |  |  | High |
| SIGN checklist items for **case-control studies**:  1. The study addresses an appropriate and clearly focused question  2. The cases and controls are taken from comparable populations  3. The same exclusion criteria are used for both cases and controls  4. What percentage of each group (cases and controls) participated in the study  (coded as ≥90% = green, 80% to 90% = amber, <80% = red)  5. Comparison is made between participants and non-participants to establish their similarities or differences  6. Cases are clearly defined and differentiated from controls  7. It is clearly established that controls are non-cases  8. Measures will have been taken to prevent knowledge of primary exposure influencing case ascertainment  9. Exposure status is measured in a standard, valid and reliable way  10. The main potential confounders are identified and taken into account in the design and analysis  11. Confidence intervals are provided  12. Taking into account clinical considerations, your evaluation of the methodology used and statistical power of the study, are you certain that the overall effect is due to the exposure being investigated?  13. Are the results of this review directly applicable to the objectives of this systematic review?  * How well was the study done to minimize bias?  Colour legend for risk of bias ratings:   \|  \| Yes / Well covered / Adequately addressed \| \| --- \| --- \| \|  \| Can’t say / Not reported / Not addressed \| \|  \| No / Poorly addressed \| \|  \| Not applicable \|   † Heliövaara 1987a, Heliövaara 1987b and Heliövaara 1987c deemed one study represented by three publications. Ratings done at individual publication level.  ‡ Seidler 2009, Bergmann 2017, Schumann 2010 and Seidler 2011 deemed one study represented by four publications.  § Kelsey 1975a, Kelsey 1975b, Kelsey 1975c, Kelsey 1975d and Kelsey 1975e were deemed one study represented by five publications.  # Kelsey 1984a and Kelsey 1984b deemed one study represented by two publications.  ‖ Mundt 1993a and Mundt 1993b deemed one study represented by two publications.  Δ Dong 2018 and Zhu 2018 deemed one study represented by two publications.  ◊ Hu 2019, Ji 2019, Liu 2020, Wu 2020, Yang 2020, Hu 2022, Han 2023 and Wu 2023 deemed one study represented by eight publications.  ↓ Zhu 2019 and Tai 2020 deemed one study represented by two publications.  ¶ Sun 2012 and Sun 2011 deemed one study represented by two publications.  ♦ Jacobsen 2013 and Jacobsen 2011 deemed one study represented by two publications. | | | | | | | | | | | | | | |

**Appendix 4.** Admissible studies examining the incidence of LDH with radiculopathy in adults (n = 30)

| First author, Year published  Country  Study design | Study population and setting  Follow-up  N (% female)  Age range (mean)  Participation % | Study outcome  Case definition type  Case definition | ROB | Incidence estimates (95% CI) |
| --- | --- | --- | --- | --- |
| Heliövaara, 1987 [1–3]  Finland  Cohort | Nationwide cohort of Finnish adults followed from 1970-1980 for hospitalized LDH or sciatica in the National Hospital Discharge Register  Follow-up: 11y  N=57,000 (48%)  ≥15y (NR)  83% | Hospitalized LDH or sciatica  Hospital  ICD-8 codes indicating principal diagnosis for hospitalization: 725.10 or 725.19 for LDH; 353.99 for sciatica | Low | 11-year cumulative incidence of hospitalized LDH or sciatica: 14.5 (13.5-15.5) per 1,000 persons; of hospitalized LDH: 8.0 (7.3-8.7) per 1,000 persons; of hospitalized sciatica: 6.5 (5.9-7.2) per 1,000 persons  Average annual incidence of hospitalized LDH or sciatica: 1.3 (1.2-1.4) per 1,000 persons; of hospitalized LDH: 0.7 (0.7-0.8) per 1,000 persons; of hospitalized sciatica: 0.6 (0.5-0.7) per 1,000 persons  Raw data: 825/57,000, 454/57,000, 371/57,000 |
| Bruske-Hohlfeld, 1990 [4]  USA  Cohort | All Olmsted County residents followed from 1950-1979 for any form of LDH surgery  Follow-up: 30y  N=1,028 (NR)  15-78y (42)  88% incident surgery | LDH surgery  Surgical  Any form of back surgery for suspected LDH, HNP, or fragments of disc material identified by review of complete (inpatient and outpatient) medical records  “Proven” LDH was a protruded, extruded or sequestered disc seen during surgery  “Suspected” LDH was a disc described as bulging or degenerated | Low | Incidence density of first surgery for suspected LDH: age-adjusted in men, 0.6 (0.5-0.6) per 1,000 person-years; age-adjusted in women, 0.4 (0.3-0.4) per 1,000 person-years; age- and sex-adjusted, 0.5 (0.4-0.5) per 1,000 person-years  Incidence density of first surgery for proven LDH:  age-adjusted in men, 0.5 (0.5-0.6) per 1,000 person-years; age-adjusted in women, 0.3 (0.3-0.3) per 1,000 person-years; age- and sex-adjusted, 0.4 (0.4-0.4) per 1,000 person-years  Raw data: numerators: 538, 371, 909, 485, 316, 801; denominators: NR, but census data used to derive denominator person-years |
| Zitting, 1998 [5]  Finland  Cohort | Birth cohort from 2 provinces of Finland followed from 1966-1994 for hospitalized LDH in the National Hospital Discharge Register  Follow-up: 28y  N=12,058 (NR)  15-28y among cases (NR)  92% | Hospitalized LDH  Hospital  ICD-9 diagnosis codes and corresponding ICD-8 codes used to identify all possible lumbar disc disease cases: 7221, 7227, 7244, 7225, 7242, 7245, 7561, 7384, 7385, 7213, 7214  “Confirmed” LDH was a reliable description of herniation in a surgical report or reliable evidence of a herniation on MRI, CT or myelogram with appropriate symptoms (i.e., on the same side) described in the medical records | Low | Cumulative incidence of hospitalized LDH:  in men, 9.1 (6.9-12.0) per 1,000 persons;  in women, 4.2 (2.8-6.4) per 1,000 persons  Average annual incidence: in men, 0.3 (0.3-0.4) per 1,000 persons in women, 0.2 (0.1-0.2) per 1,000 persons  Raw data: in men, 50/5,474; in women, 22/5,218 |
| Miranda, 2002 [6]  Finland  Cohort | Forest industry workers who had no sciatica during the past 12 months in 1994  Follow-up: 1y  N=2,077 (26%)  NR (45)  77% | Sciatica  Clinical  Sciatica was more than 7 days of low back pain radiating below the knee during the preceding 12 months, with a manikin used to denote the anatomic area in mailed questionnaire | Low | 1-year cumulative incidence of sciatica: 93.4 (81.6-106.7) per 1,000 persons  Raw data: 194/2,077 |
| Jhawar, 2006 [7]  USA  Cohort | Female nurses from the Nurses’ Health Study without prior LDH in 1976, were followed up in 1992  Follow-up: 16y  N=98,407 (100%)  30-55y in 1976 (NR)  ≥85% | Clinical LDH  Clinical  Self-reported physician-diagnosis of LDH that was confirmed by MRI or CT, in follow-up mailed questionnaire | Low | Incidence density: 6.2 (6.0-6.5) per 1,000 person-years  Raw data: 2,727/438,662 |
| Mattila, 2008 [8]  Finland  Cohort | Nationwide cohort of adolescents aged 14-18y, without LDH and prior hospitalized nonspecific back-related diagnosis followed up to December 31, 2001, in the National Hospital Discharge Register  Follow-up: 651,027 person-years (11y average follow-up)  N=57,408 (54%)  15-41y among cases (27 at surgery)  79% | LDH surgery  Surgical  Lumbar discectomy defined as ICD code 9211 between 1979 and 1996, and then with new codes ABC07, ABC16 and ABC26 from 1997 onwards | Low | Incidence density of LDH surgery:  0.4 (0.4-0.5) per 1,000 person-years;  in men, 0.6 (0.5-0.6) per 1,000 persons-years; in women, 0.3 (0.2-0.3) per 1,000 persons-years  Raw data: numerators: 251, 166, 85; denominators: NR |
| Mattila, 2009 [9]  Finland  Cohort | All male military conscripts without severe back diseases and performing compulsory service between 1990-2002, were followed during their service period by record linkage to the National Hospital Discharge Register  Follow-up: 267,700 person-years; 6- to 12-month military service period  N=387,070 (0%)  18-29y (20)  100% | Hospitalized LDH  Hospital  Hospitalized LDH (lumbar and other intervertebral disc disorders with radiculopathy) was ICD-10 diagnosis code M51.1 (lumbar and other intervertebral disc disorders with radiculopathy), and ICD-9 diagnosis codes 7227C and 3539X | Low | Incidence density of hospitalized LDH: 7.8 (6.7-8.3) per 1,000 person-years  Incidence densities ranged from a high of 12.3 (9.6-13.3) per 1,000 person-years in 1993, to a low of 4.2 (3.0-5.0) per 1,000 person-years 2001  Raw data: NR |
| Hincapié, 2018 [10]  Canada  Cohort with self-controlled case series analysis | All adults with acute LDH requiring ED visit and early LDH surgery from April 1994 to December 2004, using population-based Ontario healthcare databases  Follow-up: 11 years  N=195 (40%), primary analysis; N=961(NR), sensitivity analyis  ≥18y (43y)  NR | Early LDH surgery  Surgical  Patients with at least one disc surgery intervention code and a recent primary care physician or chiropractic care visit due to LDH | Low | 11-year cumulative incidence for early LDH surgery after primary care physician or chiropractic care LDH visit: 0.002 per 1,000 person-years  Average annual incidence for early LDH surgery after primary care physician or chiropractic care LDH visit: 0.0002 per 1,000 person-years  11-year cumulative incidence for early LDH surgery after primary care physician or chiropractic care LDH visit (using a more sensitive outcome case definition): 0.01 per 1,000 person-years  Average annual incidence for early LDH surgery after primary care physician or chiropractic care LDH visit (using a more sensitive outcome case definition): 0.001 per 1,000 person-years  Raw data: numerators 195, 961; denominator >100,000,000 person-years |
| Wahlström, 2018[11]  Sweden  Cohort | Male construction workers who participated in a national occupational health surveillance program from 1971 to 1992, linked to Swedish Hospital Discharge Register  Follow-up: 1-32y  N=288,926  20-65y (NR)  ≥80% | Hospitalized LDH  Hospital  Primary diagnosis codes for LDH: 722.1 (“Displacement of thoracic or lumbar intervertebral disc without myelopathy,” ICD-9, 1987–1996) or M51.1 (“Lumbar and other intervertebral disc disorders with radiculopathy,” ICD-10, 1997-2003) | Low | Incidence of hospitalization due to LDH among construction workers from 1987 till 2010 was 1%  32y-cumulative incidence: 10.0 (9.6-10.3) per 1,000 persons  Average annual incidence: 0.04 (0.04-0.04) per 1,000 persons  Raw data: 2880/288,926 |
| Balling, 2019 [12]  Denmark  Cohort | Nationwide cohort on Danish adults followed till 2015 for hospitalization due to LBP or LDH from the Danish Health Examination Survey  Follow-up: 7.4y  N=46,826 (60%)  18-99y (48)  14% | Hospitalized LDH or sciatica  Hospital  LDH ICD-10 code M51.1 | Low | 7.4-year cumulative incidence of hospitalized LDH: 10.2 (9.4-11.2) per 1,000 persons  Average annual incidence: 1.4 (1.3-1.5) per 1,000 persons  Raw data: 479/46,826 |
| Brauer, 2020 [13]  Denmark  Cohort | Airport baggage handlers with first time hospital diagnosis or treatment for low back disorders from 1990-2012 with linkage to the National Patient Register and Civil Registration System  Follow-up: 0-22y  N=68,436 (0%)  <30->60 (NR)  100% | First-time hospitalisation with a diagnosis or surgical treatment for lumbar disc herniation  Hospital  LDH diagnosis codes: ICD-8 codes (72510, 72511), ICD-10 codes (M51.0, M51.0A, M51.1, M51.1A, M51.1B, M51.1C, M51.1D, M51.1E, M51.1F, M51.1I, M51.2, M51.2A, M51.2B, M51.2C, M51.2D, M51.2E, M51.2F)  LDH surgical procedure codes: Old surgical codes (77480, 82073, 82173), NOMESCO (ABC16, ABC26) | Low | Cumulative incidence of LDH: 32.8 (31.5-34.1) per 1,000 persons  Incidence density of LDH: 2.2 (2.1-2.3) per 1,000 person-years  Incidence density among baggage-handlers: 2.5 (2.1-3.0) per 1,000 person-years  Raw data: 2,244/68,436 persons, 2,244/1,020,756 person-years, baggage-handlers 118/47,228 person-years |
| Jung, 2020 [14]  Korea  Cohort | Nationwide cohort of Korean residents with LDH followed from 2008-2016 by the National Health Insurance Service  Follow-up: 9y  N=NR (NR)  0-80+y (NR)  100% | Hospitalized LDH  Hospital  Clinical LDH with imaging confirmation  ICD-10 codes M51.06, M51.16, M51.17, M51.26, M51.27, M51.86, M51.87, G83.4 as a main diagnosis | Low | Incidence densities ranged from 9.5 (8.9-10.0) per 1,000 person-years in 2008, to 9.8 (9.2-10.4) per 1,000 person-years in 2012, to 8.0 (7.4-8.5) per 1,000 person-years in 2016  Raw data: 472,245 in 2008, 537,577 in 2012, 478,697 in 2016, denominator NR |
| Hurme, 1983 [15]  Finland  Cohort | AII lumbar disc herniation surgeries performed in South-West Finland from 1975-1979, based on surgical department registers of the Turku University Central Hospital area (mean area population during study period, 455,000)  Follow-up: 5y  N=1,011 surgeries (44%)  15-80y among cases (42 at surgery)  79% incident surgery | LDH surgery  Surgical  No explicit case definition provided  Included all operations performed for LDH, classified as first operations and reoperations | Mod | 5-year cumulative incidence of first LDH surgery: 1.7 (1.6-1.8) per 1,000 persons  Average annual incidence: 0.3 (0.3-0.4) per 1,000 persons  Raw data: 778/455,000 |
| Heikkilä, 1989 [16]  Finland  Cohort | Finnish twin cohort consisting of 9,365 adult pairs of the same sex followed from 1972-1985 for hospitalized sciatica by record linkage to the National Hospital Discharge Register  Follow-up: 14y  N=18,730 (55%)  24-60y+ (NR)  100% | Hospitalized sciatica  Hospital  Sciatica was ICD-8 discharge diagnosis code: 353 | Mod | 14-year cumulative incidence of hospitalized sciatica among twins: 16.5 (14.8-18.5) per 1,000 persons  Average annual incidence: 1.2 (1.1-1.3) per 1,000 persons  Raw data: 304/18,370 |
| Riihimäki, 1989 [17]  Finland  Cohort | Male concrete reinforcement workers and house painters without prior history of sciatic pain in 1977 followed up in 1982  Follow-up: 5y  N=178 (0%)  25-54y at baseline  77-80% | Sciatica  Clinical  Sciatica was defined as back pain radiating to a leg in follow-up mailed questionnaire | Mod | 5-year cumulative incidence of sciatica: among concrete reinforcement workers, 343 (244-457) per 1,000 persons; among house painters, 229 (159-318) per 1,000 persons  Average annual incidence of sciatica: among concrete reinforcement workers, 68.5 (48.8-91.3) per 1,000 persons; among house painters, 45.7 (31.7-63.5) per 1,000 persons  Raw data: 25/73, 24/105 |
| Jørgensen, 1994 [18]  Denmark  Cohort | Occupationally active assistant nurses followed for LDH surgery in 1988 by record linkage to the Danish National Registry of Hospitalized Patients, and compared to all Danish females  Follow-up: 1y  N=1,681,152 (100%)  20-69y (NR)  100% | LDH surgery  Surgical  LDH surgery was ICD-8 surgical codes: 82073, *ablatio prolapsus disci intervertebralis lumbalis*; 82173, *evacuatio disci intervertebralis lumbalis* | Mod | 1-year cumulative incidence of LDH surgery among nurses aged 30-69y: 1.3 (1.0-1.8) per 1,000 persons  1-year cumulative incidence of LDH surgery among Danish women aged 30-69y: 0.78 (0.74-0.84) per 1,000 persons  Raw data: 37/28,008; 969/1,235,038 |
| Riihimäki, 1994 [19]  Finland  Cohort | Male machine operators, carpenters and office workers without prior history of sciatica in 1984, followed up in 1987  Follow-up: 3y  N=1,149 (0%);  25-49y (37)  83% | Sciatica  Clinical  Sciatica was LBP radiating to a leg in follow-up mailed questionnaire | Mod | 3-year cumulative incidence of sciatica: among office workers, 141 (111-177) per 1,000 persons; among machine operators, 220 (181-264) per 1,000 persons; among carpenters, 241 (198-290) per 1,000 persons  Average annual incidence of sciatic pain: among carpenters, 80.4 (66.1-96.5) per 1,000 persons; among machine operators, 73.2 (60.4-87.8) per 1,000 persons; among office workers, 46.9 (37.0-59.0) per 1,000 persons  Raw data: 81/336; 85/387; 60/426 |
| ^a^ Leino-Arjas, 2004 [20] ^b^ Leino-Arjas, 2002 [21]  Finland  Cohort | Nationwide workforce cohort followed in 1996 for lumbar intervertebral disc disorders by record linkage to the National Hospital Discharge Register  Follow-up: 1y  N=2,409,319 (NR)  20-64y (NR)  100%  ^a^ n=1,783,616 (51% women); 25-64y  ^b^ n=2,409,319 (NR); 20-64y | Hospitalized LDH and LDH surgery  Hospital and Surgical  Hospitalized LDH was primary diagnosis ICD-10 codes M51.1-M51.9 (intervertebral disc disorders other than those of the cervical spine)  LDH surgery was those with information on surgical decompression or spondylodesis of the lumbar spine during any of the admissions in 1996 | Mod | 1-year cumulative incidence of hospitalized LDH: among entire 20-64y workforce, 1.9 (1.9-2.0) per 1,000 persons; among those gainfully employed throughout prior year, 2.2 (2.1-2.2) per 1,000 persons; among men gainfully employed throughout prior year, 2.5 (2.4-2.7) per 1,000 persons; among women gainfully employed throughout prior year, 1.8 (1.7-1.9) per 1,000 persons  1-year cumulative incidence of LDH surgery: among entire 20-64y workforce, 1.0 (0.9-1.0) per 1,000 persons; among those gainfully employed throughout prior year, 1.1 (1.1-1.2) per 1,000 persons  Raw data: 4,643/2,409,319 entire workforce; 3,863/1,783,616 gainfully employed workforce; 2,211/868,876 in men gainfully employed; 1,652/914,740 in women gainfully employed; 2,368/2,409,319 surgical cases in entire workforce; 2,015/1,783,616 surgical cases in gainfully employed workforce |
| Leclerc, 2003 [22]  France  Cohort | Male workers in the French national electricity and gas company without LBP during the past 12 months in 1992 were followed up in 1994  Follow-up: 2y  N=841 (0%)  40-50y at baseline (NR)  65% | Sciatica  Clinical  Sciatica was pain, discomfort or stiffness in the low back region at least 1 day in the previous 12 months, with radiating symptoms in the leg, in follow-up mailed questionnaire | Mod | 2-year cumulative incidence of sciatica: 55.9 (42.3-73.5) per 1,000 persons; among those with a history of LBP before 1992, 101.9 (63.7-159.1) per 1,000 persons; among those with no history of LBP before 1992, 45.3 (32.1-63.6) per 1,000 persons  Average annual incidence of sciatica: 27.9 (21.1-36.8) per 1,000 persons; among those with a history of LBP before 1992, 51.0 (31.9-79.5) per 1,000 persons; among those with no history of LBP before 1992, 22.6 (16.1-31.8) per 1,000 persons  Raw data: 47/841; 16/157; 31/684 |
| ^a^ Jarvik, 2005 [23] ^b^ Suri, 2014 [24]  USA  Cohort | Outpatients without LBP or sciatica in the past 4 months from four clinics at the Veterans Affairs Puget Sound Health Care System, Seattle Division  Follow-up: 3y  N=148 (13%)  35-70y (median, 53)  89% | Clinical LDH and sciatica  Clinical  ^a^ LDH was MRI-confirmed disc protrusion or extrusion, with pain frequency for low back or buttock pain rated as more than “some of the time” [and] sciatic leg pain; or numbness or tingling in the leg, foot, or groin; or weakness in leg or foot, rated as more than “none”  ^b^ Incident sciatica (“radicular symptoms”) defined as any self-reported sciatica, lower extremity numbness or tingling, or lower extremity weakness, at one or more time points over the 3-year follow-up | Mod | ^a^ 3-year cumulative incidence of LDH: 89.4 (50.7-153.1) per 1,000 persons Average annual incidence: 29.8 (16.9-51.0) per 1,000 persons  ^b^ 3-year cumulative incidence of radicular symptoms: 569.1 (480.8-653.2) per 1,000 persons Average annual incidence: 189.7 (160.3-217.7) per 1,000 persons  Raw data: 11/123; 70/123 |
| ^a^ Sørensen, 2011 [25] ^b^ Jørgensen, 2013 [26]  Denmark  Cohort | Male workers in 1970 to 1971, at 14 private and public companies (railway, telephone, insurance, postal and firefighting) in Copenhagen, linked to Danish National Hospital Register (Copenhagen Male Study)  Follow-up: 6-33y  N=3,833 (0%)  40-59y (NR)  87% | Hospitalized LDH  Hospital  Hospitalization due to LDH identified in the National Hospital Register: ICD-8 code 725.11, from 1977 to 1994, and ICD-10 code M51.1, from 1994 to 2003. | Mod | ^a,b^ 26-year cumulative incidence of LDH: 16.7 (13.1-21.3) per 1,000 persons  Average annual incidence: 0.6 (0.5-0.8) per 1,000 persons  Raw data: 64/3,833 |
| Wahlström, 2012 [27]  Sweden  Cohort | Male construction workers who participated in a national occupational health surveillance program from 1971 to 1992, linked to Swedish Hospital Discharge Register  Follow-up: 1-32y  N=263,529 (0%)  20-65y (NR)  ≥80% | Hospitalized LDH  Hospital  Primary diagnosis codes for LDH: 722.1 (“Displacement of thoracic or lumbar intervertebral disc without myelopathy,” ICD-9, 1987–1996) or M51.1 (“Lumbar and other intervertebral disc disorders with radiculopathy,” ICD-10, 1997-2003) | Mod | Incidence density of hospitalized LDH among male construction workers: 0.6 (0.6-0.6) per 1,000 person-years  Incidence densities ranged from a low of 0.4 (0.4-0.5) per 1,000 person-years in white collar and foremen workers, to a high of 1.0 (0.6-1.5) per 1,000 person-years in refrigerator technicians  Raw data: 2,239/3,740,546 in male construction workers; 208/479,839 in white-collar and foremen; 17/17,655 in refrigerator technicians |
| Bovenzi, 2015 [28]  Italy  Cohort | Male professional drivers employed in several industries (marble quarries, marble laboratories, dockyards and paper mills) and public utilities (garbage services and public transport) in various provinces of Italy  Follow-up: 1-2y  N=598 (0%)  NR (41y)  90% | Sciatica  Clinical  Sciatic pain defined as LBP radiating to one or both legs below the knee, lasting one day or longer in the last 7 days and the previous 12 months | Mod | 1-year cumulative incidence of sciatica: among all drivers,167.6 (138.4-201.5) per 1,000 persons; among drivers of earth-moving machines in marble quarries and laboratories (group A), 217.7 (154.2-298.3) per 1,000 persons; among drivers of forklift trucks in marble laboratories, dockyards and paper mills (group B), 165.7 (117.2-229.0) per 1,000 persons; among drivers of buses in public transport and garbage machines in public services (group C), 143.4 (105.0-193.0) per 1,000 persons  Raw data: all drivers, 90/537; group A, 27/124; group B, 28/169; group C, 35/244 |
| Chan, 2018 [29]  Taiwan  Cohort | Physician and non-physician healthcare professionals with LDH linked to the Taiwan National Health Research Database  Follow-up: 5y  N= 115,488 (42%)  NR (47y)  100% | Clinical LDH  Clinical  ICD-9-CM diagnosis codes: 722.10, 722.52, 722.73, 722.93 | Mod | 5-year cumulative incidence of LDH 17.6 (16.8-18.4) per 1,000 persons  Average annual incidence of LDH 3.5 (3.4-3.7) per 1,000 persons  Raw data: 2,031/115,488 |
| Fouquet, 2018 [30]  France  Cohort | ^a^Lumbar disc surgery (LDS) study:  Patients with LDH surgery from 2007-2008 who responded to a questionnaire  Follow-up: 2y  N=1489 (NR)  20-59y (NR)  57%  ^b^Occupational disease - disc related sciatica (OD-DRS):  French workers compensated for disc related sciatica as a occupational disease living in the Pays de la Loire from 2009-2010  Follow-up: 2y  N=1009 (NR)  20-59y (NR)  NR | ^a^LDH surgery  Surgical  Surgical codes for hospital discharge database (LHPH907 LFFA002 LFFA003 LFFC002 LFFA011 LFFA010 LHKA900 )  ^b^Clinical LDH  Clinical  ICD-10 code M511  Chronic LBP associated with lumbar disc herniation with radiculopathy caused by vibration or manual handling of loads | Mod | ^a^2-year cumulative incidence of LDH surgery: men 0.5 (0.2-1.2) per 1,000 persons, in women 0.5 (0.2-1.2) per 1,000 persons  Average annual incidence of LDH surgery: men 0.3 (0.1-0.6) per 1,000 persons, women 0.3 (0.1-0.6) per 1,000 persons  ^b^2-year cumulative incidence of clinical LDH or disc-related sciatica: men 0.5 (0.2-1.2) per 1,000 persons, in women 0.2 (0.1-0.7) per 1,000 persons  Average annual incidence of clinical LDH or disc-related sciatica: men 0.3 (0.1-0.6) per 1,000 persons, women 0.1 (0.1-0.4) per 1,000 persons  Raw data: NR |
| Han, 2018 [31]  South Korea  Cohort | Nationwide cohort of Korean public officers (Police officer, firefighter, public educational officers) and national and regional government officers with claims data from 2002-2014 collected from the National Health Insurance Service  Follow-up: 13y  N=860,221 (36%)  NR (40y)  100% | Clinical LDH  Clinical  ICD-10 code M51 («lumbar disc herniation») | Mod | In men:  Incidence density: Police 23.6 (22.7-24.5), firefighter 25.5 (24.6-26.5), public educational officer 22.1 (21.2-23.0), national and regional government officer 21.1 (20.2-22.0) per 1,000 person-years  In women:  Incidence density: Police 21.1 (20.3-22.0), firefighter 26.6 (25.6-27-6), public educational officer 19.7 (18.9-20.6), national and regional government officer 19.9 (19.0-20.7) per 1,000 person-years  Raw data: numerator NR, denominator overall 10,017,374 person-years, in men 6,315,940 person-years, in women 3,701,434 person-years |
| Kim, 2018 [32]  Korea  Cohort | Nationwide cohort followed for LDH from 2004-2010 by record linkage to the National Health Insurance Service  Follow-up: 7y  N=18,786,256 (33%)  20-69y (NR)  100% | Clinical LDH  Clinical  The following diagnosis codes were used: M501, M510, MM11, M512 | Mod | 7-year cumulative incidence of LDH: 93.7 (93.0-94.3) per 1,000 persons, in men 80.4 (79.6-81.3) per 1,000 persons, in women 106.9 (105.9-107.9) per 1,000 persons, among regional subscribers 83.3 (82.1-84.5) per 1,000 persons, among company subscribers 96.1 (94.8-97.4) per 1,000 persons, among government office and private school subscribers 86.6 (85.4-87.8) per 1,000 persons, among medical care 108.8 (107.4-110.1) per 1,000 persons  Average annual incidence of LDH: 13.4 (13.3-13.5) per 1,000 persons  Raw data: 74,941/800,000, in men 32,171/400,000, in women 42,771/400,000 |
| Knox, 2018 [33]  USA  Cohort | Military helicopter pilots and matched active-duty military service members with first time diagnosis of lumbar disc displacement linked to the Defense Medical Epidemiology Database followed from 2006-2015  Follow-up: 10y  N=NR (NR)  20-40y+ (NR)  100% | First-time diagnosis with lumbar disc displacement  Clinical  ICD-9 code 722.1 (lumbar disc displacement) | Mod | Incidence density: helicopter pilots 8.6 (8.1-9.1) per 1,000 person-years, active-duty military members 7.0 (6.9-7.2) per 1,000 person-years  Incidence density in 1997 was 4.1 per 1,000 person-years, 2014 10.7 per 1,000 person-years  Raw data: helicopter pilots 1,218/141,383, non-pilots 15,111/2,147,246 |
| Huang, 2019 [34]  Taiwan  Cohort | Nationwide cohort of dentists, non-dentist healthcare professionals and age and sex matched participants from the general population without LDH before 2007 followed up till 2011 in the National Health Insurance Research Database  Follow-up: 5y  N=165,600 (36%)  ≤34-60y+ (43y)  100% | Clinical LDH  Clinical  ICD‐9‐CM codes: 722.10, 722.52, 722.73 and 722.93 | Mod | 5-year cumulative incidence of LDH among dentists: 14.0 (11.9-16.3) per 1,000 persons  5-year cumulative incidence of LDH among non-dentist healthcare providers: 15.9 (15.0-16.9) per 1,000 persons  5-year cumulative incidence of LDH in general population: 16.1 (15.2-17.0) per 1,000 persons  Raw data: dentists 150/10,734, non-dentist healthcare provider 1,147/72,066, general population 1,331/82,800 |
| Jäntti, 2022 [35]  Finland  Cohort | Patients presenting to the emergency department at Tampere University Hospital, Mikkeli Central Hospital or Central Finland Hospital during the year 2020  Follow-up  N=4,310 (55%)  NR (54y)  100%  N=NR | Hospital-based LDH (ED visits for LDH)  Hospital  LDH: ICD-10 codes M51.0 and M51.1 | Mod | Annual incidence of emergency department visits for LDH:  0.3 (0.2-0.5) per 1,000 persons  Other findings:  The incidence density of visits due to lumbar disc herniation remained the same during COVID compared to the reference years.  Raw data: numerator NR, 900,000 denominator |
| Nyrhi, 2023 [36]  Finland  Cohort | Nationwide cohort of Finnish women from the Finnish Care Register for Health and the Finnish Medical Birth Register with a lumbar discectomy between 1999 and 2017  Follow-up: 19y  N=13,912 (100%)  15-49y (NR)  100% | LDH surgery  Surgical  Nordic-Medico-Statistical Committee classification codes: ABC01, ABC04, ABC07, ABC10, ABC13, ABC16, ABC17, ABC20, ABC23, ABC26 | Mod | Incidence density of LDH surgery during pregnancy: 0.1 (0.1-0.2) per 1,000 person-years  Incidence density of LDH surgery within 12 months postpartum: 0.5 per 1,000 person-years  IRR of LDH surgery: not pregnant OR 1.0, during pregnancy 0.2 (0.1-0.2), first 12 months postpartum 0.7 (0.6-0.8)  Raw data: NR |
| Abbreviations: CT, computerized tomography; HNP, herniated nucleus pulposus; ICD, international classification of diseases; LBP, low back pain; LDH, lumbar disc herniation; Mod, moderate; MRI, magnetic resonance imaging; N, study size; NR, not reported; ROB, risk of bias; y, years | | | | |

**Appendix 5.** Admissible studies examining risk factors for LDH with radiculopathy in adults (n = 53)

| First author, Year of publication  Country  Study design | Participants and setting  Follow-up  N (% female)  Age range (mean)  Participation % | Study outcome  Case definition type  Case definition | ROB | Risk factors considered | Phase of evidence  Risk estimates (95% CI) |
| --- | --- | --- | --- | --- | --- |
| Jhawar, 2006 [7]  USA  Cohort | Female nurses from the Nurses’ Health Study without prior LDH in 1976, were followed up in 1992  Follow-up: 16 years  N=98,407 (100%)  30-55y in 1976 (NR)  ≥85% | Clinical LDH  Clinical  Self-reported physician-diagnosis of LDH that was confirmed by MRI or CT, in follow-up mailed questionnaire | Low | Cardiovascular risk factors: BMI, smoking, diabetes, high cholesterol, hypertension, family history of coronary heart disease | Phase III  High cholesterol: no RR 1.0; yes RR 1.3 (1.1-1.4)  Diabetes: no RR 1.0; yes RR 1.5 (1.2-2.0)  Hypertension: no RR 1.0; yes RR 1.3 (1.1-1.4)  Family history: no RR 1.0; yes RR 1.1 (1.0-1.3)  BMI (kg/m^2^): <21.9 RR 1.0; 22.0-24.9 RR 1.0 (0.9-1.1); 25.0-26.9 RR 1.1 (1.0-1.3); 27.0-28.9 RR 1.2 (1.0-1.4); 29+ RR 1.1 (1.0-1.3)  Smoking: non-smoker RR 1.0; ex-smoker RR 1.1 (1.0-1.2); current RR 1.4 (1.3-1.5) |
| Hincapié, 2018 [10]  Canada  Cohort | All adults with acute LDH requiring ED visit and early LDH surgery from April 1994 to December 2004, using population-based Ontario healthcare databases  Follow-up: 11 years  N=195 (40%), primary analysis; N=961(NR), sensitivity analyis  ≥18y (43y)  NR | Early LDH surgery  Surgical  Patients with at least one disc surgery intervention code and a recent primary care physician or chiropractic care visit due to LDH | Low | Chiropractic care and primary care physician visits | Phase III  IRR for acute LDH 0 to 7 days after visit with early surgery: chiropractic care 12.9 (7.2-23.3), primary care physician 14.5 (9.9-21.2)  IRR for acute LDH within 12-week emergency department window and early surgery (more sensitive outcome case definition): chiropractic care 10.0 (7.4-13.5), primary care physician 18.6 (15.0-23.0) |
| Wahlström, 2018 [11]  Sweden  Cohort | Male construction workers who participated in a national occupational health surveillance program from 1971 to 1992, linked to Swedish Hospital Discharge Register  Follow-up: 1-32y  N=288,926 (0%)  20-65y (NR)  ≥80% | Hospitalized LDH  Hospital  Primary diagnosis codes for LDH: 722.1 (“Displacement of thoracic or lumbar intervertebral disc without myelopathy,” ICD-9, 1987–1996) or M51.1 (“Lumbar and other intervertebral disc disorders with radiculopathy,” ICD-10, 1997-2003) | Low | Whole-body vibration (WBV), age, smoking | Phase III  WBV: white-collar workers and foremen RR 1.0, construction workers with none to very low WBV exposure RR 1.2 (1.1-1.4), construction workers with moderate to high WBV exposure RR 1.4 (1.1-1.6)  WBV in age group 30-49y: white-collar workers and foremen RR 1.0, construction workers with none to very low WBV exposure RR 1.5 (1.2-1.8), construction workers with moderate to high WBV exposure RR 1.7 (1.3-2.3) |
| Balling, 2019 [12]  Denmark  Cohort | Nationwide cohort on Danish adults followed till 2015 for hospitalization due to LBP or LDH from the Danish Health Examination Survey  Follow-up: 7.4y  N=46,826 (60%)  18-99y (48y)  14% | Hospitalized LDH or sciatica  Hospital  LDH ICD-10 code M51.1 | Low | Sex. total sitting time and physical activity during leisure time | Phase III  Physical activity during leisure time: inactive HR 1.1 (0.8-1.5), light HR 1.0, moderate HR 1.3 (1.0-1.6), vigorous HR 0.9 (0.5-1.5)  Sitting time 0 to <6 hours per day combined with physical activity in leisure time: Inactive HR 1.1 (0.9-1.5), light HR 1.0, moderate HR 1.1 (0.9-1.4) vigorous HR 1.6 (1.0-2.3)  Sitting time 6 to <10 hours per day combined with physical activity in leisure time: Inactive HR 1.2 (0.9-1.6), light HR 1.0, moderate HR 1.3 (1.1-1.6), vigorous HR 1.4 (0.9-2.1)  Sitting time 10+ hours per day combined with physical activity in leisure time: Inactive HR 1.0 (0.7-1.5), light HR 1.0, moderate HR 1.5 (1.0-2.1), vigorous HR 2.2 (1.1-4.2)  In men:  Total sitting time (hours per day): 0 to <6 HR 1.0, 6 to <10 HR 1.2 (0.9-1.7), 10+ HR 1.1 (0.7-1.7)  Physical activity during leisure time: Inactive HR 0.8 (0.5-1.3), light HR 1.0, moderate HR 1.4 (1.0-1.9), vigorous HR 0.7 (0.3-1.4)  In women:  Total sitting time (hours per day): 0 to <6 HR 1.0, 6 to <10 HR 0.9 (0.7-1.2), 10+ HR 0.8 (0.5-1.2)  Physical activity during leisure time: Inactive HR 1.4 (1.0-2.0), light HR 1.0, moderate HR 1.1 (0.8-1.6), vigorous HR 1.4 (0.7-3.1) |
| Brauer, 2020 [13]  Denmark  Cohort | Airport baggage handlers with first time hospital diagnosis or treatment for low back disorders from 1990-2012 with linkage to the National Patient Register and Civil Registration System  Follow-up: 0-22y  N=68,436 (0%)  <30-60y+ (NR)  68% | First-time hospitalisation with a diagnosis or surgical treatment for lumbar disc herniation  Hospital  LDH diagnosis codes: ICD-8 codes (72510, 72511), ICD-10 codes (M51.0, M51.0A, M51.1, M51.1A, M51.1B, M51.1C, M51.1D, M51.1E, M51.1F, M51.1I, M51.2, M51.2A, M51.2B, M51.2C, M51.2D, M51.2E, M51.2F)  LDH surgical procedure codes: Old surgical codes (77480, 82073, 82173), NOMESCO (ABC16, ABC26) | Low | Cumulative years of employment as a baggage-handler, BMI, education, marital status, smoking habits, alcohol consumption, leisure-time physical activity, general health | Phase III  Occupation: Non-baggage handler IRR 1.0, baggage handler IRR 0.8 (0.6-1.2)  Years of employment: 0.1-2.9y IRR 1.0, 3.0-9.9y IRR 1.1 (0.7-1.7), 10-19.9y IRR 1.2 (0.7-1.9), >20y IRR 1.1 (0.5-2.1) |
| ^a-c^ Heliövaara, 1987 [1–3]  Finland  Cohort with case-control risk analyses | Nationwide cohort of Finnish adults followed from 1970-1980 for hospitalized LDH or sciatica in the National Hospital Discharge Register  Follow-up: 11y  N=57,000 (48%)  ≥15y (NR)  83%  ^a^ n=2,732; 20-59y  ^b^ n=1,537; 20-59y  ^c^ n=2,732; 20-59y | Hospitalized LDH or sciatica  Hospital  ICD-8 codes indicating principal diagnosis for hospitalization: 725.10 or 725.19 for LDH; 353.99 for sciatica | Low | ^a^ Sex, age, geographic region, type of population, social class (men only), number of births (women only), marital status, leisure time physical activity, smoking, chronic cough, number of psychological distress symptoms, medication use, frequent use of analgesics  ^b^ Height, BMI (as an overall measure of obesity), and triceps skinfold thickness  ^c^ Occupation, strenuousness of work | Phase II  Hospitalized LDH  Sex: women RR 1.0; men RR 1.6 (p<0.001)  Age at baseline (y): 15-19 RR 1.0; 20-29 RR 3.0 (p<0.001); 30-39 RR 5.5 (p<0.001); 40-49 RR 6.5 (p<0.001); 50-59 RR 3.0 (p<0.001); 60+ RR 1.3  Type of population: urban RR 1.0; rural RR 0.8 (p<0.05); industrial RR 1.1  Hospitalized sciatica  Sex: women RR 1.0; men RR 1.3 (p<0.05)  Age at baseline (y): 15-19 RR 1.0; 20-29 RR 2.7 (p<0.05); 30-39 RR 8.6 (p<0.001); 40-49 RR 12.1 (p<0.001); 50-59 RR 7.3 (p<0.001); 60+ RR 4.3 (p<0.01)  Type of population: urban RR 1.0; rural RR 0.9; industrial RR 1.5 (p<0.001)  Case-control analyses in men  Hospitalized LDH  Social class: I OR 1.0; II OR 2.4 (p<0.05); III OR 2.6 (p<0.05); IV OR 2.0; V OR 1.2  Height (cm): ≤169 OR 1.0; 170-174 OR 1.1 (0.7-1.7); 175-179 OR 1.1 (0.7-1.7); ≥180 OR 2.3 (1.4-3.9)  BMI (kg/m^2^): <21.9 OR 1.0; 22.0-23.9 OR 2.4 (1.3-4.5); 24.0-25.9 OR 3.2 (1.7-6.1); 26.0-27.9 OR 3.1 (1.5-6.3); 28.0-29.9 OR 3.7 (1.7-8.0); ≥30.0 OR 2.3 (0.8-6.2)  Occupation: professional/white collar OR 1.0; intermediate non-manual OR 2.3 (p<0.05); forestry OR 2.9; farmer/agricultural OR 1.4; motor vehicle driver OR 2.9 (p<0.05); metal/machine worker OR 3.0 (p<0.01); construction worker OR 2.4 (p<0.05); chemical processor/paper worker OR 2.4; other industrial OR 2.2 (p<0.05); service and other groups OR 2.3  Hospitalized LDH or sciatica  Social class: I OR 1.0; II OR 3.2 (p<0.001); III OR 3.0 (p<0.001); IV OR 2.6 (p<0.01); V OR 1.5  Occupation: professional/white collar OR 1.0; intermediate non-manual OR 2.8; forestry OR 3.1 (p<0.05); farmer/agricultural OR 2.5 (p<0.01); motor vehicle driver OR 4.6 (p<0.001); metal/machine worker OR 4.2 (p<0.001); construction worker OR 3.1 (p<0.001); chemical processor/paper worker OR 3.2 (p<0.001); other industrial OR 2.6 (p<0.01); service and other groups OR 3.1 (p<0.01)  Case-control analyses in women  Hospitalized LDH  Height (cm): ≤159 OR 1.0; 160-164 OR 1.0 (0.6-1.6); 165-169 OR 1.2 (0.6-2.3); ≥170 OR 3.7 (1.6-6.6)  Number of psychological distress symptoms: 0 OR 1.0; 1 OR 2.0 (p<0.05); 2 OR 2.9 (p<0.001); 3-5 OR 1.2  Strenuousness of work: light or very light OR 1.0; normal OR 3.8 (p<0.05); heavy or very heavy OR 2.4  Hospitalized LDH or sciatica  Number of psychological distress symptoms: 0 OR 1.0; 1 OR 1.7 (p<0.05); 2 OR 2.0 (p<0.01); 3-5 OR 1.8 (p<0.05)  Medication use: None OR 1.0; Some OR 0.7 (p<0.05)  Frequent use of analgesics: No OR 1.0; Yes OR 1.9 (p<0.01)  Strenuousness of work: light or very light OR 1.0; normal OR 2.0 (p<0.05); heavy or very heavy OR 2.5 (p<0.05) |
| Zitting, 1998 [5]  Finland  Cohort | Birth cohort from 2 provinces of Finland followed from 1966-1994 for hospitalized LDH in the National Hospital Discharge Register  Follow-up: 28 years  N=12,058 (NR)  15-28y among cases (NR)  92% | Hospitalized LDH  Hospital  ICD-9 diagnosis codes and corresponding ICD-8 codes used to identify all possible lumbar disc disease cases: 7221, 7227, 7244, 7225, 7242, 7245, 7561, 7384, 7385, 7213, 7214  “Confirmed” LDH was a reliable description of herniation in a surgical report or reliable evidence of a herniation on MRI, CT or myelogram with appropriate symptoms (i.e., on the same side) described in the medical records | Low | Sex, age | Phase II  Sex: women RR 1.0; men RR 2.2 (1.3-3.6)  Age: NR; in men, hospitalized LDH first occurred around 15y of age, and incidence rose more sharply from 20y of age; in women, first cases of hospitalized LDH also appeared around 15y of age |
| Miranda, 2002 [6]  Finland  Cohort | Forest industry workers who had no sciatica during the past 12 months in 1994  Follow-up: 1y  N=2,077 (26%)  NR (45)  77% | Sciatica  Clinical  Sciatica was more than 7 days of low back pain radiating below the knee during the preceding 12 months, with a manikin used to denote the anatomic area | Low | Individual: sex, age, height, BMI, mental stress, smoking, car driving, previous low back injuries  Physical exercise: frequency of physical exercise, sports activity, different types of sports  Work-related: amount of twisting movements of trunk, working with trunk forward flexed, working with a hand above shoulder level, working in sitting position, working in kneeling or squatting position, daily lifting of loads, operating a motor vehicle, physical strenuousness of work, job satisfaction, overload at work, risk of accident at work | Phase II  Sex: male OR 1.0, female OR 0.9 (0.5-1.6)  Age (y): <35 OR 1.0; 35-44 OR 2.1 (1.1-4.1); 45-54 OR 2.0 (1.0-4.0); ≥55 OR 3.3 (1.4-7.6)  Smoking: non-smoker OR 1.0; ex-smoker OR 1.3 (0.9-2.0); current smoker (1-15cig/d, 1-15y) OR 1.3 (0.6-3.1); current smoker (>15cig/d, 1-15y) OR 1.2 (0.3-4.6); current smoker (1-15cig/d, >15y) OR 2.2 (1.2-4.0); current smoker (>15cig/d, >15y) OR 2.3 (1.3-3.9)  Mental stress: not at all OR 1.0; only little OR 1.6 (0.9-2.9); to some extent OR 2.1 (1.2-3.7); rather much or much OR 3.0 (1.5-5.9)  Walking: not at all or only little OR 1.0; moderately OR 1.8 (1.2-2.8); actively OR 1.9 (1.2-3.0)  Jogging: not at all or only little OR 1.0; moderately or actively OR 0.5 (0.3-1.0)  Twisting movements of trunk during the work day: not at all or only little OR 1.0; moderately OR 1.6 (1.1-2.5); much OR 1.9 (1.1-3.2) |
| Mattila, 2008 [8]  Finland  Cohort | Nationwide cohort of adolescents aged 14-18y, without LDH and prior hospitalized nonspecific back-related diagnosis followed up to December 31, 2001, in the National Hospital Discharge Register  Follow-up: 651,027 person-years (11y average follow-up)  N=57,408 (54%)  15-41y among cases (27 at surgery)  79% | LDH surgery  Surgical  Lumbar discectomy defined as ICD code 9211 between 1979 and 1996, and then with new codes ABC07, ABC16 and ABC26 from 1997 on | Low | Sex, socioeconomic background, perceived health status, chronic disease or disability, number of stress symptoms per week, timing of puberty, overweight, smoking, drinking style, frequency of participation in sports clubs, frequency of other leisure-time physical exercise, school success | Phase II  Sex: women RR 1.0; men RR 2.2 (1.7-2.9)  In men  Smoking: not daily HR 1.0; daily HR 1.5 (1.1-2.2)  Timing of puberty: early HR 1.0; normal HR 0.7 (0.5-1.1); late HR 0.6 (0.4-1.0)  In women  Overweight: no HR 1.0; yes HR 2.1 (1.1-4.1)  Frequency of participation in sports clubs: never HR 1.0; 2-3 times per week or less HR 1.5 (0.9-2.5); 4-5 times per week or more HR 2.7 (1.1-6.3) |
| Jung, 2020 [14]  Korea  Cohort | Nationwide cohort of Korean residents with LDH followed from 2008-2016 by the National Health Insurance Service  Follow-up: 9y  NR (NR)  0-80y+ (NR)  100% | Hospitalized LDH  Hospital  Clinical LDH with imaging confirmation  ICD-10 codes M51.06, M51.16, M51.17, M51.26, M51.27, M51.86, M51.87, G83.4 as a main diagnosis | Low | Age, sex | Phase II  Other findings:  The crude incidence of LDH in female patients exceeded that of male patients in their middle age (30s or 40s) and was 1.5–1.6 times higher than in male patients in their 60s |
| Seidler, 2003 [37]  Germany  Case-control | Cases: 225 male patients with acute LDH (94 LDH only; 131 LDH and osteochondrosis or spondylosis) from 2 orthopaedic practices and 4 neurosurgical or orthopaedic clinics in the Frankfurt/Main area  Controls: 107 population controls without chronic LBP and 90 patients hospitalized for urolithiasis treatment by lithotripsy without chronic LBP and osteochondrosis/ spondylosis (197 controls in total)  Excluded persons with Bechterew’s disease, spine fractures, malignancies of the spine, and poliomyelitis  Follow-up: NA  437 (0%)  25-65y (42)  66-93% | Clinical LDH  Clinical  LDH only cases defined as currently symptomatic, radiographically confirmed (MRI and/or CT scan) LDH or protrusion | Mod | Occupational: physical workload, lifting/carrying, extreme forward bending postures, whole body vibration, psychosocial work environment (monotonous, boring, opportunities to use knowledge and skills, information about future plans, satisfaction with supervisor, satisfaction with workmates, psychic strain through contact with clients, time pressure, too much responsibility) | Phase III  Physical workload: always working in occupations with low physical workload OR 1.0; ≥10y in occupations with medium physical workload OR 0.8 (0.4-1.7); ≥10y in occupations with high physical workload OR 2.1 (0.9-4.6)  Cumulative extreme (>90° trunk flexion) forward bending (h): 0 OR 1.0; >0-1,500 OR 1.4 (0.7-2.8); >1,500 OR 2.7 (1.2-6.4)  Cumulative whole body vibration (h): 0 OR 1.0; >0-1,800 OR 2.1 (0.9-4.8); >1,800 OR 1.9 (0.7-4.9)  Psychosocial time pressure (number of working years with high degree of time pressure, classified as 5 or 6): 0 OR 1.0; >0-<10 OR 1.2 (0.6-2.6); ≥10 OR 2.9 (1.3-6.3) |
| Huang, 2019 [34]  Taiwan  Cohort | Nationwide cohort of dentists, non-dentist healthcare professionals and age and sex matched participants from the general population without LDH before 2007 followed up till 2011 In the National Health Insurance Research Database  Follow-up: 5y  N=165,600 (36%)  ≤34-60y+ (43y)  100% | Clinical LDH  Clinical  ICD‐9‐CM codes: 722.10, 722.52, 722.73, and 722.93 | Mod | Occupation, age, sex | Phase III  General population OR 1.0, dentists OR 0.8 (0.6-1.0)  General population OR 1.0, non-dentist healthcare provider OR 1.1 (1.0‐1.2)  Non-dentist healthcare provider OR 1.0, dentist 0.8 (0.7‐1.0)  Age group ≤34y: general population OR 1.0, non-dentist healthcare provider OR 1.3 (1.1-1.6)  In men:  Non-dentist healthcare provider OR 1.0, dentist OR 0.8 (0.7-1.0) |
| ^a^ Leino-Arjas, 2004 [20] ^b^ Leino-Arjas, 2002 [21]  Finland  Cohort | ^a^ Nationwide occupationally active workforce cohort followed in 1996 for lumbar intervertebral disc disorders by record linkage to the National Hospital Discharge Register  Follow-up: 1y  N=1,783,616 (51%)  25-64y (NR)  100%  ^b^ Nationwide workforce cohort followed in 1996 for lumbar intervertebral disc disorders by record linkage to the National Hospital Discharge Register  Follow-up: 1y  N=2,409,319 (NR)  20-64y (NR)  100% | ^a,b^ Hospitalized LDH  Hospital  Hospitalized LDH was primary diagnosis ICD-10 codes M51.1-M51.9 (intervertebral disc disorders other than those of the cervical spine) | Mod | ^a^ Sex, age, education, income, smoking and BMI  Occupational: physical workload, manual materials handling, accident risk, inconvenient work postures, sedentary work, video display terminal work, challenging tasks, social demands, job control, work time schedule  ^b^ Sex, age, employment status, education, occupational class, income | ^a^ Phase III  Sex: women RR 1.0; men RR 1.4 (1.3-1.5)  In men  Age (y): 25-34 RR 1.0; 35-44 RR 1.4 (1.2-1.5); 45-54 RR 1.3 (1.2-1.5); 55-64 RR 1.1 (0.9-1.3)  Education: higher RR 1.0; intermediate RR 1.4 (1.2-1.6); basic RR 1.7 (1.4-1.9)  Personal income: highest quintile RR 1.0; 2^nd^ RR 1.2 (1.0-1.5); 3^rd^ RR 1.5 (1.3-1.8); two lowest quintiles combined RR 1.4 (1.2-1.6)  Accident risk: no RR 1.0; low RR 1.2 (1.0-1.5); high RR 1.5 (1.2-1.8)  Job control: low RR 1.0; high RR 0.9 (0.8-1.0)  In women  Age (y): 25-34 RR 1.0; 35-44 RR 1.8 (1.5-2.0); 45-54 RR 1.7 (1.5-2.0); 55-64 RR 1.2 (1.0-1.5)  Education: higher RR 1.0; intermediate RR 1.6 (1.4-1.8); basic RR 1.8 (1.5-2.1)  Personal income: highest quintile RR 1.0; 2^nd^ RR 1.5 (1.3-1.8); 3^rd^ RR 1.5 (1.2-1.8); two lowest quintiles combined RR 1.5 (1.2-1.8)  BMI: lowest tertile RR 1.0; middle tertile RR 1.1 (1.0-1.2); highest tertile RR 1.3 (1.1-1.5)  Accident risk: no RR 1.0; low RR 1.3 (1.1-1.5); high RR 1.4 (1.2-1.8)  Job control: low RR 1.0; high RR 0.8 (0.8-0.9)  Work time schedule: regular daytime work RR 1.0; two-shift work, regular evening work, weekend work or other irregular work hours not including night work RR 1.1 (0.9-1.3); regular or irregular three-shift work or regular night work RR 1.3 (1.2-1.6)  ^b^ Phase II  Hospitalized LDH (crude analysis)  Age (y): 20-34 RR 1.0; 35-44 RR 1.9 (1.7-2.0); 45-54 RR 1.8 (1.6-1.9); 55-64 RR 0.9 (0.8-1.1)  Employment status at end of year 1995: occupationally active RR 1.0; unemployed RR 0.8 (0.7-0.8); on unemployment pension RR 0.8 (0.5-1.1); on other pension RR 1.7 (1.6-1.9); student, at military service or other RR 0.5 (0.4-0.5)  Hospitalized LDH in those occupationally active throughout 1995 (multivariable analysis)  Sex: men RR 1.0; women RR 0.8 (0.8-0.9)  Education: higher RR 1.0; secondary RR 1.3 (1.2-1.5); basic RR 1.5 (1.3-1.8)  Occupational class: upper white-collar RR 1.0; lower white-collar (in supervising position or with independent work tasks) RR 1.0 (0.8-1.1); lower white-collar (in dependent position or with routine work tasks) RR 1.2 (1.0-1.4); specialized manual workers RR 1.4 (1.2-1.6); non-specialized manual workers RR 1.5 (1.3-1.8); farmers RR 1.3 (1.1-1.5); other entrepreneurs RR 1.2 (1.0-1.4)  Income: highest quartile RR 1.0; 2^nd^ RR 1.0 (1.0-1.1); 3^rd^ RR 1.0 (0.9-1.1); lowest RR 0.7 (0.6-0.8) |
| ^a^ Seidler, 2009 [38] ^b^ Bergmann, 2017 [39] ^c^ Schumann, 2010 [40] ^d^ Seidler, 2011 [41]  Germany  Case-control | ^a,b,c,d^ Cases: 915 patients with structural lumbar disc diseases (564 with LDH and sciatica; 351 with severe lumbar disc space narrowing) from four study regions in Germany  ^a, c, d^ Controls: 901 persons randomly selected from a 1% random sample of residents from local population registration offices of the same 4 regions  ^b^ Controls: 233 German residents with LBP and 422 without LBP in the past 12 months  Excluded those with Bechterew’s disease, inflammatory spine diseases, tumors of the spine or initial diagnosis of lumbar disc disease over 10 years ago  Follow-up: NA  ^a, c, d^ N=1,816 (50%)  ^b^ N=1,570 (51%)  ^a, c, d^ 25-70y (48y)  ^b^ 25-70y (51y among cases)  ^a, c, d^ 53-66%  ^b^ 66% cases, 54% controls | LDH hospital treatment  Hospital  Inpatient or outpatient hospital treatment because of LDH with radiculopathy, sensory and/or motor deficits (neurological findings), and confirmation of LDH by MRI or CT scan | Mod | ^a^ Occupational: cumulative lumbar load by manual materials handling (lifting, carrying, pushing, pulling, throwing, catching or shoveling of objects weighing ≥5 kg), cumulative lumbar load by intensive-load postures (postures with trunk inclination of ≥20 degrees)  ^b^ Cumulative lumbar load through manual materials handlingand/or trunk inclination >20°, and cumulative whole-body vibration dose due to vertical vibrations (in men only)  ^c^ Lifestyle factors: BMI, smoking, and sports activities (endurance sports, ball sports, athletic sports, body building sports)  ^d^ Age | ^a^ Phase III  In men  Cumulative lumbar load by manual materials handling and/or intensive-load postures (Nh):  0-<5.0*10^6^ OR 1.0; 5.0-<21.51*10^6^ OR 1.7 (1.1-2.7); ≥21.51*10^6^ OR 3.4 (2.2-5.0)  Cumulative lumbar load by manual materials handling (Nh): 0-<2.34*10^6^ OR 1.0; 2.34-<8.98*10^6^ OR 1.2 (0.7-2.0); ≥8.98*10^6^ OR 2.0 (1.2-3.5)  Cumulative lumbar load by intensive-load postures (Nh): 0 OR 1.0; >0-<4.85*10^6^ OR 1.1 (0.6-2.0); 4.85-14.62*10^6^ OR 1.7 (0.9-3.2); ≥14.62*10^6^ OR 1.9 (1.0-3.5)  In women  Cumulative lumbar load by manual materials handling and/or intensive-load postures (Nh): 0 OR 1.0; 0-<4.04*10^6^ OR 1.6 (1.1-2.7); 4.04-<14.47*10^6^ OR 2.4 (1.6-3.8); ≥14.47*10^6^ OR 2.3 (1.5-3.6)  Cumulative lumbar load by intensive-load postures (Nh): 0 OR 1.0; >0-<2.77*10^6^ OR 1.9 (1.0-3.7); 2.77-8.83*10^6^ OR 2.4 (1.2-4.6); ≥8.83*10^6^ OR 3.2 (1.6-6.3)  ^b^ Phase III  In men:  Manual materials handling and/or trunk inclination >20° in Nh:  0 to <5.0*10^6^ OR 1.0 5.0 to <21.51*10^6^ OR 1.9 (1.1-3.0) ≥21.51*10^6^ OR 3.7 (2.3-6.0)  Manual materials handling in Nh:  0 to <2.34*10^6^ OR 1.0 2.34 to <8.98*10^6^ OR 1.4 (0.8-2.6) ≥8.98*10^6^ OR 2.2 (1.2-4.1)  Trunk inclination >20° in Nh:  0 OR 1.0, >0 to <4.85*10^6^ OR 1.1 (0.6-2.1) 4.85 to 14.62*10^6^ OR 1.7 (0.8-3.4) >14.62*10^6^ OR 2.4 (1.2-5.0)  Whole-body vibration dose due to vertical vibrations in (m/s^2^)^2^:  0 OR 1.0, >0 to <364 OR 1.6 (0.5-4.9) 364 to <1190 OR 0.7 (0.3-1.6) ≥1190 OR 1.8 (0.4-9.0)  In women:  Manual materials handling and/or trunk inclination >20° in Nh:  0 OR 1.0  >0 to <4.04*10^6^ OR 2.2 (1.3-3.8),  4.04 to <14.47*10^6^ OR 3.6 (2.1-6.1)  ≥14.47*10^6^ OR 3.5 (2.0-5.9)  Manual materials handling in Nh:  0 OR 1.0 >0 to <1.58*10^6^ OR 0.8 (0.4-1.8) 1.58 to <9.06*10^6^ OR 1.1 (0.5-2.5) ≥9.06*10^6^ OR 1.1 (0.5-2.5)  Trunk inclination >20° in Nh:  0 OR 1.0 >0 to <2.77*10^6^ OR 2.7 (1.2-6.3) 2.77 to 8.83*10^6^ OR 2.6 (1.2-6.0) ≥8.83*10^6^ OR 3.7 (1.6-8.6)  ^c^ Phase II  In men  BMI (kg/m^2^): <21.88 OR 1.0; ≥21.88-<24.30 OR 1.4 (0.8-2.4); ≥24.30-<29.21 OR 2.1 (1.3-3.6); ≥29.21 OR 1.6 (0.7-3.8)  Smoking (pack-years): 0 OR 1.0; >0-<8 OR 1.0 (0.6-1.7); ≥8-<20 OR 1.2 (0.8-1.9); ≥20-<40 OR 1.6 (1.0-2.5); ≥40 OR 0.8 (0.4-1.5)  Cumulative hours of body building sports (h): 0 OR 1.0; >0-<400 OR 1.0 (0.5-2.4); 400-<1,350 OR 1.0 (0.5-2.2); ≥1,350 OR 0.5 (0.2-1.1)  In women  BMI (kg/m^2^): <21.88 OR 1.0; ≥21.88-<24.30 OR 1.3 (0.9-1.8); ≥24.30-<29.21 OR 1.3 (0.8-1.9); ≥29.21 OR 2.0 (1.1-3.7)  Smoking (pack-years): 0 OR 1.0; >0-<8 OR 1.0 (0.7-1.6); ≥8-<20 OR 1.5 (1.0-2.4); ≥20-<40 OR 1.0 (0.6-1.7); ≥40 OR 1.4 (0.4-4.5)  ^d^ Phase II  In men  Age (y): <35 OR 1.0; 35-44 OR 2.1 (1.3-3.7); 45-54 OR 1.9 (1.1-3.4); 55-64 OR 1.3 (0.8-2.4); ≥65 OR 1.4 (0.7-2.7)  In women  Age (y): <35 OR 1.0; 35-44 OR 0.7 (0.4-1.1); 45-54 OR 0.9 (0.6-1.5); 55-64 OR 0.9 (0.5-1.5); ≥65 OR 1.4 (0.7-2.6) |
| ^a-e^ Kelsey, 1975 [42–46]  USA  Case-control | Cases: 223 patients with surgical, probable or possible LDH after having lumbar spine x-rays taken in 3 area hospitals or in the office of 2 private radiologists between 1971-1973, from New Haven, Connecticut  Controls (matched): 217 patients admitted to the same hospital service or radiologist office for a condition not related to the spine, matched on age, sex, and medical setting  Controls (unmatched): 494 patients who had lumbar spine x-rays and were not classified as cases  Excluded those who had previous LDH or other serious back problems, and all persons who had experienced symptoms for ≥1year prior to study entry  Follow-up: NA  934 (41%)  20-64y (39 among cases)  78%  ^a, b, c, d^ n=934; 41% female  ^e^ n=407; 100% female | Combined clinical, hospitalized and surgical LDH  Clinical, hospital and surgical  Surgical cases were those in which: i) the hospital chart indicated that the surgeon saw a herniated disc during surgery (descriptions included ruptured, free fragments, herniated, prolapsed, bulging and extruded, but not disc degeneration without evidence of nerve root involvement); and ii) the patient reported pain distributed along the sciatic nerve; and iii) the patient had a positive straight leg raising test, and/or increased pain in the low back or along the sciatic nerve when stretching or extending the leg from a sitting position, and/or increased pain along the sciatic nerve when coughing, sneezing, or straining at stools  Probable cases were similar to surgical cases except that a herniation need not have been observed at surgery; and included cases in which the sciatic pain was felt in both the thigh and lower leg, and cases in which there was sciatic pain in part of the leg and numbness in another part  Possible cases differed from probable cases in that the sciatic pain was only in the thigh or the lower leg but not in both; or if the leg was numb but straight leg raising increased LBP | Mod | ^a,b^ Sex, age, race, social class, residence, marital status, number of children  ^b^ Month of onset of symptoms, height, weight, body bulk (BMI for men, weight/height for women), weight gain/loss in previous year, respiratory symptoms (chronic phlegm, chronic bronchitis), smoking, physical activity and sports, stressful life events in the previous year  ^b,c^ Occupational: sedentary jobs (sitting ≥half the time), jobs requiring driving (sitting ≥half the time in a motor vehicle; men only), truck driving (men only), jobs involving any lifting, jobs involving any pushing, jobs involving any pulling, jobs involving any carrying, heavy lifting  ^b,d^ Driving: jobs requiring driving (sitting ≥half the time in a motor vehicle; men only), truck driving (men only), driving other than on job  ^b,e^ Pregnancy: average number of pregnancies, pregnancies resulting in live births, pregnancies not resulting in live births | Phase II  All cases combined  Sex: NR; among surgical cases, male to female ratio 2.1 (p<0.001); among probable and possible cases, male to female ratio 1.1 (p>0.10)  Age: NR; LDH most common in the 30-49y age groups  Residence (matched analysis): urban OR 1.0; suburban OR 1.5 (1.0-2.4)  Sedentary jobs, age ≥35y (matched analysis) OR 2.4 (1.3-4.7)  Jobs requiring driving (matched analysis) OR 2.8 (1.2-7.1)  Truck driving job (matched analysis) OR 4.7 (1.3-25.3)  Driving other than on job, drivers v nondrivers: (in men; matched analysis) OR 2.7 (1.0-8.3); (in women; matched analysis) OR 1.9 (0.9-4.1); (in both men and women; matched analysis) OR 2.2 (1.2-3.9)  Chronic phlegm (in women; matched analysis) OR 2.8 (1.1-8.8)  Chronic bronchitis (in women; matched analysis) OR 3.7 (1.0-20.5)  Physical activity (matched analysis) OR 0.4 (0.2-1.0)  Pregnancy: NR; cases had more pregnancies resulting in live births than other women of their age  Other findings  Social class: NR; some indication of an association between higher social class and LDH in women, but no association in men  Sports: NR; some tendency for cases to play sports rarely and controls more frequently  Lifting on the job: no association found with heavy lifting or with frequent lifting |
| ^a,b^ Kelsey, 1984 [47, 48]  USA  Case-control | Cases: 325 patients with surgical, probable or possible LDH after having lumbar spine x-rays or myelograms taken in 3 area hospitals, 1 neurosurgical practice, or 2 orthopaedic practices between 1979-1981, from the New Haven and Hartford, Connecticut areas  Controls: 241 patients admitted to the same hospital service or practice for a condition not related to the spine, matched on age, sex, and medical setting  Excluded those who had previous prolapsed lumbar or cervical disc, or previous neck, back, leg or arm problems that caused activity restriction for more than 4 consecutive weeks, and all persons who had experienced symptoms for ≥1year prior to study entry  Follow-up: NA  566 (45%)  20-64y (NR)  72-79% | Combined clinical, hospitalized and surgical LDH  Clinical, hospital and surgical  Surgical cases were those in which: i) the hospital chart indicated that the surgeon saw a herniated disc during surgery (descriptions included ruptured, free fragments, herniated, prolapsed, bulging and extruded, but not disc degeneration without evidence of nerve root involvement); and ii) the patient reported pain distributed along the sciatic nerve; and iii) the patient had a positive straight leg raising test, and/or increased pain in the low back or along the sciatic nerve when stretching or extending the leg from a sitting position, and/or increased pain along the sciatic nerve when coughing, sneezing, or straining at stools  Probable cases were similar to surgical cases except that a herniation need not have been observed at surgery; and included cases in which the sciatic pain was felt in both the thigh and lower leg, and cases in which there was sciatic pain in part of the leg and numbness in another part  Possible cases differed from probable cases in that the sciatic pain was only in the thigh or the lower leg but not in both; or if the leg was numb but straight leg raising increased LBP | Mod | ^a^ Smoking, motor vehicle type, size or model, driving pattern characteristics (local roads, highways, bucket seats, regular seats, driver, passenger, automatic transmission, manual transmission), occupational lifting while twisting body, race, education, marital status, residence, height, weight, number of pregnancies, number of children, respiratory symptoms (chronic phlegm, chronic bronchitis), vibration, frequency of wearing shoes with high heels, participation in baseball, golf, bowling, swimming, diving from a board, tennis, bicycling, or jogging  ^b^ Occupational: frequency of lifting, frequency of carrying, frequency of twisting at waist, lifting while twisting body, holding while twisting body, bending knees while lifting and twisting | Phase II  All cases combined  Smoking: never smoked OR 1.0; former smoker (not in the past year) OR: 1.0 (0.6-1.7); current smoker (in past year) OR 1.7 (1.0-2.5)  Smoking (average 10cigs/d): OR per one unit increase, 1.2 (1.0-1.4)  Motor vehicle type (driven during the past 5y period): only Japanese or Swedish OR 1.0; other OR 3.0 (1.1-8.3)  Driving non-Japanese or non-Swedish car (average 5h/wk): OR per one unit increase, 1.3 (1.1-1.6)  Married v not married (in women) OR 2.2 (1.0-5.1)  Rural residence v urban or suburban residence OR 1.7 (0.9-3.3)  Lifting objects >11.3kg, >25 times per day while twisting body: knees bent (yes v no) OR 1.9 (0.8-4.8); knees not bent (yes v no) OR 7.2 (2.0-25.8);  Lifting objects >11.3kg while twisting body (with and without knees bent): no lifting while twisting body OR 1.0; lifting while twisting body, knees bent OR 2.7 (0.9-7.9); lifting while twisting body, knees almost straight OR 6.1 (1.3-27.9)  Frequency of lifting objects >11.3kg: not at all OR 1.0; <5 times/d OR 1.2 (0.7-2.0); 5-25 times/d OR 1.3 (0.7-2.5); >25 times/d OR 3.5 (1.5-8.5)  Frequency of carrying objects >11.3kg: not at all OR 1.0; <5 times/d OR 1.0 (0.6-1.9); 5-25 times/d OR 2.1 (1.0-4.3); >25 times/d OR 2.7 (1.2-5.8)  Lifting objects >11.3kg while twisting body: never or small amount OR 1.0; moderate amount OR 2.5 (0.9-6.8); large amount OR 3.1 (1.3-7.5) |
| Riihimäki, 1989 [17]  Finland  Cohort | Male concrete reinforcement workers and house painters without prior history of sciatic pain in 1977 followed up in 1982  Follow-up: 5y  N=178 (0%)  25-54y at baseline  77-80% | Sciatica  Clinical  Sciatica was defined as back pain radiating to a leg in follow-up mailed questionnaire | Mod | Occupation, previous back symptoms, earlier back accidents, lumbar spine degeneration, back muscle strength, abdominal muscle strength, height, BMI, stress, smoking | Phase II  Occupation: house painters OR 1.0; concrete reinforcement workers OR 1.8 (1.2-2.9)  Earlier back accidents: no OR 1.0; yes OR 1.6 (1.0-2.7)  Previous back symptoms: no symptoms OR 1.0; lumbago or nonspecific low back pain OR 1.8 (0.9-3.4)  BMI (kg/m^2^): ≤23.9 OR 1.0; 24.0-27.9 OR 1.1 (0.6-2.1); ≥28.0 OR 1.6 (0.8-3.1) |
| ^a.b^ Mundt, 1993 [49, 50]  USA  Case-control | Cases: 297 patients with confirmed and unconfirmed LDH between 1986-1988, from 38 orthopaedic and neurosurgical practices and 5 hospitals in Massachusetts, New Jersey, and New York  Controls: 287 patients admitted to the same hospital service or practice for a condition not related to the back or neck, matched on age, sex, geographic location, and source of medical care  Excluded those who had experienced symptoms for >1 year before index date; those who had previous disc surgery >1 year before index date; those who had other conditions of the back or neck; and those who had experienced activity limitation >1y before index date from back, leg, neck or arm pain of ≥4 weeks duration  Follow-up: NA  585 (41%)  20-64y (NR)  76-79% | Combined clinical, hospitalized and surgical LDH  Clinical, hospital and surgical  Confirmed case was herniation, prolapse, rupture, protrusion, extrusion, extradural defect, or free fragment noted on the surgical report, myelogram, CT scan, or MRI as reported in the medical record  Unconfirmed case was probable or possible herniation based on signs and symptoms consistent with herniation  Probable case was pain, numbness or tingling radiating to the hip, buttock, thigh or below the knee in a pattern consistent with nerve root impingement by the disc, with worsening of the symptoms with coughing, stretching the leg, or straining while moving the bowels  Possible case differed from probable LDH in that symptoms did not worsen with cough, stretch or strain; or symptoms referred to the thigh, and worsened with cough, stretch or strain, or positive straight leg raising indicated in the medical record; or symptoms were in the lower leg only, and worsened with cough, stretch or strain, or positive straight leg raising | Mod | ^a^ In 2y before index date  Occupational activities, use of motor vehicles, whole body and arm vibration, riding in planes, trains, buses, subways and motorcycles, nonoccupational lifting (inanimate objects and children), carrying, stretching, bending, shovelling, pregnancy history, smoking  Lifetime history  Occupational lifting, bending, and twisting  ^b^ In 2y before index date  Participation in specific sports (baseball or softball, golf, bowling, swimming, diving from a board at a height of at least 3 feet, jogging, aerobics, racquet sports), use of free weights, use of weightlifting equipment, warming up before workout | Phase II  All cases  Smoking (average 10cigs/d): OR per one unit increase, 1.4 (1.2-1.6)  Shovelling: no OR 1.0; yes, 5-15 times OR 0.8 (0.6-1.2); yes, >15 times OR 0.7 (0.4-1.2)  Repeated bending while doing off the job activities: no OR 1.0; yes, ≥2 days per week OR 1.4 (0.9-2.2)  Non-occupational lifting ≥11.3kg inanimate objects: no OR 1.0; yes, ≥1/wk for 6mo OR 1.1 (0.8-1.6); yes, knees bent, back straight OR 0.6 (0.4-0.9); yes, knees bent, back bent OR 1.2 (0.6-2.3); yes, knees straight, back bent OR 2.3 (1.1-4.7); yes, starting and ending the lift at the waist OR 2.0 (1.0-4.1)  Used free weights ≥10 times: no OR 1.0; yes OR 0.9 (0.6-1.4); yes, warmed up before workout <½ the time OR 0.5 (0.3-1.1); yes, warmed up before workout ≥½ the time OR 1.1 (0.7-1.7)  Used weightlifting equipment ≥10 times: no OR 1.0; yes OR 1.0 (0.7-1.5); yes, warmed up before workout <½ the time OR 0.5 (0.2-1.2); yes, warmed up before workout ≥½ the time OR 1.1 (0.7-1.7)  Confirmed cases  Non-occupational lifting ≥11.3kg inanimate objects: no OR 1.0; yes, ≥1/wk for 6mo OR 1.6 (1.0-2.5); yes, knees bent, back straight OR 0.7 (0.4-1.3); yes, knees bent, back bent OR 2.0 (0.8-4.9); yes, knees straight, back bent OR 4.0 (1.6-10.0); yes, starting and ending the lift at the waist OR 2.5 (1.1-6.0); yes, arms extended when lift started ≥½ the time OR 1.9 (1.0-3.5); yes, twisted while lifting ≥½ the time OR 1.9 (0.9-3.9) |
| Jørgensen, 1994 [18]  Denmark  Cohort | Occupationally active assistant nurses followed for LDH surgery in 1988 by record linkage to the Danish National Registry of Hospitalized Patients, and compared to all Danish females  Follow-up: 1y  N=1,681,152 (100%)  20-69y (NR)  100% | LDH surgery  Surgical  LDH surgery was ICD-8 surgical codes: 82073, ablatio prolapsus disci intervertebralis lumbalis; 82173, evacuatio disci intervertebralis lumbalis | Mod | Age, occupation | Phase II  Age: NR; incidence of LDH surgery increased with age up to the 45-49y age group in nurses, and the 50-54y age group in all Danish females  Occupation: all Danish females aged 30-69y RR 1.0; assistant nurses aged 30-69y RR 1.6 (1.2-2.2) |
| ^a^ Riihimäki, 1994 [19] ^b^ Pietri-Taleb, 1995 [51]  Finland  Cohort | Male machine operators, carpenters and office workers without prior history of sciatica in 1984, followed up in 1987  Follow-up: 3y  N=1,149 (0%);  25-49y (37)  83% | Sciatica  Clinical  Sciatica was LBP radiating to a leg in follow-up mailed questionnaire | Mod | ^a^ Age, occupation, seniority in occupation, education level, car driving, physical exercise, smoking, twisted or bent occupational postures, high pace of work, monotonous work, problems with workmates or superiors, draft, cold, vibration, history of back accidents, history of lumbago or other LBP  ^b^ Psychological distress and personality (subscales of the Middlesex Hospital Questionnaire and Maudsley Personality Inventory) | Phase II  ^a^ Occupation: office workers OR 1.0; machine operators OR 1.4 (1.0-1.9); carpenters OR 1.5 (1.1-2.1)  Physical exercise: maximum once per week OR 1.0; more than once per week OR 1.3 (1.0-1.6)  Smoking: non-smokers OR 1.0; smokers and ex-smokers OR 1.3 (1.0-1.7)  History of other low back pain: no OR 1.0; mild OR 2.7 (1.7-4.2); severe OR 4.5 (2.7-7.6)  ^b^ In blue-collar workers  Hysteria (quadratic term): OR per one unit increase, 1.3 (1.1-1.7) |
| Leclerc, 2003 [22]  France  Cohort | Male workers in the French national electricity and gas company without LBP during the past 12 months in 1992 were followed up in 1994  Follow-up: 2y  N=841 (0%)  40-50y at baseline (NR)  65% | Sciatica  Clinical  Sciatica was pain, discomfort or stiffness in the low back region at least 1 day in the previous 12 months, with radiating symptoms in the leg, in follow-up mailed questionnaire | Mod | Individual: age, living alone, height, BMI, exercise and sports, gardening, non-professional home construction activities, do-it-yourself, smoking  Health: history of low back pain, presence of neck pain, self-rated general health, score of psychological and psychosomatic well-being  Occupational: occupational category, score of job satisfaction and psychosocial aspects, prolonged sitting, prolonged standing, carrying loads, pulling or pushing heavy loads, bending forward and backward, trunk rotations, kneeling and squatting, driving | Phase II  Past history of LBP: no OR 1.0; yes OR 3.7 (1.9-7.4)  Height (cm): ≤180 OR 1.0; >180 OR 2.7 (1.2-6.4)  Driving for >2h: less than once per week OR 1.0; more than once per week OR 2.7 (1.2-6.4); daily OR 2.0 (0.9-4.4)  Self-rated health: good (score 1 or 2) OR 1.0; medium (score 3 or 4) OR 1.0 (0.5-2.0); bad (score 5-8) OR 2.9 (1.2-7.1)  Do-it-yourself activities: no OR 1.0; yes OR 2.0 (0.9-4.8) |
| ^a^ Jarvik, 2005 [23] ^b^ Suri, 2014 [24]  USA  Cohort | Outpatients without LBP or sciatica in the past 4 months from four clinics at the Veterans Affairs Puget Sound Health Care System, Seattle Division  Follow-up: 3y  N=148 (13%)  35-70y (median, 53)  89% | Clinical LDH and radicular symptoms  Clinical  ^a^ LDH was MRI-confirmed disc protrusion or extrusion, with pain frequency for low back or buttock pain rated as more than “some of the time” [and] sciatic leg pain; or numbness or tingling in the leg, foot, or groin; or weakness in leg or foot, rated as more than “none”  ^b^ Incident radicular symptoms defined as any self-reported sciatica, lower extremity numbness or tingling, or lower extremity weakness, at one or more time points over the 3-year follow-up | Mod | ^b^ Primary risk factors: incident MRI findings linked to radicular symptoms (central canal stenosis, disc extrusions, nerve root impingement) Other covariates: age, sex, race, BMI, current smoking status, depression, arthritis, and prior episodes of LBP and sciatica | ^b^ Phase II  MRI findings: No significant associations between incident MRI findings and the development of radicular symptoms.  Depression: Individuals with incident radicular symptoms were more likely to have depression at study inception (21.4% vs. 7.7%) than those without radicular symptoms.  Prior sciatica: More commonly reported among those with incident radicular symptoms than those without (12.9% vs. 3.8%) |
| ^a^ Sørensen, 2011 [25] ^b^ Jørgensen, 2013 [26]  Denmark  Cohort | Male workers in 1970 to 1971, at 14 private and public companies (railway, telephone, insurance, postal and firefighting) in Copenhagen, linked to Danish National Hospital Register (Copenhagen Male Study)  Follow-up: 6-33y  N=3,833 (0%)  40-59y (NR)  87% | Hospitalized LDH  Hospital  Hospitalization due to LDH identified in the National Hospital Register: ICD-8 code 725.11, from 1977 to 1994, and ICD-10 code M51.1, from 1994 to 2003 | Mod | ^a,b^ Occupational physical workload (ergonomic load to the back and strenuous work), height, weight, BMI, social class, leisure time physical activity, mental stress at work, mental stress in leisure time, use of sedatives, smoking, daily alcohol use  ^b^ Physical fitness (aerobic capacity) | Phase II  ^a,b^ Strenuous work (work resulting in sweating): seldom/never HR 1.0; Occasionally HR 2.4 (1.4-4.1); Often HR 3.9 (1.8-8.4)  Height (cm): ≤171 HR 1.0; 172-177 HR 2.2 (1.1-4.3); ≥178 HR 1.9 (0.9-3.9)  All other potential risk factors were not associated with incident LDH  ^b^ Physical fitness (VO_2_Max): low (<32) HR 1.0; high (≥33) HR 0.9 (0.5-1.5) |
| Wahlström, 2012 [27]  Sweden  Cohort | Male construction workers who participated in a national occupational health surveillance program from 1971 to 1992, linked to Swedish Hospital Discharge Register  Follow-up: 1-32y  N=263,529 (0%)  20-65y (NR)  ≥80% | Hospitalized LDH  Hospital  Primary diagnosis codes for LDH: 722.1 (“Displacement of thoracic or lumbar intervertebral disc without myelopathy,” ICD-9, 1987–1996) or M51.1 (“Lumbar and other intervertebral disc disorders with radiculopathy,” ICD-10, 1997-2003) | Mod | Age, height, weight, smoking, occupation | Phase II  Age (y): 20-29 RR 1.0; 30-39 RR 1.9 (1.6-2.2); 40-49 RR 1.8 (1.5-2.1); 50-59 RR 1.1 (0.9-1.3); 60-69 RR 0.9 (0.7-1.1)  Height (cm): 170-179 RR 1.0; 150-159 RR 0.9 (0.2-3.6); 160-169 RR 0.8 (0.7-1.0); 180-189 RR 1.3 (1.2-1.4); 190-199 RR 1.6 (1.3-1.9)  Weight (kg): 70-89 RR 1.0; 50-69 RR 0.8 (0.8-0.9); 90-99 RR 1.3 (1.1-1.5); 100-119 RR 1.4 (1.1-1.8); 120-149 RR 1.3 (0.6-3.0)  Smoking: never smoker RR 1.0; ex-smoker RR 1.1 (0.9-1.3); current smoker RR 1.3 (1.2-1.4); unknown smoking habits RR 1.3 (1.1-1.6)  Occupation: Incidence densities varied between the different groups of construction workers. Most groups of manual workers had an increased RR compared with white-collar workers and foremen. Incidence densities ranged from a low of 0.43 (0.38-0.50) per 1,000 person-years in white collar and foremen workers, to a high of 0.96 (0.60-1.54) per 1,000 person-years in refrigerator technicians |
| Zhang, 2016 [52]  China  Case-control | Cases: 396 patients with surgery for single-level LDH at Qilu Hospital from 2013-2014  Controls: 394 age and sex matched controls who had surgery for wounded lower limbs without evidence of LBP at the same hospital  Excluded controls and cases with diabetes, coronary heart disease, cerebrovascular disease, inflammatory arthritis and patients younger than 18 years  Excluded cases with lumbar spinal stenosis, spondylolisthesis, multiple intervertebral disc herniations, spinal tumor, history of spinal trauma and intervertebral space infection, previous surgery on the affected lumbar disc  Excluded controls with history of spinal disorders, trauma and low back pain, primary osteoarthritis of the operated or contralateral joint, previous surgery on the affected lower limbs  Follow-up: NA  N=790 (42%)  18-82y (42y)  NR | LDH surgery  Surgical  Cases: LBP with unilateral or bilateral lower limb radicular pain, special nerve root irritation signs (straight leg raising test, strengthen test or femoral stretch test depending on the level), neurologic deficit (muscle weakness, numbness, or lack of the corresponding reflex), CT or MRI with signs of a herniated disc  Controls: Lower limb fracture, a meniscal tear and cruciate ligament rupture diagnosed on the basis of clinical evaluation and imaging results | Mod | Fasting serum lipid levels of total cholesterol (TC), triglycerides (TG), low-density lipoprotein cholesterol (LDL-C) and high-density lipoprotein cholesterol (HDL-C), labour intensity, BMI | Phase II  High LDL-C serum levels: OR 1.5 (1.2-1.8)  High-TG serum levels: OR 3.0 (1.5-5.9)  Borderline high LDL-C: OR 1.6 (1.0-2.6) |
| Bjornsdottir, 2017 [53]  Iceland  Case-control | Cases: 4,748 patients with surgery for LDH from Landspitali National University Hospital Database from 1997- 2015  Controls: 282,590 controls without diagnosis of LDH from 1997-2015 and 6 months later  Excluded those with secondary osteoarthritis and post-trauma osteoarthritis  Follow-up: NA  N=287,338 (NR)  NR (45y among cases)  90% | LDH surgery  Surgical  No explicit case definition provided  Included all LDH surgeries | Mod | SNP rs6651255[C] | Phase II  rs6651255[C] OR 0.8 (0.8-0.9)  ≤40 years: rs6651255[C] OR 0.7 (0.7-0.8)  >40 years: rs6651255[C] OR 0.9 (0.8-0.9) |
| Chan, 2018 [29]  Taiwan  Cohort | Physician and non-physician healthcare professionals with LDH linked to the Taiwan National Health Research Database  Follow-up: 5y  N=115,488 (42%)  NR (47y)  100% | Clinical LDH  Clinical  ICD-9-CM diagnosis codes: 722.10, 722.52, 722.73, 722.93 | Mod | Occupational: Job as physicial or non-physician healthcare provider (e.g. pharmacists, medical technicians, audiologists, consultant experts, clinical experts, dieticians, social workers and language experts) | Phase II  General population OR 1.0, physician OR 1.1 (1.0-1.3), nonphysician HCP OR 1.2 (1.1-1.4)  Nonphysician HCP OR 1.0, physician OR 0.9 (0.8-1.1)  In men:  Nonphysician HCP OR 1.0, physician OR 0.9 (0.8-1.1)  In women:  Nonphysician HCP OR 1.0, physician OR 0.9 (0.7-1.2) |
| ^a^Dong, 2018 [54]  ^b^Zhu, 2018 [55]  China  Case-control | Cases: 380 Han Chinese patients with LDH recruited at the Xi’an Jiaotong Hospital Medical College Red Cross Hospital from 2015-2019  Controls: 692 unrelated healthy Han Chinese controls without any known disease and no history of cancer from the same region  Excluded those with trauma related LDH, complicated blood diseases, autoimmune diseases, tumors, trauma, rheumatoid arthritis, related lumbar spine disease containing lumbar spinal stenosis, spinal congenital dysplasia, intraspinal tumor or spondylolisthesis  Follow-up: NA  N=1,072 (42%)  >18y (49y)  NR | Clinical LDH and sciatica  Clinical  ^a^LDH was unilateral pain from the femoral or sciatic nerve to the corresponding dermatome of the nerve root >3 months duration, with positive MRI findings  ^b^Symptoms were described as lower back pain, pain in the inferior lumbar part of the spine and regional typical sciatica, difficulty in straight-leg raising and augmentation test, limited lumbar flexion range with confirmatory MRI | Mod | ^a^Aldehyde dehydrogenase 2 gene  Eight SNP’s in ALDH2 (rs886205, rs2238152, rs4648328, rs441, rs4646778, rs671, rs11066028, rs7296651)  ^b^Brain derived neurotrophic factor gene  Seven SNPs in BDNF (rs62656, rs11030104, rs10767664) and BDNFOS (rs988712, rs7481311, rs11030064, rs11030096) | ^a^Phase II  ALDH2(rs671): GG OR 1.0, GA OR 1.4 (1.0-1.8), AA OR 2.3 (0.9-5.4)  ALDH2(rs7296651): GG OR 1.0, CG OR 0.7 (0.5-1.0), CC OR 2.5 (0.9-7.3)  “A” of rs671: log-additive model OR 1.4 (1.1–1.8)  “C” of rs7296651: over-dominant model OR 0.7 (0.5–1.0)  Haplotype “GGCTCACG” of ALDH2 (rs886205, rs2238152, rs4648328, rs441, rs4646778, rs671, rs11066028, and rs7296651) OR 1.5 (1.1-1.9)  ^b^Phase II  ≤50y: BDNFOS SNP(rs11030064) TC codominant OR 0.7 (0.5-1.0), TC-TT dominant OR 0.7 (0.5-0.9), log-additive OR 0.7 (0.6-1.0)  <50y: BDNF SNP(rs6265) TC-TT dominant OR 1.5 (1.0-2.3), BDNF SNP(rs11030104) GA-GG dominant OR 1.5 (1.0-2.3), BDNF SNP(rs10767664) AT-TT dominant OR 1.6 (1.0-2.4), BDNF SNP (rs10767664) log-additive OR 1.3 (1.0-1.7)  Haplotype “GCC” (rs988712, rs7481311, rs11030064) of BDNFOS in age group >50y: OR 1.5 (1.0-2.1)  In women:  BDNFOS SNP (rs11030096): CC recessive OR 2.0 (1.1-3.7) |
| Fouquet, 2018 [30]  France  Cohort | ^a^Lumbar disc surgery (LDS) study:  Patients with LDH surgery from 2007-2008 who responded to a questionnaire  Follow-up: 2y  N=1,489 (NR)  20-59y (NR)  57%  ^b^Occupational disease - disc related sciatica (OD-DRS):  French workers compensated for disc related sciatica as an occupational disease living in the Pays de la Loire from 2009-2010  Follow-up: 2y  N=1,009 (NR)  20-59y (NR)  NR | ^a^LDH surgery  Surgical  Surgical codes for hospital discharge database (LHPH907 LFFA002 LFFA003 LFFC002 LFFA011 LFFA010 LHKA900 )  ^b^Clinical LDH  Clinical  ICD-10 code M511  Chronic LBP associated with lumbar disc herniation with radiculopathy caused by vibration or manual handling of loads | Mod | ^a,b^Occupation: Agriculture, hunting and forestry; fishing, aquaculture and related service; manufacturing; electricity, gas and water conditioning supply; construction; wholesale and retail trade, repair of motor vehicles and household goods; accommodation and food service activities; transportation and communication; financial activities; real estate, renting and business services; public administration and defense, compulsory social security; education; human health and social work activities; collective, social and personal activities, all non-missing sectors | ^a^Phase II  In men:  Construction sector: RR 0.7 (0.5-0.8)  Transportation and communication: RR 0.6 (0.5-0.8)  Public administration and defense, compulsory social security: RR 0.6 (0.5-0.7)  In women:  Accommodation and food service activities: RR 0.5 (0.3-0.8)  Transportation and communication: RR 0.6 (0.3-0.8)  Human health and social work activities: RR 0.6 (0.3-0.8)  ^b^Phase II:  In men:  Agriculture, hunting and forestry: RR 0.4 (0.3-0.5)  Manufacturing: RR 0.2 (0.2-0.3)  Construction: RR 0.6 (0.5-0.7)  In women:  Manufacturing: RR 0.2 (0.1-0.2)  Wholesale and retail trade, repair of motor vehicles and household goods: RR 0.1 (0.0-0.1)  Human health and social work activities: RR 0.1 (0.1-0.1) |
| Han, 2018 [31]  South Korea  Cohort | Nationwide cohort of Korean public officers (Police officer, firefighter, public educational officers) and national and regional government officers with claims data from 2002-2014 collected from the National Health Insurance Service  Follow-up: 13y  N=860,221 (36%)  NR (40y)  100% | Clinical LDH  Clinical  ICD-10 code M51 | Mod | Occupation: Police officers, firefighters, public educational officers and national and regional government officers | Phase II  Occupation: National and regional government officer HR 1.0, police officer HR 1.2 (1.2-1.2), firefighter HR 1.4 (1.4-1.5), public educational officer HR 1.0 |
| Jing, 2018 [56]  China  Case-control | Cases: 845 patients with LDH recruited from the Affiliated Hospital of Ya- n’an University and Yan’an People’s Hospital  Controls: 1,751 healthy controls without recent infections, history of tumors or lumbar sprain/chronic strain  Excluded those with mental illness, severe dysfunction of important organs (such as heart, lung, liver, or kidney), blood diseases, diabetes, autoimmune diseases, tumors, BMI ≤ 18.5 kg/m^2^ or ≥28 kg/m^2^  Follow-up: NA  N=2,596 (31%)  32-62y (48y)  NR | Clinical LDH  Clinical  LDH defined as physical signs and clinical symptoms (not further specified) with positive MRI imaging | Mod | Age, sex, BMI, family history, smoking, drinking, amateur exercise, leisure sports, bed type, spine load level, grade of disc degeneration, fourteen SNPs of matrix metalloproteinase-9 gene (MMP-9) | Phase II  MMP-9 SNP (rs3918242) T allele OR 1.2  MMP-9 SNP (rs17576) A allele OR 0.8  Positive family history: no OR 1.0, yes OR 1.4 (1.1-1.9)  Amateur exercise (Activities outside working hours): no OR 1.0, yes OR 0.6 (0.5-0.7)  Leisure sports (>20min of exercise ≥3 times/week): no OR 1.0, yes OR 0.5 (0.4-0.6)  Soft bed type: no OR 1.0, yes OR 1.2 (1.0-1.5)  High spine load level (Whole-body vibration, bending and twisting at work, lifting and heavy work): no OR 1.0, yes OR 3.4 (2.8-4.0) |
| Kim, 2018 [32]  Korea  Cohort | Nationwide cohort followed for LDH from 2004-2010 by record linkage to the National Health Insurance Service  Follow-up: 7y  N=18,786,256 (33%)  20-69y (NR)  100% | Clinical LDH  Clinical  The following diagnosis codes were used: M501, M510, MM11, M512 | Mod | Occupation (Regional subscribers (Own business such as restaurant or beauty salon with various forms of physical labor), company subscribers (70% field work with physical work, 30% office work), government subscribers (office work), private school subscribers (office work), medical care subscribers (Physical work such as daily construction site work)), age, sex | Phase II  The number of male patients with LDH was higher for those in their 30s, while there were more female patients with LDH ≥40y.  In men:  Number of patients with LDH highest in medical care subscribers, followed by company, government office and private school subscribers, and lowest in regional subscribers  In women:  Number of patients with LDH highest for medical subscribers, followed by company and regional subscribers and lowest at government office and private school subscribers |
| Knox, 2018 [33]  USA  Cohort | Military helicopter pilots and matched active-duty military service members with first time diagnosis of lumbar disc displacement linked to the Defense Medical Epidemiology Database followed from 2006-2015  Follow-up: 10y  N=NR (NR)  20-40y+ (NR)  100% | First-time diagnosis with lumbar disc displacement  Clinical  ICD-9 code 722.1 (lumbar disc displacement) | Mod | Age, sex, work branch affiliation | Phase II  Incidence rate compared to non-pilots: IRR 1.1 (0.7-1.8) for ages 20-24y, IRR 1.0 (0.9-1.2) for ages 25-29y, IRR 1.4 (1.2-1.6) for ages 30-34, IRR 1.3 (1.2-1.5) for ages 35-39, IRR 1.2 (1.1-1.4) for ages >40y  Age >30y versus <30y: IRR 2.9 (2.7-3.0) |
| Li, 2018 [57]  China  Case-control | Cases: 120 Chinese Han patients with LDH recruited from four hospitals in Tianjin from May 2015 till December 2015  Controls: 120 Han Chinese controls without LDH or other spine diseases recruited from the same hospitals  Excluded those with previous spinal surgery, patients with congenital spinal deformities, previous spine injury or spine tumor  Follow-up: NA  N=240 (30%)  >18y (55y)  NR | Clinical LDH  Clinical  LDH was defined on clinical presentation and positive MRI finding | Mod | Age, sex, BMI, vitamin D receptor (VDR) gene polymorphisms (Fok I (rs2228570), Apa I (rs7975232), Taq I (rs731236)) | Phase II  BMI >25 kg/m2 OR 0.3 (0.2-0.5)  Other findings:  VDR polymorphisms (Fok I, Apa I, Taq I) were not associated with increased LDH risk |
| ^a^Hu, 2019 [58] ^b^Ji 2019 [59] ^c^Liu 2020 [60] ^d^Wu 2020 [61] ^e^Yang 2020 [62] ^f^Hu 2022 [63] ^g^Han 2023 [64] ^h^Wu 2023 [65]  China  Case-control | Cases: 508 uninterruptedly recruited Han Chinese patients with LDH recruited from The Second Affiliated Hospital of Inner Mongolia Medical University in Hohhot from 2015-2017  Controls: 508 sex matched healthy Han Chinese controls from the annual health checkup center at the same hospital without sciatica, LBP, family history of LDH, infections, generalized musculoskeletal pain, diabetic polyneuropathy, cardiovascular disease, cancer, psychiatric disease, alcohol or drug abuse, acute or chronic inflammatory disease, hypertension, diabetes mellitus or poor DNA quality on blood sample  Excluded those with complicated blood diseases, autoimmune diseases, tumors, trauma, lumbar spinal stenosis, spinal congenital dysplasia, intraspinal tumor, spondylolisthesis , poliomyelitis and history of spinal surgery  Follow-up: NA  N=1,016 (42%)  NR (49y)  NR  ^f^N=1,007 (504 cases, 503 controls)  ^g^N=1,019 (509 cases, 510 controls)  ^h^N=1,004 (504 cases, 500 controls) | Clinical LDH and Sciatica  Clinical  LDH was defined by meeting the following criteria: MRI confirmed LDH, clinical symptoms for at least 4 weeks including limited lumbar flexion range, abnormal feeling of the lower limb skin, leg pain more severe than LBP, positive Lasègue test and 2 symptoms from muscle atrophy, decreased muscle strength, decreased sensation or decreased tendon reflex | Mod | ^a^Glypican-6 polymorphism  Six SNPs in GPC6 (rs4773724, rs1008993, rs9523981, rs7320969, rs59624626, rs995810)  ^b^Eyes shut homolog polymorphism  Five SNPs in EYS gene (rs62413038, rs1482456, rs9342097, rs9450607, rs7757884)  ^c^RAB40C gene polymorphism  Three SNPs in RAB40C gene (rs4984677, rs62030917 and rs2269556)  ^d^GSDMC gene polymorphism  Five SNPs in GSDMC gene (rs4527833, rs77681114, rs4285452, rs4733741 and rs4509280)  ^e^Storkhead box 1 gene polymorphism  Six SNPs in STOX1 (rs10998449, rs10762244, rs10998461, rs10998468, rs7903209, and rs4472827)  ^f^MIR31HG gene polymorphism  Seven SNPs in MIR31HG (rs1332184, rs72703442, rs2025327, rs55683539, rs2181559, rs10965059, and rs10965064)  ^g^ADAMTS6 and ADAMTS17 gene polymorphism  Five SNPs in ADAMTS6 and ADAMTS17 (rs17206779, rs2307121, rs2573625, rs4533267, rs4965593)  ^h^LINC-PINT gene polymorphism  Three SNPs in LINC-PINT (rs157916, rs16873842, rs7801029) | ^a-e^Phase II  ^a^Glypican-6 polymorphism  Allelic model analysis: GP6 SNP (rs4773724) G/T OR 0.8 (0.7–1.0), GP6 SNP (rs1008993) T/C, OR 1.3 (1.1–1.7)  GP6 haplotype TT (rs4773724, rs1008993) OR 1.3 (1.0 – 1.7)  GP6 haplotype GC (rs4773724, rs1008993) OR 0.8 (0.7 - 0.8)  ^b^Eyes shut homolog polymorphism  Allelic model analysis: EYS SNP (rs62413038) G/T OR 1.2 (1.0–1.4), EYS SNP( rs9450607) A/G OR 1.3 (1.1–1.5)  Genotypic model analysis: EYS SNP (rs62413038) log-additive OR 1.2 (1.0-1.4), EYS SNP (rs9450607) recessive OR 2.0 (1.2-3.1), EYS SNP (rs9450607) log-additive OR 1.3 (1.1-1.6)  EYS haplotype CGGA (rs1482456, rs9342097, rs9450607, rs7757884) OR 0.5 (0.3-0.9)  Age ≥49y: EYS SNP (rs9450607) OR 2.2 (1.1-4.4)  In men:  EYS SNP (rs62413038) OR 1.8 (1.1-2.8), EYS SNP (rs9342097) OR 1.9 (1.1-3.4), EYS SNP (rs9450607) OR 2.1 (1.1–3.9)  ^c^RAB40C gene polymorphism  Allelic model analysis: RAB40C SNP (rs62030917) G/A OR 1.2 (1.0-1.5), RAB40C SNP (rs2269556) G/A OR 1.2 (1.0-1.5)  Genotypic model analysis: RAB40C SNP (rs2269556) codominant OR 1.5 (1.0-2.2), log additive OR 1.2 (1.0-1.5)  Age <49y: RAB40C SNP (rs62030917) codominant GA OR 1.2 (0.8-1.7), codominant GG OR 3.0 (1.3-6.6), recessive GG OR 2.8 (1.3-6.2), log-additive OR 1.4 (1.1-1.9)  ^d^GSDMC gene polymorphism  Allelic model analysis: GSDMC SNP (rs77681114) A/G OR 0.8 (0.7–1.0)  Genotypic model analysis: GSDMC SNP (rs77681114) log-additive OR 0.8 (0.7-1.0)  GSDMC haplotype AG (rs77681114, rs4285452) OR 1.2 (1.0-1.5)  Age <49y: GSDMC SNP (rs4509280) codominant OR 1.7 (1.0–2.9), recessive OR 1.8 (1.1–2.9)  Age ≥49y: GSDMC SNP (rs77681114) alleles AG OR 0.7 (0.5–0.9), co-dominant AA OR 0.2 (0.1-0.7), dominant AG-AA OR 0.7 (0.5– 1.0), recessive AA OR (0.1–0.7), log-additive OR 0.7 (0.5–0.9)  In women:  GDSMC SNP (rs77681114) co-dominant OR 0.2 (0.1–0.9), recessive OR 0.3 (0.1–1.0)  ^e^Storkhead box 1 gene polymorphism  Allelic model analysis: STOX1 SNP (rs7903209) T/C OR 1.4 (1.0-1.8), STOX1 SNP (rs4472827) A/G OR 1.5 (1.1-2.0)  Genotypic analysis: STOX1 SNP (rs7903209) dominant CT-TT OR 1.4 (1.0-1.9), additive OR 1.4 (1.0-1.8). STOX1 SNP (rs4472827) genotype GA OR 1.5 (1.1-2.1), dominant GA-AA OR 1.5 (1.1-2.1), additive OR 1.4 (1.1-1.9)  STOX1 haplotype GT (rs10998461, rs10998468) OR 0.7 (0.5-0.9)  ^f^MIR31HG gene polymorphism  MIR31HG SNP(rs10965059): dominant OR 0.5 (0.3-0.6), log-additive OR 0.6 (0.5-0.8), codominant OR 0.4 (0.3-0.6)  >49y: MIR31HG SNP(rs10965059) allele T OR 0.6 (0.4-0.8), codominant C/T OR 0.3 (0.2-0.5), dominant C/T-T/T OR 0.4 (0.3-0.6), log additive OR 0.5 (0.4-0.8)  ≤49y: MIR31HG SNP(rs10965059) allele T OR 0.4 (0.3-0.6), co-dominant C/T OR 0.3 (0.2-0.4), dominant C/T-T/T OR 0.3 (0.2-0.5), log-additive OR 0.4 (0.3-0.6)  In men  MIR31HG SNP(rs10965059) allele T OR 0.6 (0.4-0.9), co-dominant C/T OR 0.5 (0.3-0.8), dominant C/T-T/T OR 0.5 (0.4-0.8), log-additive OR 0.6 (0.4-0.9)  ^g^ADAMTS6 and ADAMTS17 gene polymorphism  ADAMTS17 SNP(rs4533267): allele A OR 0.7 (0.6-0.9), co-dominant GA OR 0.7 (0.5-0.9), dominant OR 0.7 (0.5-0.9), log-additive OR 0.7 (0.6-0.9)  ≤48: ADAMTS17 SNP(rs4533267): co-dominant GA OR 0.6 (0.4-1.0), co-dominant AA OR 0.3 (0.1-0.9), dominant GA-AA OR 0.6 (0.4-0.9), log-additive OR 0.6 (0.4-0.8)  In women  ADAMTS17 SNP(rs4533267): co-dominant GA OR 0.4 (0.3-0.7), dominant GA-AA OR 0.5 (0.3-0.7), log-additive OR 0.5 (0.4-0.8)  ADAMTS6 SNP(rs2307121): co-dominant CT OR 1.6 (1.0-2.4), dominant CT-TT OR 1.6 (1.1-2.5), log-additive OR 1.5 (1.1-2.2)  ^h^LINC-PINT gene polymorphism  LINC-PINT SNP (rs157916) G/A OR 1.2 (1.0-1.5)  LINC PINT SNP(rs7801029) C/G OR 1.4 (1.1-1.7), codominant OR 2.3 (1.3-4.3), recessive OR 2.1 (1.2-3.9), additive OR 1.4 (1.1-1.7)  Haplotype GGG (rs157916, rs16873842, rs7801029) OR 1.4 (1.1-1.8)  In men  LINC-PINT SNP(rs157916): allele G OR 1.4 (1.1-1.8); codominant GG OR 1.9 (1.2-3.1), dominant GG-GA OR 1.6 (1.1-2.2), additive OR 1.4 (1.1-1.8)  LINC-PINT SNP(rs780129): allele G OR 1.5 (1.2-6.1), dominant GG-GC OR 1.6 (1.1-2.2), additive OR 1.6 (1.2-2.1) |
| Yang, 2019 [66]  China  Case-control | Cases: 380 Han Chinese patients recruited from Xi’an Honghui Hospital  Controls: 400 unrelated Han Chinese controls recruited from the same hospital with good health as confirmed by physical examination, no recent infections, no history of tumors, and no history of lumbar sprain and/or chronic strain  Excluded those with spinal and joint diseases such as trauma, spinal tumor, synovial cyst, inflammatory disease, scoliosis, osteoarthritis, spondylosis, and spondylolisthesis and patients with work time >8 hours per day or ≥60 pack years  Follow-up: NA  N=780 (41%)  NR (51y)  NR | Clinical LDH  Clinical  Cases were patients with a history of lumbar sprain and/or chronic strain, pain in the inferior lumbar part of the spine and regional sciatic nerve pain in the leg caused by bed rest, tenderness beside the lumbar spine that affects the leg or foot, limited lumbar flexion range, positive results in the straight-leg raising test and augmentation test (Bragard’s sign), or nerve injury symptoms (muscular atrophy, motor weakness, decreased sensation and hyporeflexia) in accordance with LDH findings on CR, MRI or CT | Mod | 15 SNPs in CHRNA3/CHRNA5 gene (rs667282, rs16969948, rs588765, rs6495306, rs17486278, rs680244, rs569207, rs692780, rs3743077, rs1317286, rs938682, rs12914385, rs2869546, rs3743075, rs8040868), drinking | Phase II  Smoking: no OR 1.0, yes OR 0.1 (0.1-0.2)  Drinking: no OR 1.0, yes OR 0.0 (0.0-0.1)  Haplotype ‘TACACCCG’ of CHRNA5 (rs667282, rs16969948, rs588765, rs6495306, rs17486278, rs680244, rs569207, rs692780): OR 0.8 (0.6-1.0), ≤50y OR 1.5 (1.0-2.1)  In men:  CHRNA3 SNP (rs8040868) TT vs CT OR 0.5 (0.3-0.8)  In women:  CHRNA3 SNP (rs8040868) TT vs CT+CC OR 0.5 (0.3-1.0) |
| ^a^Zhu, 2019 [67] ^b^Tai, 2020 [68]  China  Case-control | Cases: 498 unrelated Han Chinese patients with LDH from Shaanxi Province recruited at the Xi’an Jiaotong University Hospital Medical College Red College Red Cross Hospital from 2015-2017  Controls: 463 randomly recruited healthy individuals with no history of cancers or diseases  Excluded those with trauma-related LDH, blood diseases, autoimmune diseases, tumors, trauma, rheumatoid arthritis and related lumbar spine diseases such as lumbar spinal stenosis, congenital dysplasia of the spine, intraspinal tumor or spon- dylolisthesis  Follow-up: NA  N=961(41%)  >18y (50y)  NR | Clinical LDH and sciatica  Clinical  ^a^LDH was defined as low back pain, partial lumbar spine pain and local typical sciatica, differences in straight-leg elevation test and protuberance test with positive findings on CT or MRI  ^b^LDH was defined positive MRI for LDH and a history of unilateral pain from the femoral or sciatic nerve to the corresponding dermatome for more than 3 months | Mod | ^a^Inflammatory mediator genes  Five SNPs of IL1R1 gene (rs10490571, rs12712127, rs956730, rs3917225, rs3917318)  ^b^Inflammatory mediator genes  Five SNPs of Interleukin (IL)-1 gene (rs17042888, rs315919, rs928940, rs3181052, rs452204), age | ^a^Phase II  >50y: IL1R1 SNP(rs956730): TT recessive OR 3.4 (1.1-10.6)  In men:  IL1R1 SNP(rs956730): AG codominant OR 0.7 (0.5-0.9), AA codominant OR 2.4 (1.1-5.2), AA recessive OR 2.8 (1.3-6.1)  ^b^Phase II  IL1RN SNP (rs3181052) dominant AG-GG vs A OR 0.7 (0.6-1.0)  Age >50y: IL1RN SNP (rs17042888) allele AG OR 0.7 (0.5-1.0), dominant AG-AA vs GG OR 0.7 (0.5-1.0). IL1RN SNP (rs315919) recessive GG vs TT-GT 0.6 (0.4-1.0) |
| Luo, 2020 [69]  China  Case-control | Cases: 231 LDH patients recruited from the First Affiliated Hospital on Nanjing Medical University from 2012- 2018  Controls: 312 sex and age matched healthy controls without intervertebral disc diseases from the First Affiliated Hospital on Nanjing Medical University from 2012 till 2018  Excluded those with intervertebral disc diseases other than LDH  Follow-up: NA  543 (40%)  25-84y (46y)  NR | Clinical LDH  Clinical  LDH confirmed by MRI imaging | Mod | Matrix metalloproteinase (MMP)-3 gene polymorphism (SNP rs591058), prolonged sitting, bending/twisting, whole body vibration, lifting and heavy work load, smoking, drinking, back injury, age and sex | Phase II  Genotypic model analysis: MMP-3 SNP (rs591058) homozygote TT OR 1.9 (1.1-3.4), dominant TT+CT OR 1.5 (1.0-2.1)  Allelic model analysis: MMP-3 SNP(rs591058) T OR 1.4 (1.1-1.8)  Whole body vibration: MMP-3 SNP (rs591058) genotype TT OR 5.2 (2.0-13.7), genotype CT+TT OR 2.0 (1.2-3.5)  Bending/twisting: MMP-3 SNP (rs591058) genotype CT OR 1.9 (1.0-3.6), genotype TT OR 3.1 (1.1-8.7), genotype CT+TT 2.1 (1.2-3.8)  Lifting: MMP-3 SNP (rs591058) genotype TT OR 2.7 (1.1-6.7), genotype CT+TT OR 1.9 (1.1-3.3)  Smoking: no OR 1.0, yes OR 1.5 (1.1-2.1)  Back injury: no OR 1.0, yes OR 3.4 (2.2-5.3) |
| Fidan, 2022 [70]  Turkey  Case-control | Cases: 651 patients with LDH presenting to outpatient clinics between January 2021 and August 2021  Controls: 651 age and sex matched patients with LBP without LDH on MRI  Excluded those with a history of spondylolisthesis, spondylodiscitis, scoliosis, inflammatory spondylarthropathy, vertebral fracture, previous spinal surgery, primary or metastatic vertebral neoplasia and those radiographs on which the last thoracic vertebra could not be identified were excluded from the study  Follow-up: NA  N=1,302 (63%)  18-65y (43y)  71% | Clinical LDH  Clinical  LDH was defined as low back pain with positive finding of LDH on MRI taken within two weeks of indexed LBP symptoms | Mod | Age, sex, lumbosacral transitional vertebra | Phase II  Lumbosacral transitional vertebra: OR 2.3 (1.9-2.9)  Sacralization: OR 2.4 (2.0-3.1)  Lubarization: OR 0.9 (0.6-1.9) |
| Nyrhi, 2023 [36]  Finland  Cohort | Nationwide cohort of Finnish women from the Finnish Care Register for Health and the Finnish Medical Birth Register with a lumbar discectomy  Follow-up: 19y  N=13,912 (100%)  15-49y (NR)  100% | Surgically treated LDH  Surgical  Nordic-Medico-Statistical Committee classification codes: ABC01, ABC04, ABC07, ABC10, ABC13, ABC16, ABC17, ABC20, ABC23, ABC26 | Mod | Smoking | Phase II  Risk of LDH surgery during pregnancy: non-smoker OR 1.0, active smokers before pregnancy OR 2.0 (1.2-3.2) |
| Hurme, 1983 [15]  Finland  Cohort | AII lumbar disc herniation surgeries performed in South-West Finland from 1975-1979, based on surgical department registers of the Turku University Central Hospital area (mean area population during study period, 455,000)  Follow-up: 5y  N=1,011 surgeries (44%)  15-80y among cases (42 at surgery)  79% incident surgery | LDH surgery  Surgical  No explicit case definition provided  Included all operations performed for LDH, classified as first operations and reoperations | Mod | Sex, age, workload, home locality, seasonality | Phase I  Sex: NR; 56% of surgeries done on men  Age: NR; 90% of patients between 25 and 54 years of age  Workload: NR; neither heavy nor light work were more common among the incident surgery group than among the general population of Finland or South-West Finland |
| Noponen-Hietela, 2005 [71]  Finland  Case-control | Cases: 155 unrelated Finnish patients with sciatica from Oulu University Hospital area, 1997-1998  Controls: 179 unrelated University of Oulu employees and students (all Finnish), no information on possible MSK disorders  Excluded patients with chronic pain syndromes and neurological disorders  Follow-up: NA  334 (39% among cases, 69% among controls)  Cases: 19-78y (44); controls: 20-69y (39)  NR | Sciatica  Clinical  Disabling unilateral shooting band-like sciatic pain referring from the back to below the knee (dermatomes L4, L5, and S1) from 3 weeks to 6 months, nonresponsive to nonsteroidal anti-inflammatory agents, with clinical presentation concordant with MRI findings | Mod | Inflammatory mediator genes  Sequence variations (mutations) in 3 interleukin (IL) genes and 1 tumor necrosis factor (TNF) gene: IL1A, IL1B, IL6, TNFA  16 SNPs in 10 candidate cytokine genes: IL1A, IL1B, IL1 receptor antagonist (IL1RN), IL2, IL4, IL4R, IL6, IL10, TNFA, and interferon γ (IFNG) | Phase I  Genotypes of the IL6 SNP, T15A in exon 5, (AA and AT v TT) OR 4.4 (1.2-15.7)  Genotypes (haplotype pairs) of IL6, (GGGA/GGGA or GGGA/other v other/other) OR 5.4 (1.5-19.2)  Other findings  No mutations identified in the IL1A, IL1B, IL6 or TNFA genes were associated with sciatica |
| Mio, 2007 [72]  Japan  Case-control | Cases: 823 patients of Japanese origin with LDH from hospitals in the Toyama, Tokyo, and Kyoto areas  Controls: 841 hospital patients of Japanese origin who received medical examinations  Excluded patients with spinal canal stenosis, spondylolisthesis, spondylosis, synovial cysts, spinal tumor, and trauma; and those with occupational and/or habitual risk factors, such as heavy manual laborers, occupational drivers, and heavy smokers  Follow-up: NA  1,664 (41% among cases, 63% among controls  Cases:11-83y (36); controls: 13-87y (61)  NR | Clinical LDH  Clinical  LDH was unilateral pain radiating from the back along the femoral or sciatic nerve to the corresponding dermatome of the nerve root of >3 months duration, with positive MRI findings | Mod | Cartilage collagen genes  SNPs in 3 type XI collagen genes: COL11A1, COL11A2, COL2A1 | Phase I  COL11A1 (rs1676486) SNP, c.4603C→T in exon 62, OR 1.4 (1.2-1.7) |
| Virtanen, 2007 [73]  Finland & China  Case-control | Finland  Cases: 243 unrelated Finnish patients with sciatica from Oulu University Hospital area  Controls: 259 unrelated Finnish persons from the same area  502 (45%)  NR (NR)  NR  China  Irrelevant outcome | Sciatica  Clinical  Disabling unilateral shooting band-like sciatic pain referring from the back to below the knee (dermatomes L4, L5, and S1) from 3 weeks to 6 months, nonresponsive to nonsteroidal anti-inflammatory agents, with clinical presentation concordant with MRI findings | Mod | Cartilage intermediate layer protein (CILP) gene  Functional SNP (rs2073711), +1184T→C, in exon 8 of the CILP gene  Other SNPs in the CILP gene | Phase I  CILP SNP (rs2073711), +1184T→C in exon 8, OR 1.4 (1.0-1.9) |
| Hirose, 2008 [74]  Japan  Case-control | Cases: 847 patients of Japanese origin with LDH from 19 hospitals between 2001-2007  Controls: 896 Japanese persons from the same catchment area  Excluded patients with synovial cyst, spinal tumor, spondylosis, spondylolisthesis, trauma, and inflammatory disease  Follow-up: NA  1,743 (38%)  Cases: NR (39); controls: NR (62)  NR | Clinical LDH  Clinical  LDH defined by meeting 3 criteria: i) a history of unilateral pain radiating from the back along the femoral or sciatic nerve to the corresponding dermatome of the nerve root for >3 months; ii) diagnosis of LDH by MRI; and iii) treatment and monitoring for >1 year by an orthopaedic surgeon | Mod | Intervertebral disc extracellular matrix protein genes  SNPs in the 2 thrombospondin genes: THBS1, THBS2  SNPs in 2 matrix metalloproteinases: MMP2, MMP9 | Phase I  THBS2 SNP (rs9406328), IVS10-8C→T, OR 1.4 (1.2-1.6)  MMP9 SNP (rs17576), Q279R, OR 1.3 (1.1-1.5)  Genotypes for combined effect of THBS2 (TT/TC/CC) and MMP9 (GG/GA/AA): CC and AA, OR 1.0; TC and GA, OR 1.8 (0.9-3.3); TT and GG, OR 3.0 (1.6-5.8) |
| Karasugi, 2009 [75]  Japan & Finland  Case-control | Japan  Cases: 862 patients of Japanese origin with LDH from 20 hospitals between 2001-2007  Controls: 896 Japanese persons from the same catchment area  Excluded patients with synovial cyst, spinal tumor, spondylosis, spondylolisthesis, trauma, and inflammatory disease  1,758 (NR)  Cases: NR (39); controls: NR (62)  38%  Finland  Cases: 257 unrelated Finnish patients with sciatica from the Oulu University Hospital catchment area  Controls: 249 unrelated Finnish persons from the same area  Follow-up: NA  506 (NR)  NR (NR)  NR | Japan  Clinical LDH  Clinical  LDH was defined by meeting 3 criteria: i) a history of unilateral pain radiating from the back along the femoral or sciatic nerve to the corresponding dermatome of the nerve root for >3 months; ii) diagnosis of LDH by MRI; and iii) treatment and monitoring for >1 year by an orthopaedic surgeon  Finland  Sciatica  Clinical  Disabling unilateral shooting band-like sciatic pain referring from the back to below the knee (dermatomes L4, L5, and S1) from 3 weeks to 6 months, nonresponsive to nonsteroidal anti-inflammatory agents, with clinical presentation concordant with MRI findings | Mod | Human sickle tail (SKT) gene  Japan: 68 tag SNPs of the SKT gene  Finland: SKT SNP (rs16924573) only | Phase I  LDH in Japan  SKT SNP (rs16924573), OR 1.3 (1.1-1.6)  SKT SNP (rs2285592), OR 1.3 (1.1-1.5)  SKT SNP (rs17469499), OR 1.2 (1.0-1.5)  Sciatica in Finland  SKT SNP (rs16924573), OR 2.8 (1.1-7.2) |
| Cong, 2010 [76]  China  Case-control | Cases: 70 male patients of Chinese Han origin with LDH, from the First Affiliated Hospital of China Medical University  Controls: 14 male spinal trauma patients who underwent surgery at the same hospital and 113 male healthy blood donors without LDH symptoms  Excluded persons with occupational and lifestyle risk factors for LDH, such as heavy manual labour, occupational driving and heavy smoking  Follow-up: NA  197 (0%)  Cases: 14-41y (33); controls: 20-49y (38)  NR | Clinical LDH  Clinical  Radicular pain with signs of positive nerve root tension or neurologic deficit, a confirmatory imaging study (MRI and/or CT scan) indicating LDH corresponding to the symptoms and presence of symptoms for ≥6 weeks | Mod | Aggrecan gene  Aggrecan gene VNTR polymorphism  Expression of aggrecan | Phase I  Aggrecan VNTR polymorphism, A25 allele OR 2.1 (1.1-4.0); A21 allele OR 11.8 (2.1-65.5); A29 allele OR 0.2 (0.1-0.8) |
| Mu, 2014 [77]  China  Case-control | Cases: 231 Han Chinese patients with LDH recruited from the 150^th^ Hospital and Xijing Hospital from 2011-2012  Controls: 370 Han Chinese controls who never experienced symptoms suggesting LDH  Excluded those with osteoarthritis, previous fractures of the spine, lumbar spinal stenosis, malignancies involving the spine and poliomyelitis  Follow-up: NA  N=601 (45%)  NR (48y)  NR | Clinical LDH and sciatica  Clinical  LDH was defined as unilateral pain radiating from the back along the femoral or sciatic nerve to the corresponding dermatome of the nerve root for more than 1 month with positive MRI findings | Mod | Growth differentiation factor 5 gene  One SNP in GDF5 (rs143383) | Phase I  GDF5 SNP(rs143383): TT OR 1.9 (1.4-2.6), CC OR 0.3 (0.1-0.7), T vs C allele OR 1.8 (1.4-2.4)  In men:  GDF5 SNP(rs143383): TT OR 2.0 (1.3-3.2), CC OR 0.3 (0.1-0.8), T vs C allele OR 2.0 (1.4-2.8)  In women:  GDF5 SNP(rs143383): TT OR 1.7 (1.0-2.8), T vs C allele OR 1.7 (1.1-2.5) |
| Huang, 2017 [78]  China  Case-control | Cases: 267 Han Chinese patients with LDH from 2014-2015 from the Orthopedic Department of the Affiliated Hospital of Jining Medical College and The People’s Hospital of Zhangqiu  Controls: 300 Han Chinese individuals with similar age and geographical location without lumbocrural pain, family history of lumbocrural pain and osteoarthritis  Excluded those with complicated liver, kidney, cardiovascular, and cerebrovascular diseases, respiratory diseases, psychosis, complicated blood diseases, diabetes, autoimmune diseases, tumors, BMI <18.5 kg/m2 or >28.0 kg/m2, smoking index >300  Follow-up: NA  N=567 (39%)  18-60y (44y)  NR | Clinical LDH  Clinical  LDH was defined by clinical symptoms, physical signs and positive MRI findings | Mod | Inflammatory mediator genes  IL-6 (IL-6 572C/G and 174G/C SNPs) and IL-10 (IL-10 592A/C and 1082G/A SNPs) | Phase I  IL-6-572 C/G:  Genotype frequency: CC OR 1.0, CG OR 1.6 (1.1-2.3), GG OR 4.5 (1.6-15.3), GG+GC OR 1.7 (1.2-2.4)  Allele frequency: C OR 1.0, G OR 1.6 (1.2-1.6)  IL-10-1082 A/G:  Genotype frequency: AG OR 1.0, AA OR 2.7 (1.4-6.3)  Allele frequency: G OR 1.0, A OR 2.4 (1.4-5.9) |
| Jiang, 2017 [79]  China  Case-Control | Cases: 156 Han Chinese patients with lumbar spine pathologies recruited from Spine Surgery at the First Affiliated Hospital of Guangxi Medical University between 2012-2016  Controls: 400 Han Chinese controls from the Physical Examination Centre at the First Affiliated Hospital of Guangxi Medical University without clinical or radiographic signs of lumbar disc disease or LBP  Excluded those with presence of intraspinal tumors, trauma, inflammatory diseases and rheumatoid arthritis.  Follow-up: NA  N=556 (45%)  NR (45y)  NR | Clinical LDH  Clinical  Lumbar disc disease was defined by MRI-confirmation and history of low back pain or/and leg pain for longer than 3 months | Mod | Physical work load, age, sex, BMI, smoking habits, exposure to vibration, presence of other orthopedic conditions, six SNPs (rs1337185, rs5275, rs5277, rs7575934, rs3213718, rs162509) | Phase I  COL11A1 SNP (rs1337185) C allele OR 1.8 (1.2–2.7)  ADAMTS5 SNP (rs162509) G allele OR 1.6 (1.2–2.1) |
| Ghandhari, 2018 [80]  Iran  Case-control | Cases: 129 patients with LDH and signs of radiculopathy recruited in 2013  Controls: 61 controls without LBP or radiculopathy  Excluded those with multi-level disc herniation, degenerative changes, concomitant spinal deformities such as kyphoscoliosis, spina bifida, spinal infection, spinal stenosis, spondylolisthesis, history of spinal surgery and tumor or metastatic disease  Follow-up: NA  N=190 (37%)  NR (41y)  17% | Clinical LDH  Clinical  LDH was defined by clinical signs and symptoms of radiculopathy (radicular pain and/or paresthesia, positive straight leg raising, and one of the following: dermatomal hypesthesia, depressed deep tendon reflexes, or weakness in the region of the affected nerve root) with positive findings of LDH on MRI | Mod | Facet tropsim | Phase I  Incidence of facet tropism with LDH at level L4/5: cases 48.5%, controls 26.2% |
| Wang, 2018 [81]  China  Case-control | Cases: 134 patients with LDH treated in Huashan Hospital of Fudan University from 2010-2015  Controls: 100 healthy controls without lumbar disc degeneration or bony structural abnormal or significant degeneration in any lumbar disc matched for sex, age, BMI and occupation  Excluded those with history of spine surgery, scoliosis, lumbar fractures, tuberculosis, tumor and infections  N=234 (35%)  18-39y (30y)  NR | Hospitalized LDH  Hospital  LDH confirmed by MRI imaging | Mod | Asymmetry of superior and inferior endplates | Phase I  Asymmetry of endplates L4/5: degenerated LDH discs group 47%, non-degenerated LDH disc group 21%, controls 7%  Asymmetry of endplates L5/S1: degenerated LDH discs group 73%, non-degenerated LDH disc group 55%, controls 38% |
| Withanage, 2018 [82]  Sri Lanka  Case-control | Cases: 51 patients recruited from a hospital in the district of Colombo with LBP and imaging confirmed LDH  Controls: 68 controls recruited from several districts of Sri Lanka without LBP one month prior and without prior LDH surgery  Excluded those with bone disorders such as osteoarthritis, osteoporosis and supplementation on vitamin D, pregnancy and malignancies  Follow-up: NA  N=119 (52%)  18-74y (42y)  NR | Clinical LDH  Clinical  Low back pain with MRI confirmed LDH diagnosis | Mod | Multivariable analysis for Vitamin D receptor (VDR) polymorphism, 25(OH)D level, calcium levels | Phase I  Exposure to sunlight: limited OR 1.0, abundant OR 2.8 (0.5-22.5)  Other findings:  No statistically significant association was observed between VDR polymorphisms was observed |
| Zhou, 2018 [83]  China  Case-control | Cases: 53 patients with LDH recruited from the Tianjin First Center Hospital  Control: 129 controls with neither LDH, LBP, history of low back pain, sciatica, claudication nor previous problems involving the lower limbs from the same hospital  Excluded those with scoliosis, spondylolisthesis, transitional vertebra, vertebral fracture, previous surgery, previous trauma/spinal infection, and facet arthropathy  Follow-up: NA  N=182 (42%)  18-35y (28y)  NR | Clinical LDH  Clinical  LDH was defined as by clinical symptoms such as radicular radiating leg pain, radicular radiating leg paresthesia, positive straight leg raising test and or one neurological sign (dermatomal hypoesthesia, depressed deep tendon reflexes or muscle weakness in the distribution of the affected nerve root) confirmed by CT and MRI | Mod | Facet tropism and orientation | Phase I  LDH was significantly associated with more coronal facet joint orientation at L1/2, L2/3 and L3/4 |
| Abbreviations: BMI, body mass index; cig, cigarettes; cm, centimeter; CT, computerized tomography; d, day; h, hour; HR, hazard ratio; ICD, international classification of diseases; IRR, incidence rate ratio; kg, kilogram; LBP, low back pain; LDH, lumbar disc herniation; m, meter; mo, month; MRI, magnetic resonance imaging; MSK, musculoskeletal; N, study size; NA, not applicable; Nh, Newton-hours; NR, not reported; OR, odds ratio; ROB, risk of bias; RR, relative risk; SMR, standardized morbidity ratio; SNP, single nucleotide polymorphism; v, versus; VNTR, variable number of tandem repeats; wk, week; y, year | | | | | |

**Appendix 6.** Inadmissible studies examining the incidence of LDH with radiculopathy (n = 7)

| First author, Year published  Country  Study design | Study population and setting  Follow-up  N (% female)  Age range (mean)  Participation % | Study outcome  Case definition type  Case definition | ROB | Incidence estimates (95% CI) |
| --- | --- | --- | --- | --- |
| Bongers, 1988 [84]  Netherlands  Cohort | All male crane workers and floor workers employed in the same departments at a steel company on January 1, 1975 and those hired up to the end of 1979, were followed up for incident disability pensions due to LDH up until December 31, 1984  Follow-up: 10y  N=1,405 (0%)  <25-60y+ (NR)  71% | Disability pension due to LDH  Clinical  Disability pension due to displacement of intervertebral disc defined as ICD-9 diagnosis code 722.2, assigned by social insurance physician after evaluation of the social insurance medical records | High | Incidence density of LDH disability pension among crane operators, 2.9 (1.6-4.9) per 1,000 person-years; among floor workers, 1.3 (0.5-2.9) per 1,000 person-years  Raw data: 13/4,454.2; 5/3,805.8 |
| Netterstrøm, 1989 [85]  Denmark  Cohort | All full-time male bus drivers employed on April 1, 1978 by 3 urban bus companies followed up from 1978-1984 for hospitalized LDH in the Danish National Patient Register  Follow-up: 7y  N=2,465 (0%)  20-69y (NR)  100% | Hospitalized LDH  Hospital  ICD discharge diagnosis codes for LDH (725.10-725.11) | High | Incidence density of hospitalized LDH: 3.6 (2.8-4.6) per 1,000 person-years  7-year cumulative incidence of hospitalized LDH: 14.6 (10.6-20.2) per 1,000 persons  Average annual incidence: 2.1 (1.5-2.9) per 1,000 persons  Raw data: 62/17,122; 36/2,465 |
| Rivinoja, 2011 [86]  Finnland  Cohort | 1966 Northern Finland Birth Cohort followed from 1981-2008 with linkage to the Finnish Hospital Discharge Register  Follow-up: 28y  N=9,016 (50%)  NR (NR)  97% | Hospitalized sciatica and LDH surgery  Hospital and surgical  Hospitalization due to sciatica was defined by ICD-8 codes 35399 and 72510, ICD-9 codes 7227C and 7229X and ICD-10 code M511.  Hospitalization due to LDH surgery was defined by the surgical codes 9211, ABC07, ABC16, ABC26 and ABC36 | High | 28-year cumulative incidence LDH surgery: 22.5 (19.7-25.8) per 1,000 persons  Average annual incidence of LDH surgery: 0.8 (0.7-0.9) per 1,000 persons  Raw data: 203/9,016 |
| Roquelaure, 2011 [87]  France  Cohort | All residents of the Loire-Atlantique region discharged in 2002-2003 following LDH surgery  Follow-up: 2y  N=272 (56%)  20-59y (42y)  49-60% | Surgical LDH  Surgical  ICD-10th codes M51.1 (lumbar and other disc disorders with radiculopathy), M51.2 (other specified intervertebral disc displacement), M51.8 (other specified intervertebral disc disorders) and M51.9 (intervertebral disc disorders, unspecified). Hospital admissions were eligible only if the surgical procedure included conventional lumbar discectomy or microendoscopic lumbar discectomy. | High | NR |
| Chung, 2013 [88]  Taiwan  Cohort | Nurses and referent participants from the Taiwanese National Health Insurance Research Database between 2004-2010  Follow-up: 7y  N= 3,914, nurses; 11,744 referents (99%)  NR (34y for women, 31y for men)  NR | Hospital LDH  Hospital  LDH defined as ICD-9-CM code 722.10 | High | 1-year cumulative incidence of LDH in nurses: 14.5 (3.3-61.6) per 1,000 persons  1-year cumulative incidence of LDH in general population referent group: 6.4 (0.8-48.5) per 1,000 persons  Raw data: 1.45/100 in nurses, 0.64/100 in referent |
| Makovicka, 2019 [89]  USA  Cohort | Injuries in collegiate football players utilizing the NCAA Injury Surveillance Program (ISP) database  Follow-up: 5y  NR (NR)  NR (NR)  NR | Clinical LDH and sciatica  Clinical  No further information on case definition | High | Radiculopathy: Injury rate 0.2/10,000 AE’s (athlete-exposure, defined as one NCAA-sanctioned practice or competition)  LDH: Injury rate 0.2/10,000 AE’s  Raw data: 108 radiculopathies, 540 LDH, 7076 low back injuries, denominators: NR |
| Bailey, 2022 [90]  USA  Cohort | Twelve NASA astronauts spending 6 months in space from 2011-2018  Follow-up: 8m-2.5y  N=12 (17%)  NR (51y)  NR | Clinical LDH  Clinical  Clinical signs of LDH and confirmatory MRI scan | High | Cumulative incidence: 500 (253.8-746.2) per 1,000 persons  Raw data: 6/12 |
| Abbreviations: CT, computerized tomography; HNP, herniated nucleus pulposus; ICD, international classification of diseases; LBP, low back pain; LDH, lumbar disc herniation; Mod, moderate; MRI, magnetic resonance imaging; N, study size; NR, not reported; ROB, risk of bias; y, years | | | | |

**Appendix 7.** Inadmissible studies examining risk factors for LDH with radiculopathy (n = 27)

| First author, Year of publication  Country  Study design | Participants and setting  Follow-up  N (% female)  Age range (mean)  Participation % | Study outcome  Case definition type  Case definition | ROB | Risk factors considered | Phase of evidence  Risk estimates (95% CI) |
| --- | --- | --- | --- | --- | --- |
| Hrubec, 1975 [91]  NR  Case-control | Cases: 1,132 first admission records to Army hospitals for LDH in 1944-1945  Controls: 1,095 records of Army National Service Life Insurance policyholders individually matched on age and period of military service  Follow-up: NA  1,095 case-control pairs (0%)  18-56y (NR)  97% | Hospitalized LDH  Hospital  No explicit case definition provided  Records of first admission for LDH identified using punch cards from the Office of the Surgeon General | High | Height, weight, posture, body frame, place of birth, place of residence, education, occupation, marital status, physical defects, military occupation specialty assignments, overseas service, blood type, religion, rank, combat credit (medals or decorations) | Phase II  All statistically significant associations:  Height (cm): 152-164 OR 0.4; 165-181 OR 0.9; 182-203 OR 2.6  Weight (kg): 45-58 OR 0.5; 59-81 OR 1.0; 82-136 OR 1.6  Craftsman, foreman or kindred occupation: OR 1.5  Clerical or kindred occupation: OR 0.6  Married or re-married at induction: OR 1.4  Place of birth <2500 population or rural: OR 1.6  Rural free delivery address: OR 1.6  Good posture at induction: OR 1.3  Defects at induction relevant to HNP affecting back or legs: OR 3.5  Heavy frame at induction: OR 1.5  Military occupation specialty, ground combat: OR 1.6  Combat credit, 2+ battle stars: OR 0.7  Rank, staff sergeant or sergeant: OR 1.3 |
| Bongers, 1988 [84]  Netherlands  Cohort | All male crane workers and floor workers employed in the same departments at a steel company on January 1, 1975 and those hired up to 1979, followed up for incident disability pensions due to LDH until December 1984  Follow-up: 10y  N=1,405 (0%)  <25-60y+ (NR)  71% | Disability pension due to LDH  Clinical  Disability pension due to displacement of intervertebral disc defined as ICD-9 diagnosis code 722.2, assigned by social insurance physician after evaluation of the social insurance medical records | High | Whole-body vibration | Phase II  Disability pension due to LDH in crane operators exposed to whole-body vibration v floor workers, IDR 2.5 (90% CI, 1.2-12.5) |
| Chibnall, 2006 [92]  USA  Cohort | African American and non-Hispanic white workers’ compensation claimants who filed low back injury claims in St. Louis City or Kansas City, Missouri, and whose claims were settled between January 1, 2001, and June 1, 2002  Follow-up: NA  2,934 (38%)  18-55y (NR)  50% | Clinical and surgical LDH  Clinical and surgical  No explicit case definition provided  From a series of medical diagnosis questions, participants were categorized into 2 diagnosis groups: LDH v low back sprain/strain/pain (regional backache)  For claimants with a LDH diagnosis, surgery was classified as “no” v “yes” | High | Sex, age, race, socioeconomic status, presence of lower extremity pain, lumbar degeneration, legal representation, legal representation due to dissatisfaction with medical treatment, current work status | Phase II  LDH diagnosis (v regional backache diagnosis)  Sex: women OR 1.0; men OR 1.7 (1.4-2.2)  Age (y): OR per one unit increase, 1.2 (1.1-1.3)  Race: white OR 1.0; African American OR 0.6 (0.4-0.7)  Lower extremity pain: no OR 1.0; yes OR 3.3 (2.3-4.9)  Lumbar degeneration: no OR 1.0; yes OR 2.1 (1.6-2.6)  Legal representation: no OR 1.0; yes OR 1.0 (0.7-1.4)  Legal representation due to dissatisfaction with medical treatment: no OR 1.0; yes OR 0.6 (0.5-0.8)  Surgery among those with LDH diagnosis  Race: white OR 1.0; African American OR 0.3 (0.2-0.5)  Socioeconomic status: OR per one unit increase, 1.1 (1.0-1.4)  Lower extremity pain: no OR 1.0; yes OR 2.9 (1.2-6.4)  Legal representation: no OR 1.0; yes OR 1.4 (0.9-2.3)  Legal representation due to dissatisfaction with medical treatment: no OR 1.0; yes OR 0.6 (0.4-0.8) |
| Zhang, 2009 [93]  China  Case-control | Cases: 2,010 patients with LDH in orthopaedic departments at 3 hospitals between 2005-2007  Controls: 2,170 randomly selected patients (in-patients or medical exam) without current back/sciatic pain, or back/sciatic pain for >1 month ever, matched on race, sex, age, and living area  Excluded patients with lumbar spinal stenosis, spinal congenital dysplasia, intraspinal tumor, spinal instability from trauma, scoliosis, and spondylolisthesis  Follow-up: NA  4,180 (40%)  <30-50y+ (46y)  NR | Clinical LDH  Clinical  No explicit case definition provided  LDH diagnosis evaluated by ≥2 orthopaedic experts in terms of patient’s symptoms, signs and imaging examination (MRI and/or CT scan) among patients presenting with back-leg pain and typical sciatica | High | Sex, age, height, weight, smoking, drinking, family history of lumbar disc herniation, bed characteristics (hard, soft), educational background (primary school, secondary school, high school, university), physical exercise, occupational lumbar load (quite light, light, medium, heavy), occupational character (non-manual, half non-manual/half manual, manual), psychosocial factors at work (monotonous, boring, time urgency, too much responsibility, life pressure, hard-working) | Phase II  Among all persons  Family history OR 3.6 (1.9-6.5)  Lumbar load OR 2.1 (1.7-2.6)  Hard-working OR 1.8 (1.1-2.5)  Educational background OR 0.8 (0.6-1.0)  Physical exercise OR 0.4 (0.2-0.8)  Bed characteristics (hard v soft) OR 0.4 (0.2-0.6)  Other findings  Model omitted sex, age, BMI, occupational character, drinking, smoking, and vocational activities |
| Rivinoja, 2011 [86]  Finnland  Cohort | 1966 Northern Finland Birth Cohort followed from 1981-2008 with linkage to the Finnish Hospital Discharge Register  Follow-up: 28y  N=9,016 (50%)  NR (NR)  97% | Hospitalized Sciatica and LDH surgery  Hospital and surgical  Hospitalization due to sciatica was defined by ICD-8 codes 35399 and 72510, ICD-9 codes 7227C and 7229X and ICD-10 code M511.  Hospitalization due to LDH surgery was defined by the surgical codes 9211, ABC07, ABC16, ABC26 and ABC36 | High | Sports, smoking, BMI | Phase II  LDH surgery: female OR 1.0, male OR 2.6 (1.7-3.6)  In men:  First time hospitalization due to sciatica for non-surgical treatment: smoking HR 2.0 (1.1-3.6)  In women:  First time hospitalization due to sciatica: smoking HR 1.8 (0.8-3.9) |
| Roquelaure, 2011 [87]  France  Cohort | All residents of the Loire-Atlantique region discharged in 2002-2003 following LDH surgery  Follow-up: 2y  N=272 (56%)  20-59y (42y)  49-60% | Surgical LDH  Surgical  Lumbar or sacral discectomy for disc-related radiculopathy: admissions for ICD-10 codes M511 (lumbar and other disc disorders with radiculopathy), M512 (other specified intervertebral disc displacement), M518 (other specified intervertebral disc disorders) and M519 (intervertebral disc disorders, unspecified). | High | Sex, occupation, industry sector | Phase II |
| Chung, 2013 [88]  Taiwan  Cohort | Nurses and referent participants from the Taiwanese National Health Insurance Research Database between 2004-2010  Follow-up: 7y  N=3,914 nurses (99%); 11,744 referents (99%)  NR (34y for women, 31y for men)  NR | Hospitalized LDH  Hospital  LDH defined as ICD-9-CM code 722.10. | High | Age and nursing occupation | Phase II |
| Zhang, 2013 [94]  China  Case-control | Cases: Orthopaedic and spine surgery hospital patients with LDH, 2005-2007  Controls: Hospital inpatients or participants of medical exams matched for race, sex, age, living location  Follow-up: NA  N=268 (38%)  Cases: NR (46y)  Controls: NR (47y)  97% | Hospitalized LDH  Hospital  Hospitalization for reasons of back leg pain, CT/MRI diagnosed LDH and typical sciatica | High | Apoptosis-related genes (FAS, FASL, and caspase-9), smoking, alcohol consumption, leisure activities, amateur sports, and occupational lumbar load | Phase II |
| Chiang, 2014 [95]  China  Case-control | Cases: Hospital patients with LDH  Controls: Hospital patients without LDH from a medical or surgery department  Follow-up: NA  N=822 (31%)  Cases: NR (36y)  Controls: NR (42y)  NR | Hospitalized LDH  Hospital  LDH was the primary discharge diagnosis; patients were symptomatic and admitted for conservative or surgical treatment; and CT/MRI imaging evidence of LDH between L1 and S1 | High | Age, sex, height, weight, smoking | Phase II |
| Lee, 2015 [96]  South Korea  Case-control | Cases: Hospital patients with LDH treated conservatively, with selective nerve root block or surgery, from 2010-2011  Controls: Patients without LDH from an outpatient clinic matched for age, race and BMI  Follow-up: NA  N=565 (0%)  20-30y (23y)  NR | Clinical, hospital or surgical LDH  Clinical, hospital or surgical  Conservative treatment or received selective nerve root block or lumbar discectomy surgery after the diagnosis of primary LDH (defined as obvious LDH MRI findings and had symptoms or signs of LDH) | High | Age, sex, weight, BMI, height, smoking, generalized joint laxity | Phase II |
| Yang, 2020 [62]  China  Case-control | Cases: 384 Han Chinese patients with LDH recruited at the Second Affiliated Hospital of Inner Mongolia University from 2015-2017  Controls: 384 Han Chinese controls with or without back pain but without clinically lumbar disc degeneration presented on X-ray recruited at the same hospital  Exclusion not mentioned  Follow-up: NA  N=768 (41%)  NR (50y)  NR | Clinical LDH  Clinical  LDH was defined as lumbar pain, lumbar disc degeneration and herniation and/or unilateral or bilateral numbness of lower limb and confirmation on imaging (CT/MRI) | High | Collagen genes (COL1A1, COL9A3, COL2A1) | Phase II  COL9A3 (rs6122316): codominant OR 0.4 (0.2-0.8), recessive OR 0.4 (0.2-0.8)  >50y: COL1A1 (rs16970089) GA codominant OR 0.6 (0.4-0.9), GA-AA dominant OR 0.6 (0.4-0.9), log-additive OR 0.7 (0.5-0.9)  >50y: COL2A1 (rs6122316) AA recessive OR 0.4 (0.2-0.9)  ≤50 years: COL1A1 (rs16970089) AA recessive OR 3.2 (1.1-9.0)  Smoking: COL1A1(rs2071358) GT OR 2.6 (1.0-6.3), COL2A1(rs740024) AC OR 2.4 (1.1-5.3), COL2A1(rs740024) recessive 0.4 (0.2-0.9)  Drinking: COL2A1(rs740024) AC OR 5.4 (1.1-26.3)  In women:  COL1A1 (rs16970089): GA codominant OR 0.5 (0.3-0.8), dominant OR 0.6 (0.4-0.9), log-additive OR 0.7 (0.5-1.0)  COL2A1 (rs740024): CA codominant OR 0.5 (0.3-0.8), CA-AA dominant OR 0.5 (0.3-0.9) |
| Zhou, 2021 [97]  China (used finnish data)  Case-Control | Cases: 6,827 patients with sciatica from the FinnGen R4 consortium  Controls: 134,889 controls from the FinnGen R4 consortium  Exclusion not mentioned  Follow-up: NA  N=176,899 (NR)  NR (NR)  100% | Sciatica  Hospital  Sciatica was ICD-10 diagnosis codes M54.3, M54.4 and ICD-8 code 7170 | High | BMI, waist circumference, hip circumference, waist-hip ratio, whole-body fat mass, whole-body fat-free mass, whole body fat percentage, sedentary behaviour | Phase II  BMI: OR 1.3 (1.2-1.5)  Waist circumference: OR 1.4 (1.1–1.7)  Hip circumference: OR 1.3 (1.0-1.6) |
| ^a^ Sun, 2013 [98]  ^b^ Sun, 2011 [99]  ^97^China  Case-control | Cases: Orthopaedic and spine surgery hospital patients with LDH, 2006-2009  Controls: Healthy hospital patients with medical check-up  Follow-up: NA  N= ^a^ 1,008 (40%);  ^b^ 799 (40%)  NR (NR)  NR | Clinical LDH  Clinical  Cases were diagnosed by MRI of lumbar spine with clinical symptoms and signs | High | ^a^ Polymorphisms of cell death pathway genes FAS and FASL, and age, sex, height, weight, smoking, family history of LDH, occupational lumbar load  ^b^ Caspase 9 gene polymorphism | ^a^ Phase II  ^b^ Phase I |
| ^a^ Jacobsen, 2013 [100]  ^b^ Jacobsen, 2012 [101]  Norway  Case-control | Cases: Patients with LDH from two university hospitals from 2007-2009  Controls: Participants without a history of back disease from a general health survey (Nord-Trøndelag Health Study), matched for age, sex and smoking status  Follow-up: NA  N=510 (47%)  18-60y (41)  89% | Clinical LDH  Clinical  LDH defined as LDH on MRI with corresponding sciatica pain and positive straight leg raising test | High | ^a^ Matrix metalloproteinase (MMP) SNP MMP1 rs1799750 2G allele  ^b^ Catechol-O-methyltransferase (COMT) SNP rs4680, also known as COMT Val158Met  ^a,b^ Age, sex, smoking status, and treatment | ^a^ Phase II  ^b^ Phase I |
| Netterstrøm, 1989 [85]  Denmark  Cohort | All full-time male bus drivers employed on April 1, 1978 by 3 urban bus companies followed up from 1978-1984 for hospitalized LDH in the Danish National Patient Register  Follow-up: 7y  N=2,465 (0%)  20-69y (NR)  100% | Hospitalized LDH  Hospital  ICD discharge diagnosis codes for LDH (725.10-725.11) | High | Occupation | Phase I  Occupation: hospitalized LDH in male urban bus drivers compared to all Danish men: SMR 137 (105-176) |
| An, 1994 [102]  USA  Case-control | Cases: 163 consecutive surgical patients with LDH at Pennsylvania Hospital between 1987-1988  Controls: 205 inpatients without lumbar disc disease from medical and surgical services at the same hospital, matched on sex and age  Follow-up: NA  368 (39%)  16-78y (45)  NR | LDH surgery  Surgical  Confirmed LDH surgery case defined by meeting 3 criteria: i) prolapsed lumbar intervertebral disc was the primary diagnosis; ii) all were symptomatic and admitted for surgical management (100% surgically confirmed); iii) all had radiographic evidence (myelogram, CT scan or MRI) of intervertebral herniation | High | Smoking | Phase I  Smoking (current and ex-smokers v non-smokers) OR 2.2  Smoking (current smokers v non-smokers) OR 3.0  Other findings  Incorrect analysis for a matched CC study; compares proportion of current and ex-smokers among cases v controls instead of discordant pairs, results not sex- and age-adjusted |
| Lee, 2006 [103]  Korea  Case-control | Cases: 119 herniated lumbar disc levels in 111 adult patients who underwent LDH surgery between 2000-2002  Controls: 82 normal discs levels adjacent to the herniated levels in the same adult patients  Excluded patients who had not had pre-operative CT scans  Follow-up: NA  201 adult disc levels (37%)  40-49y (NR)  NR | LDH surgery  Surgical  Herniated disc level noted during open discectomy or percutaneous endoscopic discectomy for radiculopathy | High | Facet joint tropism | Phase I  NR; degree of facet tropism at the L4-L5 level was significantly greater in herniated discs than in normal discs (6.9±5.5° v 3.6±3.0°, p<0.001)  No significant difference found between herniated and normal discs at the L3-L4 and L5-S1 levels |
| Saftic, 2006 [104]  Croatia  Case-control | Cases: 67 adults from 9 villages on the Croatian islands of Rab, Vis, Lastovo, and Mljet, who had a positive history of LDH surgery  Controls: 268 adults matched on age, sex, and village of residence/immigrant status on a 4:1 basis  Excluded persons who underwent surgery of the lower spine due to other causes, such as degenerative changes or stenosis  Follow-up: NA  365 (NR)  ≥18y (NR)  NR | LDH surgery  Surgical  LDH surgery was a positive history of surgery due to lumbar intervertebral disc herniation at the level of L4/L5 or L5/S1 based on examination of medical histories and medical records | High | BMI, occupation type, intensity of physical labor at work, intensity of physical labour at home, smoking index, claudication index, self-assessed limitation in physical activity, level of education, socioeconomic status, and family history of lower spine surgery | Phase I  BMI (kg/m^2^): ≥25.7 v <25.7 OR 2.8 (1.1-4.5)  Occupation: involving hard physical labour (agriculture workers, soldiers, construction workers, mechanics or fishermen) v involving sitting or standing (clerks, lawyers, economists, tailors, waiters, cooks, salespersons, teachers, policemen, electricians, and housewives) OR 1.9 (1.1-3.8)  Intensity of physical labour at work: hard v sitting, easy or moderate) OR 2.9 (1.1-4.8)  Positive family history of spine surgery (having a parent who underwent spine surgery): yes v no OR 4.0 (1.9-6.1) |
| Kunakornsawat, 2007 [105]  Thailand  Case-control | Cases: 34 herniated lumbar disc levels in 34 adult patients who underwent discectomy at Lerdsin Hospital between 2001-2003  Controls: 34 normal discs levels adjacent to the herniated levels in the same patients  Excluded MRIs of persons who had associated spinal abnormality such as spina bifida, unilateral sacralization, enlarged transverse processes, or spondylolisthesis  Follow-up: NA  68 disc levels (35%)  23-45y (34)  NR | LDH surgery  Surgical  Patient aged <45y who underwent conventional discectomy for LDH (single protrusion or extrusion disc at L3-L4, L4-L5 or L5-S1), without previous surgery, and with symmetrical MRI scan of each disc level | High | Facet joint tropism | Phase I  Facet tropism at L4-L5 OR 1.8 (0.3-10)  Facet tropism at L5-S1 OR 1.7 (0.3-10)  Other findings  No association found between facet tropism and LDH in all levels |
| Paz Aparicio, 2011 [106]  Spain  Case-control | Cases: 50 patients with clinical LDH, 2007-2008  Controls: 129 orthopaedic patients admitted for primary hip or knee arthroplasty  Follow-up: NA  N=179 (60%)  Cases: 23-77y (44y)  Controls: 25-85y (69y)  NR | Clinical LDH  Clinical  Clinical symptoms suggestive of LDH and the condition confirmed by MRI. LDH symptoms were: (1) lower back pain, (2) pain, weakness or tingling in the legs, buttocks and feet, (3) difficulty in moving the lower back, (4) problems with bowel, bladder or erectile function in severe cases. LDH MRI findings were extension of the disk beyond margins of adjacent vertebral bodies. | High | SNPs of cytokines [IL-1a (-889 C/T); IL-1b (+3953 T/C); tumor necrosis factor (TNF) [(-308 G/A and -238 G/A)], endothelial nitric oxide synthase (eNOS) [(27 bp repeat in intron 4) and (-786 T/C)]; and inducible nitric oxide synthase (iNOS) (iNOS 22 G/A) | Phase I |
| Song, 2013 [107]  ^a^China, ^b^Finland, ^c^Japan  Case-control | Cases: 4,043 patients with lumbar degenerative disc disease from different cohorts  Controls: 28,599 controls  Exclusions not mentioned  Follow-up: NA  N= 32,642 (NR)  NR (NR)  NR  ^a^n=1,889 (NR)  ^b^n=5,434 (NR)  ^c^n=20,905 (NR) | ^a^Clinical LDH and sciatica  Clinical  LDH and sciatica with positive MRI imaging  ^b^Hospitalized sciatica  Hospital  No case definition mentioned  ^c^Clinical LDH and sciatica  Clinical  No case definition mentioned | High | Carbohydrate sulfotransferase 3 (CHST3) gene | ^a,b,c^ Phase I  CHST3 SNP(rs1245582): OR 1.2 (1.1-1.3)  CHST3 SNP(rs4148941): OR 1.3 (1.2-1.4)  CHST3 SNP(rs4148949): OR 1.3 (1.2-1.4) |
| Cong, 2014 [108]  China  Case-control | Cases: Hospital patients with surgically managed LDH  Controls: Healthy blood donors without LDH  Follow-up: NA  N=259 (0%)  14-49y (37y)  NR | Surgical LDH  Surgical  Symptomatic LDH defined as radicular pain with signs of positive nerve root tension or neurologic deficit, confirmatory imaging demonstrating LDH corresponding to their symptoms, and presence of symptoms for at least 6weeks | High | Aggrecan gene VNTR polymorphism and obesity | Phase I |
| Fei, 2017 [109]  China  Case-control | Cases: 100 young Chinese patients with LDH recruited from Peking University Third Hospital from 2012-2015  Controls: 100 age and sex matched asymptomatic volunteers without LBP, leg pain and other spinal disorders  Excluded those with complicated adolescent idiopathic scoliosis, spinal tumors, vertebral fractures, spondylolisthesis, other symptomatic spinal diseases, lower extremity disease, pelvic fractures, lumbar operation history or obviously forced posture caused by pain  Follow-up: NA  N=200 (34%)  18-35y (27y)  NR | Clinical LDH  Clinical  LDH diagnosis based on clinical an imaging findings | High | Sagittal alignment and pelvic morphology | Phase I  There is no difference in pelvic incidence between young Chinese patient with LDH and the normal population  Fatal flaw: Temporal sequence of exposure and outcome cannot be established |
| Keser, 2017 [110]  Turkey  Case-control | Cases: 50 patients with symptomatic LDH hospitalized at the Physical Medicine and Rehabilitation Clinic from 2015-2016  Controls: 50 age and sex matched controls with LBP but no finding of LDH on MRI recruited from the same clinic from 2015-2016  Excluded women in menopause and patients with conditions affecting bone mineral density such as malignancy, inflammatory joint diseases, hyperthyroidism, hyperparathyroidism, chronic liver disease, diabetes mellitus type 1, osteogenesis imperfecta, hypogonadism, chronic malnutrition, malabsorption, spinal stenosis, spondylolisthesis, compression fractures, patients with previous treatment for osteoporosis and patients being treated with cortisone, antiepileptic drugs or thyroid replacement medications  Follow-up: NA  N=100 (50%)  NR (38y)  NR | Hospitalized LDH  Hospital  LDH was defined as LBP or unilateral sciatica with MRI confirmed single-level disc herniation | High | Bone mineral density, smoking, BMI, waist circumference, 25 (OH) vitamin D_3_, parathormone, calcium, phosphorus, alkaline phosphatase | Phase I  No statistically significant differences in any measure of bone health could be detected. |
| Yaltirik, 2019 [111]  Turkey  Case-control | Cases: 108 patients with LDH followed-up by the Neurosurgery Department of Yeditepe University  Controls: 103 healthy individuals who never experienced back pain or radiculopathy  Excluded those with infection, trauma, congenital anomalies, osteoporosis, oncological pathologies, spinal stenosis, spondylolisthesis, vertebral fractures, or spinal deformities in their medical history  Follow-up: NA  N=211 (52%)  NR (40y)  NR | Clinical LDH  Clinical  LDH was defined as LBP and sciatica with MRI confirmed LDH | High | ACAN gene (c.6423T>C) variant | Phase I  No significant difference regarding distribution of ACAN (c.6423T>C) alleles was found between cases and controls |
| Wang, 2020 [112]  China  Case-control | Cases: 100 patients with single-level LDH L4/5 and 100 patients with single-level LDH L5/S1 of East Asian ancestry who had surgery for LDH from 2015-2019  Controls: 100 age and sex matched controls with CT of lumbar spine identified through record search of hospital  Excluded those with previous lumbar surgery, recurrent LDH, spinal tumors, infection, trauma, stenosis or spondylolysis on the lumbar spine  Follow-up: NA  N=300 (45%)  NR (49y)  NR | LDH surgery  Surgical  Patients with LDH surgery and MRI confirmed single-level LDH L4/5 or L5/S1 | High | Lumbar facet tropism on cephalad and caudad portions | Phase I  Facet tropism on cephalad portion L4/5 in age group ≥50y: no OR 1.0, yes OR 4.6 (1.6-15.1)  Asymmetry of ipsilateral cephalad and caudad facet joint in age group ≥50y: left side no OR 1.0, left side yes OR 4.6 (1.6-15.1), right side no OR 1.0. Right side no OR 1.0, yes OR 3.1 (1.1-9.5)  Other findings:  Facet angles of cephalad portion were larger than that of caudad portion at level L4/5. |
| Bailey, 2022 [90]  USA  Cohort | Twelve NASA astronauts spending 6 months in space from 2011-2018  Follow-up: 8m-2.5y  N=12 (17%)  NR (51y)  NR | Clinical LDH  Clinical  Clinical signs of LDH and confirmatory MRI scan | High | Multifidus muscle quality, spinal segment kinematics, spinal endplate irregularities | Phase I  Other findings:  Reduction of lean multifidus muscle of -6.2% at level L4/L5 and -7% at level L5/S1 associated with incidence of new disc herniation |
| Abbreviations: BMI, body mass index; cig, cigarettes; cm, centimeter; CT, computerized tomography; d, day; h, hour; ICD, international classification of diseases; kg, kilogram; LBP, low back pain; LDH, lumbar disc herniation; m, meter; mo, month; MRI, magnetic resonance imaging; MSK, musculoskeletal; N, study size; NA, not applicable; Nh, Newton-hours; NR, not reported; OR, odds ratio; ROB, risk of bias; RR, relative risk; SMR, standardized morbidity ratio; SNP, single nucleotide polymorphism; v, versus; VNTR, variable number of tandem repeats; wk, week; y, year | | | | | |

**References**

1. Heliövaara M (1987) Occupation and risk of herniated lumbar intervertebral disc or sciatica leading to hospitalization. J Chronic Dis 40:259–264. https://doi.org/10.1016/0021-9681(87)90162-7

2. Heliovaara M (1987) Body height, obesity, and risk of herniated lumbar intervertebral disc. Spine 12:469–472. https://doi.org/10.1097/00007632-198706000-00009

3. Heliövaara M, Knekt P, Aromaa A (1987) Incidence and risk factors of herniated lumbar intervertebral disc or sciatica leading to hospitalization. J Chronic Dis 40:251–258. https://doi.org/10.1016/0021-9681(87)90161-5

4. Bruske-Hohlfeld I, Merritt JL, Onofrio BM, et al (1990) Incidence of lumbar disc surgery: a population-based study in Olmsted County, Minnesota, 1950–1979. Spine 15:31–35. https://doi.org/10.1097/00007632-199001000-00009

5. Zitting P, Rantakallio P, Vanharanta H (1998) Cumulative incidence of lumbar disc diseases leading to hospitalization up to the age of 28 years. Spine 23:2337–43; discussion 2343-4. https://doi.org/10.1097/00007632-199811010-00017

6. Miranda H, Viikari-Juntura E, Martikainen R, et al (2002) Individual factors, occupational loading, and physical exercise as predictors of sciatic pain. Spine 27:1102–9. https://doi.org/10.1097/00007632-200205150-00017

7. Jhawar BS, Fuchs CS, Colditz GA, Stampfer MJ (2006) Cardiovascular risk factors for physician-diagnosed lumbar disc herniation. Spine J 6:684–691. https://doi.org/10.1016/j.spinee.2006.04.016

8. Mattila VM, Saarni L, Parkkari J, et al (2008) Early risk factors for lumbar discectomy: an 11-year follow-up of 57,408 adolescents. Eur Spine J 17:1317–1323. https://doi.org/10.1007/s00586-008-0738-2

9. Mattila VM, Sillanpää P, Visuri T, Pihlajamäki H (2009) Incidence and trends of low back pain hospitalisation during military service – An analysis of 387,070 Finnish young males. BMC Musculoskelet Disord 10:10. https://doi.org/10.1186/1471-2474-10-10

10. Hincapié CA, Tomlinson GA, Côté P, et al (2018) Chiropractic care and risk for acute lumbar disc herniation: a population-based self-controlled case series study. Eur Spine J 27:1526–1537. https://doi.org/10.1007/s00586-017-5325-y

11. Wahlström J, Burström L, Johnson PW, et al (2018) Exposure to whole-body vibration and hospitalization due to lumbar disc herniation. Int Arch Occup Environ Health 91:689–694. https://doi.org/10.1007/s00420-018-1316-5

12. Balling M, Holmberg T, Petersen CB, et al (2019) Total sitting time, leisure time physical activity and risk of hospitalization due to low back pain: The Danish Health Examination Survey cohort 2007–2008. Scand J Public Health 47:45–52. https://doi.org/10.1177/1403494818758843

13. Brauer C, Mikkelsen S, Pedersen EB, et al (2020) Occupational lifting predicts hospital admission due to low back pain in a cohort of airport baggage handlers. Int Arch Occup Environ Health 93:111–122. https://doi.org/10.1007/s00420-019-01470-z

14. Jung J-M, Lee SU, Hyun S-J, et al (2020) Trends in incidence and treatment of herniated lumbar disc in Republic of Korea : A nationwide database study. J Korean Neurosurg Soc 63:108–118. https://doi.org/10.3340/jkns.2019.0075

15. Hurme M, Alaranta H, Törmä T, Einola S (1983) Operated lumbar disc herniation: epidemiological aspects. Ann Chir Gynaecol 72:33–6

16. Heikkilä JK, Heikkilä K, Rita H, et al (1989) Genetic and environmental factors in sciatica evidence from a nationwide panel of 9365 adult twin pairs. Ann Med 21:393–398. https://doi.org/10.3109/07853898909149227

17. Riihimaki H, Wickström G, Hanninen K, Luopajarvi T (1989) Predictors of sciatic pain among concrete reinforcement workers and house painters--a five-year follow-up. Scand J Work Environ Health 15:415–423. https://doi.org/10.5271/sjweh.1836

18. Jørgensen S, Hein HO, Gyntelberg F (1994) Heavy lifting at work and risk of genital prolapse and herniated lumbar disc in assistant nurses. Occup Med 44:47–49. https://doi.org/10.1093/occmed/44.1.47

19. Riihimäki H, Viikari-Juntura E, Moneta G, et al (1994) Incidence of sciatic pain among men in machine operating, dynamic physical work, and sedentary work. Spine 19:138–142. https://doi.org/10.1097/00007632-199401001-00003

20. Leino-Arjas P, Kaila-Kangas L, Kauppinen T, et al (2004) Occupational exposures and inpatient hospital care for lumbar intervertebral disc disorders among Finns. Am J Ind Med 46:513–520. https://doi.org/10.1002/ajim.20084

21. Leino-Arjas P, Kaila-Kangas L, Keskimäki I, et al (2002) Inpatient hospital care for lumbar intervertebral disc disorders in Finland in relation to education, occupational class, income, and employment. Public Health 116:272–278. https://doi.org/10.1038/sj.ph.1900868

22. Leclerc A (2003) Personal and occupational predictors of sciatica in the GAZEL cohort. Occup Med 53:384–391. https://doi.org/10.1093/occmed/kqg072

23. Jarvik JG, Hollingworth W, Heagerty PJ, et al (2005) Three-year incidence of low back pain in an initially asymptomatic cohort. Spine 30:1541–1548. https://doi.org/10.1097/01.brs.0000167536.60002.87

24. Suri P, Boyko EJ, Goldberg J, et al (2014) Longitudinal associations between incident lumbar spine MRI findings and chronic low back pain or radicular symptoms: retrospective analysis of data from the longitudinal assessment of imaging and disability of the back (LAIDBACK). BMC Musculoskelet Disord 15:152. https://doi.org/10.1186/1471-2474-15-152

25. Sørensen IG, Jacobsen P, Gyntelberg F, Suadicani P (2011) Occupational and other predictors of herniated lumbar disc disease—A 33-year follow-up in the Copenhagen Male Study. Spine 36:1541–1546. https://doi.org/10.1097/BRS.0b013e3181f9b8d4

26. Jørgensen MB, Holtermann A, Gyntelberg F, Suadicani P (2013) Physical fitness as a predictor of herniated lumbar disc disease – a 33-year follow-up in the Copenhagen male study. BMC Musculoskelet Disord 14:86. https://doi.org/10.1186/1471-2474-14-86

27. Wahlström J, Burström L, Nilsson T, Järvholm B (2012) Risk factors for hospitalization due to lumbar disc disease. Spine 37:1334–1339. https://doi.org/10.1097/BRS.0b013e31824b5464

28. Bovenzi M, Schust M, Menzel G, et al (2015) A cohort study of sciatic pain and measures of internal spinal load in professional drivers. Ergonomics 58:1088–1102. https://doi.org/10.1080/00140139.2014.943302

29. Chan F-K, Hsu C-C, Lin H-J, et al (2018) Physicians as well as nonphysician health care professionals in Taiwan have higher risk for lumbar herniated intervertebral disc than general population. Medicine 97:e9561. https://doi.org/10.1097/MD.0000000000009561

30. Fouquet N, Bodin J, Chazelle E, et al (2018) Use of multiple data sources for surveillance of work-related chronic low-back pain and disc-related sciatica in a french region. Ann Work Expo Health 62:530–546. https://doi.org/10.1093/annweh/wxy023

31. Han M, Park S, Park JH, et al (2018) Do police officers and firefighters have a higher risk of disease than other public officers? A 13-year nationwide cohort study in South Korea. BMJ Open 8:e019987. https://doi.org/10.1136/bmjopen-2017-019987

32. Kim Y-K, Kang D, Lee I, Kim S-Y (2018) Differences in the incidence of symptomatic cervical and lumbar disc herniation according to age, sex and national health insurance eligibility: A pilot study on the disease’s association with work. Int J Environ Res Public Health 15:2094. https://doi.org/10.3390/ijerph15102094

33. Knox JB, Deal JB, Knox JA (2018) Lumbar disc herniation in military helicopter pilots vs. matched controls. Aerosp Med Hum Perform 89:442–445. https://doi.org/10.3357/AMHP.4935.2018

34. Huang W, Weng S, Hsu C, et al (2019) Comparison of the risk of developing lumbar herniated intervertebral disc between dentists and other occupations: A nationwide population‐based study in Taiwan. J Occup Health 61:227–234. https://doi.org/10.1002/1348-9585.12036

35. Jäntti S, Ponkilainen V, Mäntymäki H, et al (2022) Trends in emergency department visits due to back pain and spine surgeries during the COVID-19 pandemic in Finland. Medicine 101:e29496. https://doi.org/10.1097/MD.0000000000029496

36. Nyrhi L, Kuitunen I, Ponkilainen V, et al (2023) Incidence of lumbar discectomy during pregnancy and within 12 months post-partum in Finland between 1999 and 2017: a retrospective register-based cohort study. Spine J 23:287–294. https://doi.org/10.1016/j.spinee.2022.10.015

37. Seidler A, Bolm-Audorff U, Siol T, et al (2003) Occupational risk factors for symptomatic lumbar disc herniation; a case-control study. Occup Environ Med 60:821–830. https://doi.org/10.1136/oem.60.11.821

38. Seidler A, Bergmann A, Jäger M, et al (2009) Cumulative occupational lumbar load and lumbar disc disease – results of a German multi-center case-control study (EPILIFT). BMC Musculoskelet Disord 10:48. https://doi.org/10.1186/1471-2474-10-48

39. Bergmann A, Bolm-Audorff U, Ditchen D, et al (2017) Do occupational risks for low back pain differ from risks for specific lumbar disc diseases?: Results of the German Lumbar Spine Study (EPILIFT). Spine 42:E1204–E1211. https://doi.org/10.1097/BRS.0000000000002296

40. Schumann B, Bolm-Audorff U, Bergmann A, et al (2010) Lifestyle factors and lumbar disc disease: results of a German multi-center case-control study (EPILIFT). Arthritis Res Ter 12:R193. https://doi.org/10.1186/ar3164

41. Seidler A, Euler U, Bolm-Audorff U, et al (2011) Physical workload and accelerated occurrence of lumbar spine diseases: risk and rate advancement periods in a German multicenter case–control study. Scand J Work Environ Health 37:30–36. https://doi.org/10.5271/sjweh.3121

42. Kelsey JL (1975) An epidemiological study of the relationship between occupations and acute herniated lumbar intervertebral discs. Int J Epidemiol 4:197–205. https://doi.org/10.1093/ije/4.3.197

43. Kelsey JL (1975) An epidemiological study of acute herniated lumbar intervertebral discs. Rheumatol Rehabil 14:144–59. https://doi.org/10.1093/rheumatology/14.3.144

44. Kelsey JL, Hardy RJ (1975) Driving of motor vehicles as a risk factor for acute herniated lumbar intervertebral disc. Am J Epidemiol 102:63–73. https://doi.org/10.1093/oxfordjournals.aje.a112135

45. Kelsey JL, Ostfeld AM (1975) Demographic characteristics of persons with acute herniated lumbar intervertebral disc. J Chronic Dis 28:37–50. https://doi.org/10.1016/0021-9681(75)90047-8

46. Kelsey JL, Greenberg RA, Hardy RJ, Johnson MF (1975) Pregnancy and the syndrome of herniated lumbar intervertebral disc; an epidemiological study. Yale J Biol Med 48:361–8

47. Kelsey JL, Githens PB, White AA, et al (1984) An epidemiologic study of lifting and twisting on the job and risk for acute prolapsed lumbar intervertebral disc. J Orthop Res 2:61–66. https://doi.org/10.1002/jor.1100020110

48. Kelsey JL, Githens PB, OʼConner T, et al (1984) Acute prolapsed lumbar intervertebral disc. An epidemiologic study with special reference to driving automobiles and cigarette smoking. Spine 9:608–613. https://doi.org/10.1097/00007632-198409000-00012

49. Mundt DJ, Kelsey JL, Golden AL, et al (1993) An epidemiologic study of non-occupational lifting as a risk factor for herniated lumbar intervertebral disc. Spine 18:595–602. https://doi.org/10.1097/00007632-199304000-00012

50. Mundt DJ, Kelsey JL, Golden AL, et al (1993) An epidemiologic study of sports and weight lifting as possible risk factors for herniated lumbar and cervical discs. Am J Sports Med 21:854–860. https://doi.org/10.1177/036354659302100617

51. Pietri-Taleb F, Riihimäki H, Viikari-Juntura E, et al (1995) The role of psychological distress and personality in the incidence of sciatic pain among working men. Am J Public Health 85:541–545. https://doi.org/10.2105/AJPH.85.4.541

52. Zhang Y, Zhao Y, Wang M, et al (2016) Serum lipid levels are positively correlated with lumbar disc herniation—a retrospective study of 790 Chinese patients. Lipids Health Dis 15:80. https://doi.org/10.1186/s12944-016-0248-x

53. Bjornsdottir G, Benonisdottir S, Sveinbjornsson G, et al (2017) Sequence variant at 8q24.21 associates with sciatica caused by lumbar disc herniation. Nat Commun 8:14265. https://doi.org/10.1038/ncomms14265

54. Dong Q, Ren G, Zhang K, et al (2018) Genetic polymorphisms of ALDH2 are associated with lumbar disc herniation in a Chinese Han population. Sci Rep 8:13079. https://doi.org/10.1038/s41598-018-31491-6

55. Zhu Y, Jia H, Li J, et al (2018) Associations between variants in BDNF/BDNFOS gene and lumbar disc herniation risk among Han Chinese people. Sci Rep 8:12782. https://doi.org/10.1038/s41598-018-31146-6

56. Jing R, Liu Y, Guo P, et al (2018) Evaluation of common variants in matrix metalloproteinase-9 gene with lumbar disc herniation in Han Chinese population. Genet Test Mol Biomarkers 22:622–629. https://doi.org/10.1089/gtmb.2018.0080

57. Li L, Ni D, Zhu F (2018) No association between VDR gene polymorphisms and lumbar disc herniation in a Chinese population. Int J Clin Exp Med

58. Hu B, Xing W, Li F, et al (2019) Association of glypican‐6 polymorphisms with lumbar disk herniation risk in the Han Chinese population. Mol Genet Genomic Med 7:. https://doi.org/10.1002/mgg3.747

59. Ji D, Xing W, Li F, et al (2019) Correlation of EYS polymorphisms with lumbar disc herniation risk among Han Chinese population. Mol Genet Genomic Med 7:. https://doi.org/10.1002/mgg3.890

60. Liu K, Huo H, Jia W, et al (2021) RAB40C gene polymorphisms rs62030917 and rs2269556 are associated with an increased risk of lumbar disc herniation development in the Chinese Han population. J Gene Med 23:. https://doi.org/10.1002/jgm.3252

61. Wu J, Sun Y, Xiong Z, et al (2020) Association of GSDMC polymorphisms with lumbar disc herniation among Chinese Han population. Int J Immunogenet 47:546–553. https://doi.org/10.1111/iji.12488

62. Yang X, Jia H, Xing W, et al (2020) Multiple variants in collagen genes are associated with the susceptibility to lumbar disc herniation in the Chinese population. Eur Spine J 29:1709–1716. https://doi.org/10.1007/s00586-020-06299-6

63. Hu X, Hao D, Yin J, et al (2022) Association between MIR31HG polymorphisms and the risk of lumbar disc herniation in Chinese Han population. Cell Cycle 21:2109–2120. https://doi.org/10.1080/15384101.2022.2087281

64. Han P, Jiang F, Zhang L (2023) The role of ADAMTS6 and ADAMTS17 polymorphisms in susceptibility to lumbar disc herniation in Chinese Han population. Eur Spine J 32:1106–1114. https://doi.org/10.1007/s00586-023-07586-8

65. Wu Y, Bai M, Yu Y, et al (2023) Association of LINC-PINT polymorphisms with lumbar disc herniation risk among Chinese Han population: a case control study. J Orthop Surg Res 18:585. https://doi.org/10.1186/s13018-023-04052-5

66. Yang X, Guo X, Huang Z, et al (2019) CHRNA5/CHRNA3 gene cluster is a risk factor for lumbar disc herniation: a case-control study. J Orthop Surg Res 14:243. https://doi.org/10.1186/s13018-019-1254-2

67. Zhu Y, Li S, Sun Y, et al (2019) IL1R1 polymorphisms are associated with lumbar disc herniation risk in the northwestern Chinese Han population. Medical Science Monitor 25:3728–3738. https://doi.org/10.12659/MSM.913563

68. Tai A, Zhu M, Qilimuge H, et al (2020) Genetic polymorphisms of IL1RN were associated with lumbar disk herniation risk in a Chinese Han population. Mol Genet Genomic Med 8:. https://doi.org/10.1002/mgg3.1247

69. Luo Y, Wang J, Pei J, et al (2020) Interactions between the MMP‐3 gene rs591058 polymorphism and occupational risk factors contribute to the increased risk for lumbar disk herniation: A case‐control study. J Clin Lab Anal 34:. https://doi.org/10.1002/jcla.23273

70. Fidan F, Balaban M, Hatipoğlu ŞC, Veizi E (2022) Is lumbosacral transitional vertebra associated with lumbar disc herniation in patients with low back pain? Eur Spine J 31:2907–2912. https://doi.org/10.1007/s00586-022-07372-y

71. Noponen-Hietala N, Virtanen I, Karttunen R, et al (2005) Genetic variations in IL6 associate with intervertebral disc disease characterized by sciatica. Pain 114:186–194. https://doi.org/10.1016/j.pain.2004.12.015

72. Mio F, Chiba K, Hirose Y, et al (2007) A functional polymorphism in COL11A1, which encodes the α1 chain of type XI collagen, is associated with susceptibility to lumbar disc herniation. Am J Hum Genet 81:1271–1277. https://doi.org/10.1086/522377

73. Virtanen IM, Song YQ, Cheung KMC, et al (2007) Phenotypic and population differences in the association between CILP and lumbar disc disease. J Med Genet 44:285–288. https://doi.org/10.1136/jmg.2006.047076

74. Hirose Y, Chiba K, Karasugi T, et al (2008) A functional polymorphism in THBS2 that affects alternative splicing and MMP binding is associated with lumbar-disc herniation. J Med Genet 82:1122–1129. https://doi.org/10.1016/j.ajhg.2008.03.013

75. Karasugi T, Semba K, Hirose Y, et al (2009) Association of the tag SNPs in the human SKT gene (KIAA1217) with lumbar disc herniation. J Bone Miner Res 24:1537–1543. https://doi.org/10.1359/jbmr.090314

76. Cong L, Pang H, Xuan D, Tu GJ (2010) Association between the expression of aggrecan and the distribution of aggrecan gene variable number of tandem repeats with symptomatic lumbar disc herniation in Chinese Han of Northern China. Spine 35:1371–1376. https://doi.org/10.1097/BRS.0b013e3181c4e022

77. Mu J, Ge W, Zuo X, et al (2014) A SNP in the 5′UTR of GDF5 is associated with susceptibility to symptomatic lumbar disc herniation in the Chinese Han population. Eur Spine J 23:498–503. https://doi.org/10.1007/s00586-013-3059-z

78. Huang X, Chen F, Zhao J, et al (2017) Interleukin 6 (IL-6) and IL-10 promoter region polymorphisms are associated with risk of lumbar disc herniation in a northern Chinese Han population. Genet Test Mol Biomarkers 21:17–23. https://doi.org/10.1089/gtmb.2016.0189

79. Jiang H, Yang Q, Jiang J, et al (2017) Association between COL11A1 (rs1337185) and ADAMTS5 (rs162509) gene polymorphisms and lumbar spine pathologies in Chinese Han population: an observational study. BMJ Open 7:e015644. https://doi.org/10.1136/bmjopen-2016-015644

80. Ghandhari H, Ameri E, Hasani H, et al (2018) Is facet tropism associated with increased risk of disc herniation in the lumbar spine? Asian Spine J 12:428–433. https://doi.org/10.4184/asj.2018.12.3.428

81. Wang Y, Wang H, Lv F, et al (2018) Asymmetry between the superior and inferior endplates is a risk factor for lumbar disc degeneration. J Orthop Res 36:2469–2475. https://doi.org/10.1002/jor.23906

82. Withanage ND, Perera S, Peiris H, Athiththan LV (2018) Serum 25-hydroxyvitamin D, serum calcium and vitamin D receptor (VDR) polymorphisms in a selected population with lumbar disc herniation—A case control study. PLoS One 13:e0205841. https://doi.org/10.1371/journal.pone.0205841

83. Zhou Q, Teng D, Zhang T, et al (2018) Association of facet tropism and orientation with lumbar disc herniation in young patients. Neurol Sci 39:841–846. https://doi.org/10.1007/s10072-018-3270-0

84. Bongers PM, Boshuizen HC, Hulshof CTJ, Koemeester AP (1988) Back disorders in crane operators exposed to whole-body vibration. Int Arch Occup Environ Health 60:129–137. https://doi.org/10.1007/BF00381494

85. Netterstrøm B, Juel K (1989) Low back trouble among urban bus drivers in Denmark. Scand J Soc Med 17:203–206. https://doi.org/10.1177/140349488901700211

86. Rivinoja AE, Paananen M V., Taimela SP, et al (2011) Sports, smoking, and overweight during adolescence as predictors of sciatica in adulthood: a 28-year follow-up study of a birth cohort. Am J Epidemiol 173:890–897. https://doi.org/10.1093/aje/kwq459

87. Roquelaure Y, Fouquet N, Ha C, et al (2011) Epidemiological surveillance of lumbar disc surgery in the general population: A pilot study in a French region. Joint Bone Spine 78:298–302. https://doi.org/10.1016/j.jbspin.2010.08.008

88. Chung Y-C, Hung C-T, Li S-F, et al (2013) Risk of musculoskeletal disorder among Taiwanese nurses cohort: a nationwide population-based study. BMC Musculoskelet Disord 14:144. https://doi.org/10.1186/1471-2474-14-144

89. Makovicka JL, Patel KA, Deckey DG, et al (2019) Lower back injuries in National Collegiate Athletic Association football players: A 5-season epidemiological study. Orthop J Sports Med 7:232596711985262. https://doi.org/10.1177/2325967119852625

90. Bailey JF, Nyayapati P, Johnson GTA, et al (2022) Biomechanical changes in the lumbar spine following spaceflight and factors associated with postspaceflight disc herniation. Spine J 22:197–206. https://doi.org/10.1016/j.spinee.2021.07.021

91. Hrubec Z, Nashold BS (1975) Epidemiology of lumbar disc lesions in the military in World War II. Am J Epidemiol 102:367–76

92. Chibnall JT, Tait RC, Andresen EM, Hadler NM (2006) Race differences in diagnosis and surgery for occupational low back injuries. Spine 31:1272–1275. https://doi.org/10.1097/01.brs.0000217584.79528.9b

93. Zhang Y, Sun Z, Zhang Z, et al (2009) Risk factors for lumbar intervertebral disc herniation in Chinese population: a case-control study. Spine 34:E918–E922. https://doi.org/10.1097/BRS.0b013e3181a3c2de

94. Zhang Y-G, Zhang F, Sun Z, et al (2013) A controlled case study of the relationship between environmental risk factors and apoptotic gene polymorphism and lumbar disc herniation. Am J Pathol 182:56–63. https://doi.org/10.1016/j.ajpath.2012.09.013

95. Chiang S-L, Lin C-H, Tsai S-H, et al (2014) Cigarette smoking dose as a predictor of need for surgical intervention in patients with lumbar disk herniation. J Med Sci 34:23. https://doi.org/10.4103/1011-4564.129387

96. Lee GW, Lee S-M, Suh B-G (2015) The impact of generalized joint laxity on the occurrence and disease course of primary lumbar disc herniation. Spine J 15:65–70. https://doi.org/10.1016/j.spinee.2014.06.028

97. Zhou J, Mi J, Peng Y, et al (2021) Causal  associations of obesity with the intervertebral degeneration, low back pain, and sciatica: A two-sample Mendelian randomization study. Front Endocrinol 12:. https://doi.org/10.3389/fendo.2021.740200

98. Sun Z, Ling M, Chang Y, et al (2013) Single-nucleotide gene polymorphisms involving cell death pathways: a study of Chinese patients with lumbar disc herniation. Connect Tissue Res 54:55–61. https://doi.org/10.3109/03008207.2012.734878

99. Sun Z-M, Ling M, Huo Y, et al (2011) Caspase 9 gene polymorphism and susceptibility to lumbar disc disease in the Han population in northern China. Connect Tissue Res 52:198–202. https://doi.org/10.3109/03008207.2010.510914

100. Jacobsen LM, Schistad EI, Storesund A, et al (2013) The MMP1 rs1799750 2G allele is associated with increased low back pain, sciatica, and disability after lumbar disk herniation. Clin J Pain 29:967–971. https://doi.org/10.1097/AJP.0b013e31827df7fd

101. Jacobsen LM, Schistad EI, Storesund A, et al (2012) The COMT rs4680 Met allele contributes to long-lasting low back pain, sciatica and disability after lumbar disc herniation. Eur J Pain 16:1064–1069. https://doi.org/10.1002/j.1532-2149.2011.00102.x

102. An HS, Silveri CP, Simpson JM, et al (1994) Comparison of smoking habits between patients with surgically confirmed herniated lumbar and cervical disc disease and controls. J Spinal Disord 7:369–73

103. Lee DY, Ahn Y, Lee S-H (2006) The influence of facet tropism on herniation of the lumbar disc in adolescents and adults. J Bone Joint Surg Br 88:520–3. https://doi.org/10.1302/0301-620X.88B4.16996

104. Saftić R, Grgić M, Ebling B, Splavski B (2006) Case-control study of risk factors for lumbar intervertebral disc herniation in Croatian island populations. Croat Med J 47:593–600

105. Kunakornsawat S, Ngamlamaidt K, Tungsiripat R, Prasartritha T (2007) The relationship of facet tropism to lumbar disc herniation. J Med Assoc Thai 90:1337–41

106. Paz Aparicio J, Fernández Bances I, López-Anglada Fernández E, et al (2011) The IL-1β (+3953 T/C) gene polymorphism associates to symptomatic lumbar disc herniation. Eur Spine J 20:383–389. https://doi.org/10.1007/s00586-011-1915-2

107. Song Y-Q, Karasugi T, Cheung KMC, et al (2013) Lumbar disc degeneration is linked to a carbohydrate sulfotransferase 3 variant. J Clin Invest 123:4909–4917. https://doi.org/10.1172/JCI69277

108. Cong L, Zhu Y, Pang H, Guanjun TU (2014) The interaction between aggrecan gene VNTR polymorphism and obesity in predicting incident symptomatic lumbar disc herniation. Connect Tissue Res 55:384–390. https://doi.org/10.3109/03008207.2014.959117

109. Fei H, Li W, Sun Z, et al (2017) Analysis of spino-pelvic sagittal alignment in young Chinese patients with lumbar disc herniation. Orthop Surg 9:271–276. https://doi.org/10.1111/os.12340

110. Keser N, Atici A, Celikoglu E, et al (2017) Effect of bone mineral density on lumbar discs in young adults. Medicine 96:e7906. https://doi.org/10.1097/MD.0000000000007906

111. Yaltirik CK, Timirci-Kahraman Özlem, Gulec-Yilmaz S, et al (2019) The evaluation of proteoglycan levels and the possible role of of ACAN gene (c.6423T>C) variant in patients with lumbar disc degeneration disease. In Vivo (Brooklyn) 33:413–417. https://doi.org/10.21873/invivo.11488

112. Wang Y, Li D, Zhu M, et al (2020) Lumbar facet tropism on different facet portions and asymmetry between ipsilateral cephalad and caudad portions: their correlations with L4/5 and L5/S1 lumbar disc herniation. Spine 45:E1312–E1318. https://doi.org/10.1097/BRS.0000000000003614
