# Supplementary material for: Incidence of and risk factors for lumbar disc herniation with radiculopathy in adults: a systematic review
Source: Eur Spine J. 2024 Oct 25;34(1):263–94. doi: 10.1007/s00586-024-08528-8 (PMC11754359; doi:10.1007/s00586-024-08528-8)
Supplement: Supplementary file 2 — Supplementary file2 (DOCX 294 KB) [file 586_2024_8528_MOESM2_ESM.docx]

**Detailed synthesis of risk factors**

Below we report our detailed findings according to risk factor domains and prioritised by risk of bias (low risk of bias representing the best available evidence) and the hierarchical phases of evidence, with phase III studies presenting the strongest (confirmatory) evidence of association. Throughout this section, we highlight risk factor estimates (point and 95% CI estimates) from low risk of bias phase III studies. All estimates are detailed in Appendix 4 (Online Supplementary Material).

***Sociodemographics***

**Low risk of bias studies (N=4):** There were no phase III studies that examined the risk of LDH by different sociodemographic characteristics. Evidence from two phase II studies [11, 43] indicate that the incidence of LDH with radiculopathy increases with age, peaking about the fourth and fifth decades of life, and decreasing in later life. Three phase II studies [11, 41, 67] reported an increased risk for men, while one phase II study [24] reported an increased risk for women. Miranda et al. found no evidence of an association between sex and the development of clinical LDH with radiculopathy (sciatica) [43]. Heliövaara et al. suggested a higher incidence of LDH in middle class men compared to higher social class [9–11] while another phase II study reported no association between socioeconomic background and the incidence of LDH surgery in men and women in Finland [41].

**Moderate risk of bias studies (N=24):** Five studies (one phase III [36]) suggested an increased risk for LDH with radiculopathy with age, peaking at the fourth and fifth decade of life [33, 34, 36, 37, 59], whereas five studies reported no association with age among male worker cohorts and Han Chinese patients [21, 22, 35, 50, 56]. Four studies reported no association between sex and LDH with radiculopathy [18, 21, 22, 33, 35, 56], while two studies found an increased risk for men compared to women [26, 27, 30–32, 36, 37], and two, an increased risk for women [38, 40]. Evidence from one phase III [36] and one phase II [37] study suggested that the incidence of LDH with radiculopathy decreases with years of education, while two phase II studies [28, 50] found no association between education and the risk of LDH. Leino-Arjas et al. found an increased incidence of hospital care due to LDH across lower quintiles of personal net income compared to the highest quintile in their phase III analysis [36], whereas they reported a greater risk of hospitalised LDH among those with the highest quartile of personal net income compared to those with the lowest quartile in their phase II analysis [37].

***Body mass index, anthropometrics, and genetics***

**Low risk of bias studies (N=4):** One phase II study found an association between obesity and LDH with radiculopathy in women, but not in men [41]. Another phase II study reported a positive association in men, but no association in women [10]. One phase III study found weak associations between higher BMI categories of overweight and obesity, compared with BMI <21.9 kg/m^2^, and the incidence of LDH with radiculopathy (BMI 25.0-26.9 kg/m^2^, RR 1.1 [1.0-1.3]; 27.0-28.9 kg/m^2^, RR 1.2 [1.0-1.4]; ≥29 kg/m^2^, RR 1.1 [1.0-1.3]) [19], while another phase II study found no association [9–11, 43]. Heliövaara et al. (phase II) reported no evidence of an association between height and the risk of LDH [9–11].

**Moderate risk of bias studies (N=28):** Four studies indicated that the incidence of LDH with radiculopathy increases with higher BMI and/or obesity compared with healthy weight [36, 51, 52, 59], while six found no association [21, 26–32, 35, 55, 56]. Three studies reported a positive association between body height and risk of LDH [35, 55, 59], while two studies reported no association [28, 51]. One study reported an indication of a positive association between body height and risk of LDH in women, but a negative association in men [26, 27, 30–32]. We found evidence of an increased risk of LDH with radiculopathy in those with facet tropism [7, 65], asymmetry of endplates [60], lumbar transitional vertebra [6]. We found preliminary evidence of an ethnic association among people of Sri Lankan ethnicity with a 25-hydroxyvitamin D deficiency [61], and a genetic predisposition for LDH with radiculopathy, although results varied depending on the gene models used to estimate risk [4, 5, 8, 13–16, 20–22, 25, 38–40, 42, 44, 47, 57, 61–64, 66].

***Health conditions, prior pain, and comorbidities***

**Low risk of bias studies (N=5):** Jhawar et al. found phase III evidence that the presence of cardiovascular risk factors (vs. absence of each risk factor), including diabetes (RR 1.5 [1.2-2.0]), high cholesterol (RR 1.3 [1.1-1.4]), hypertension (RR 1.3 [1.1-1.4]), and a family history of a parent who had suffered a myocardial infarction before age 60 (RR 1.1 [1.0-1.3]), were associated with an increased risk of LDH with radiculopathy in women [19]. A history of low back accidents or low back injuries, chronic disease or disability, and chronic cough or respiratory symptoms were not associated with LDH with radiculopathy [11, 41, 43]^.^ No evidence of an excess risk for early LDH surgery after a chiropractic care visits compared to primary care physician visits was found (chiropractic care IRR 12.9 [7.2-23.3], primary care physician IRR 14.5 [9.9-21.2]) [12].

**Moderate risk of bias studies (N=12):** No evidence of an association was found between LDH with radiculopathy and presence of neck pain [35]; lumbar spine degeneration [51]; and arthritis [56]. Four studies reported an increased incidence of LDH with radiculopathy in those with a history of back symptoms, low back pain, sciatica and LDH with radiculopathy [35, 50, 51, 56], while one study found no association [36, 37]. One study found an association of chronic cough and respiratory symptoms in women[26], while one study found no association [28]**.**

***Health behaviours***

**Low risk of bias studies (N=5):** Evidence from one phase III ^29^and two phase II studies [41, 43] suggests that the incidence of LDH with radiculopathy increases with smoking (phase III, [19] current smoker vs. nonsmoker RR 1.4 [1.3-1.5]). However, one phase II study [9–11] found no association between smoking and the risk of LDH with radiculopathy. One study found no association between the incidence of LDH with radiculopathy and drinking [41]. Balling et al. (phase III) [1] found an increased incidence of LDH associated with moderate physical activity during leisure time (i.e., exercise, endurance training or heavy gardening for at least 4 hours per week), compared with light physical activity (walking, bicycling, or other light activities for a minimum of 4 hours per week) (HR 1.3 [1.0-1.6]). Miranda et al. reported an increased incidence of clinical LDH (sciatica) associated with walking but a decreased incidence associated with jogging [43]. Another study found a positive association between the frequency of participation in sports clubs (4-5 times per week) and risk of LDH surgery in women, but not in men [41]. Finally, Heliövaara et al. (phase II) found no association between physical or sports activity and the incidence of LDH [9–11].

**Moderate risk of bias studies (N=16):** Six studies found an association between the incidence of LDH with radiculopathy and smoking [28, 38, 40, 46, 48, 59], while five studies reported no association [21, 22, 35, 51, 55], and one a negative association [64]. One study suggested a higher risk of LDH with radiculopathy with alcohol consumption [40], while two studies found no association [22, 55]; and two other studies found a negative association [38, 64]. Two studies reported indications of negative associations between physical activity and LDH with radiculopathy [22, 26, 27, 30–32], while five studies found no association between physical fitness, physical or sports activity, and the incidence of LDH with radiculopathy [23, 28, 35, 45, 55].

***Personal physical factors***

**Low risk of bias studies (N=1):** One phase II study reported no association between personal car driving and incidence of LDH with radiculopathy among workers [43].

**Moderate risk of bias studies (N=6):** There was evidence suggesting a higher incidence of LDH with radiculopathy associated with do-it-yourself activities [35]; nonoccupational lifting and bending [46]; lifting children [46]; and soft bed types [22]. Three studies suggest a higher incidence of LDH with radiculopathy in persons driving non-Japanese and non-Swedish cars, as well as personal driving, while there was no evidence of associations found for driving patterns and personal driving in workers and healthcare patients [28, 31, 46].

***Personal psychosocial factors***

**Low risk of bias studies (N=3):** Two phase II studies from Finland found an increased incidence of LDH with radiculopathy in those who reported mental stress “to some extent” or “rather much or much” [43]; and a greater number of psychological distress symptoms in women, but not in men [9–11]. However, one other study reported no association between the risk of LDH with radiculopathy and personal mental stress [41].

**Moderate risk of bias studies (N=6):** Two studies suggested an association of LDH with hysteria in blue-collar workers [49], and depression among outpatients in a Veterans Affairs healthcare system [56]. Four studies found no associations between the risk of LDH with radiculopathy and personal mental stress [51, 55]; psychological well-being [35]; or number of stressful life events in the previous year [26, 27, 30–32].

***Occupational physical factors***

**Low risk of bias studies (N=5):** In their phase III study, Wahlström et al. reported a positive association among construction workers with moderate to high whole-body vibration exposure compared with nonexposed white-collar workers and foremen (RR 1.4 [1.1-1.6]) [58]; while Brauer et al. found no phase III evidence of an association between cumulative years of employment as an airport baggage-handler compared with a non-baggage handler and LDH with radiculopathy (incidence rate ratio 0.8 [0.6-1.2]) [3]. Miranda et al. found an association of trunk twisting movements and the incidence of LDH in Finnish forestry industry workers in their phase II study, but no association with working with the trunk flexed forward [43]. No evidence of associations was reported between the incidence of LDH with radiculopathy and sedentary or sitting work [1, 43]; working in a kneeling or squatting position [43]; occupational truck driving, working with hands above shoulders, or manual materials handling [43]. One study reported an association of LDH with radiculopathy and physical workload in Finnish women, but no association was found among men in the general Finnish population nor in Finnish forestry industry workers [43].

**Moderate risk of bias studies (N=13):** The evidence varied. Seven studies suggested a higher risk of LDH with radiculopathy associated with high cumulative work hours of extreme forward bending ^68^; occupational lifting and twisting with knees not bent [28, 29]; and, occupational driving and truck driving [26, 27, 30–32, 35]. Phase III evidence reported a positive dose-response relationship between cumulative lumbar load (in Newton-hours [Nh]) through manual materials handling (lifting, carrying, pulling, pushing, throwing, shoveling loads weighing at least 5 kg) and/or by intensive-load working postures (postures with trunk inclination ≥20-degrees) and LDH with radiculopathy in both men (0 to <5.0*10^6^ Nh, referent; 5.0 to <21.51*10^6^, OR 1.9 [1.1-3.0]; ≥21.51*10^6^, OR 3.7 [2.3-6.0]), and women (0 Nh, referent; >0 to <4.04*10^6^, OR 2.2 [1.3-3.8]; 4.04 to <14.47*10^6^, OR 3.6 [2.1-6.1]; ≥14.47*10^6^, OR 3.5 [2.0-5.9]OR 3.5 [2.0-5.9]) [2, 53]. Five studies found no association of LDH with radiculopathy with workplace exposure to draft or cold [50]; bending forward and backward [35]; inconvenient work postures [36]; sedentary and sitting work [35, 36]; video display terminal work [36]; working in a kneeling or squatting position [35]; twisted or bent occupational postures [50], prolonged standing at work [35]; frequency of bending and twisting; and manual materials handling in women and men [26, 27, 35, 36, 54]. Four studies indicated a tendency for a positive association with exposure to whole body vibration [2, 22, 40, 54], while two studies found no association [21, 50]. Three studies suggested a positive association of LDH with radiculopathy and work physical strenuousness and physical workload in German men [54], Han Chinese patients [21], and male workers in Denmark [55]; while two studies reported no association in Finnish populations [17, 36].

***Occupational psychosocial factors***

**Low risk of bias studies (N=1):** In their phase II study, Miranda et al. found no evidence of associations between the incidence of LDH with radiculopathy and job satisfaction, work psychosocial overload, and perceived risk of work injury [43].

**Moderate risk of bias studies (N=5):** Evidence indicated a positive association between the risk for LDH with radiculopathy and a greater number of work years with a high degree of time pressure ^68^; higher levels of perceived work injury risk, and a 3-shift work schedule in women ^39^. Job control was found to be negatively associated with the risk of LDH among the Finnish workforce [36]. No evidence of associations was reported between LDH with radiculopathy and social demands for work, mental stress at work, high work pace, problems or dissatisfaction with workmates and superiors, psychic strain through contact with clients, too much responsibility, and monotonous and boring work [36, 50, 54, 55].

**References**

1. Balling M, Holmberg T, Petersen CB, Aadahl M, Meyrowitsch DW, Tolstrup JS. Total sitting time, leisure time physical activity and risk of hospitalization due to low back pain: The Danish Health Examination Survey cohort 2007–2008. *Scand J Public Health*. 2019;47:45-52.

2. Bergmann A, Bolm-Audorff U, Ditchen D, Ellegast R, Grifka J, Haerting J, Hofmann F, Jäger M, Linhardt O, Luttmann A, Meisel HJ, Michaelis M, Petereit-Haack G, Schumann B, Seidler A. Do occupational risks for low back pain differ from risks for specific lumbar disc diseases?: Results of the German Lumbar Spine Study (EPILIFT). *Spine*. 2017;42:E1204-E1211.

3. Brauer C, Mikkelsen S, Pedersen EB, Møller KL, Simonsen EB, Koblauch H, Alkjær T, Helweg-Larsen K, Thygesen LC. Occupational lifting predicts hospital admission due to low back pain in a cohort of airport baggage handlers. *Int Arch Occup Environ Health* . 2020;93:111-122.

4. Cong L, Pang H, Xuan D, Tu GJ. Association between the expression of aggrecan and the distribution of aggrecan gene variable number of tandem repeats with symptomatic lumbar disc herniation in Chinese Han of Northern China. *Spine*. 2010;35:1371-1376.

5. Dong Q, Ren G, Zhang K, Liu D, Dou Q, Hao D. Genetic polymorphisms of ALDH2 are associated with lumbar disc herniation in a Chinese Han population. *Sci Rep*. 2018;8:13079.

6. Fidan F, Balaban M, Hatipoğlu ŞC, Veizi E. Is lumbosacral transitional vertebra associated with lumbar disc herniation in patients with low back pain? *Eur Spine J*. 2022;31:2907-2912.

7. Ghandhari H, Ameri E, Hasani H, Safari MB, Tabrizi A. Is facet tropism associated with increased risk of disc herniation in the lumbar spine? *Asian Spine J*. 2018;12:428-433.

8. Han P, Jiang F, Zhang L. The role of ADAMTS6 and ADAMTS17 polymorphisms in susceptibility to lumbar disc herniation in Chinese Han population. *Eur Spine J*. 2023;32:1106-1114.

9. Heliövaara M. Occupation and risk of herniated lumbar intervertebral disc or sciatica leading to hospitalization. *J Chronic Dis*. 1987;40:259-264.

10. Heliovaara M. Body height, obesity, and risk of herniated lumbar intervertebral disc. *Spine*. 1987;12:469-472.

11. Heliövaara M, Knekt P, Aromaa A. Incidence and risk factors of herniated lumbar intervertebral disc or sciatica leading to hospitalization. *J Chronic Dis*. 1987;40:251-258.

12. Hincapié CA, Tomlinson GA, Côté P, Rampersaud YR, Jadad AR, Cassidy JD. Chiropractic care and risk for acute lumbar disc herniation: a population-based self-controlled case series study. *Eur Spine J*. 2018;27:1526-1537.

13. Hirose Y, Chiba K, Karasugi T, Nakajima M, Kawaguchi Y, Mikami Y, Furuichi T, Mio F, Miyake A, Miyamoto T, Ozaki K, Takahashi A, Mizuta H, Kubo T, Kimura T, Tanaka T, Toyama Y, Ikegawa S. A functional polymorphism in THBS2 that affects alternative splicing and MMP binding is associated with lumbar-disc herniation. *J Med Genet*. 2008;82:1122-1129.

14. Hu B, Xing W, Li F, Huang Z, Zheng W, Ji D, Niu F, Zhu Y, Yang X. Association of glypican‐6 polymorphisms with lumbar disk herniation risk in the Han Chinese population. *Mol Genet Genomic Med*. 2019;7.

15. Hu X, Hao D, Yin J, Gong F, Wang X, Wang R, Liu B. Association between MIR31HG polymorphisms and the risk of lumbar disc herniation in Chinese Han population. *Cell Cycle*. 2022;21:2109-2120.

16. Huang X, Chen F, Zhao J, Wang D, Jing S, Li H, Meng C. Interleukin 6 (IL-6) and IL-10 promoter region polymorphisms are associated with risk of lumbar disc herniation in a northern Chinese Han population. *Genet Test Mol Biomarkers*. 2017;21:17-23.

17. Hurme M, Alaranta H, Törmä T, Einola S. Operated lumbar disc herniation: epidemiological aspects. *Ann Chir Gynaecol* . 1983;72:33-6.

18. Jarvik JG, Hollingworth W, Heagerty PJ, Haynor DR, Boyko EJ, Deyo RA. Three-year incidence of low back pain in an initially asymptomatic cohort. *Spine*. 2005;30:1541-1548.

19. Jhawar BS, Fuchs CS, Colditz GA, Stampfer MJ. Cardiovascular risk factors for physician-diagnosed lumbar disc herniation. *Spine J*. 2006;6:684-691.

20. Ji D, Xing W, Li F, Huang Z, Zheng W, Hu B, Niu F, Zhu Y, Yang X. Correlation of EYS polymorphisms with lumbar disc herniation risk among Han Chinese population. *Mol Genet Genomic Med*. 2019;7.

21. Jiang H, Yang Q, Jiang J, Zhan X, Xiao Z. Association between COL11A1 (rs1337185) and ADAMTS5 (rs162509) gene polymorphisms and lumbar spine pathologies in Chinese Han population: an observational study. *BMJ Open*. 2017;7:e015644.

22. Jing R, Liu Y, Guo P, Ni T, Gao X, Mei R, He X, Zhang J. Evaluation of common variants in matrix metalloproteinase-9 gene with lumbar disc herniation in Han Chinese population. *Genet Test Mol Biomarkers*. 2018;22:622-629.

23. Jørgensen MB, Holtermann A, Gyntelberg F, Suadicani P. Physical fitness as a predictor of herniated lumbar disc disease – a 33-year follow-up in the Copenhagen male study. *BMC Musculoskelet Disord*. 2013;14:86.

24. Jung J-M, Lee SU, Hyun S-J, Kim K-J, Jahng T-A, Oh CW, Kim H-J. Trends in incidence and treatment of herniated lumbar disc in Republic of Korea : A nationwide database study. *J Korean Neurosurg Soc* . 2020;63:108-118.

25. Karasugi T, Semba K, Hirose Y, Kelempisioti A, Nakajima M, Miyake A, Furuichi T, Kawaguchi Y, Mikami Y, Chiba K, Kamata M, Ozaki K, Takahashi A, Mäkelä P, Karppinen J, Kimura T, Kubo T, Toyama Y, Yamamura K, Männikkö M, Mizuta H, Ikegawa S. Association of the tag SNPs in the human SKT gene (KIAA1217) with lumbar disc herniation. *J Bone Miner Res*. 2009;24:1537-1543.

26. Kelsey JL. An epidemiological study of the relationship between occupations and acute herniated lumbar intervertebral discs. *Int J Epidemiol*. 1975;4:197-205.

27. Kelsey JL. An epidemiological study of acute herniated lumbar intervertebral discs. *Rheumatol Rehabil*. 1975;14:144-59.

28. Kelsey JL, Githens PB, OʼConner T, Weil U, Calogero JA, Hohlford TM, White AA, Walter SD, Ostfeld AM, Southwick WO. Acute prolapsed lumbar intervertebral disc. An epidemiologic study with special reference to driving automobiles and cigarette smoking. *Spine*. 1984;9:608-613.

29. Kelsey JL, Githens PB, White AA, Holford TR, Walter SD, O’Connor T, Ostfeld AM, Weil U, Southwick WO, Calogero JA. An epidemiologic study of lifting and twisting on the job and risk for acute prolapsed lumbar intervertebral disc. *J Orthop Res*. 1984;2:61-66.

30. Kelsey JL, Greenberg RA, Hardy RJ, Johnson MF. Pregnancy and the syndrome of herniated lumbar intervertebral disc; an epidemiological study. *Yale J Biol Med*. 1975;48:361-8.

31. Kelsey JL, Hardy RJ. Driving of motor vehicles as a risk factor for acute herniated lumbar intervertebral disc. *Am J Epidemiol*. 1975;102:63-73.

32. Kelsey JL, Ostfeld AM. Demographic characteristics of persons with acute herniated lumbar intervertebral disc. *J Chronic Dis*. 1975;28:37-50.

33. Kim Y-K, Kang D, Lee I, Kim S-Y. Differences in the incidence of symptomatic cervical and lumbar disc herniation according to age, sex and national health insurance eligibility: A pilot study on the disease’s association with work. *Int J Environ Res Public Health*. 2018;15:2094.

34. Knox JB, Deal JB, Knox JA. Lumbar disc herniation in military helicopter pilots vs. matched controls. *Aerosp Med Hum Perform*. 2018;89:442-445.

35. Leclerc A. Personal and occupational predictors of sciatica in the GAZEL cohort. *Occup Med*. 2003;53:384-391.

36. Leino-Arjas P, Kaila-Kangas L, Kauppinen T, Notkola V, Keskimäki I, Mutanen P. Occupational exposures and inpatient hospital care for lumbar intervertebral disc disorders among Finns. *Am J Ind Med*. 2004;46:513-520.

37. Leino-Arjas P, Kaila-Kangas L, Keskimäki I, Notkola V, Mutanen P. Inpatient hospital care for lumbar intervertebral disc disorders in Finland in relation to education, occupational class, income, and employment. *Public Health*. 2002;116:272-278.

38. Li L, Ni D, Zhu F. No association between VDR gene polymorphisms and lumbar disc herniation in a Chinese population. *Int J Clin Exp Med*. 2018.

39. Liu K, Huo H, Jia W, Li M, Xiong Z, Sun Y, Wu J, Li H, Liu J, Liu Y, Jin T, Li B, Zuo Y, Zhao Y. RAB40C gene polymorphisms rs62030917 and rs2269556 are associated with an increased risk of lumbar disc herniation development in the Chinese Han population. *J Gene Med*. 2021;23.

40. Luo Y, Wang J, Pei J, Rong Y, Liu W, Tang P, Cai W, Yin G. Interactions between the MMP‐3 gene rs591058 polymorphism and occupational risk factors contribute to the increased risk for lumbar disk herniation: A case‐control study. *J Clin Lab Anal*. 2020;34.

41. Mattila VM, Saarni L, Parkkari J, Koivusilta L, Rimpelä A. Early risk factors for lumbar discectomy: an 11-year follow-up of 57,408 adolescents. *Eur Spine J* . 2008;17:1317-1323.

42. Mio F, Chiba K, Hirose Y, Kawaguchi Y, Mikami Y, Oya T, Mori M, Kamata M, Matsumoto M, Ozaki K, Tanaka T, Takahashi A, Kubo T, Kimura T, Toyama Y, Ikegawa S. A functional polymorphism in COL11A1, which encodes the α1 chain of type XI collagen, is associated with susceptibility to lumbar disc herniation. *Am J Hum Genet*. 2007;81:1271-1277.

43. Miranda H, Viikari-Juntura E, Martikainen R, Takala E-P, Riihimäki H. Individual factors, occupational loading, and physical exercise as predictors of sciatic pain. *Spine*. 2002;27:1102-9.

44. Mu J, Ge W, Zuo X, Chen Y, Huang C. A SNP in the 5′UTR of GDF5 is associated with susceptibility to symptomatic lumbar disc herniation in the Chinese Han population. *Eur Spine J*. 2014;23:498-503.

45. Mundt DJ, Kelsey JL, Golden AL, Panjabi MM, Pastides H, Berg AT, Sklar J, Hosea T. An epidemiologic study of sports and weight lifting as possible risk factors for herniated lumbar and cervical discs. *Am J Sports Med* . 1993;21:854-860.

46. Mundt DJ, Kelsey JL, Golden AL, Pastides H, Berg AT, Sklar J, Hosea T, Panjabi MM. An epidemiologic study of non-occupational lifting as a risk factor for herniated lumbar intervertebral disc. *Spine*. 1993;18:595-602.

47. Noponen-Hietala N, Virtanen I, Karttunen R, Schwenke S, Jakkula E, Li H, Merikivi R, Barral S, Ott J, Karppinen J, Ala-Kokko L. Genetic variations in IL6 associate with intervertebral disc disease characterized by sciatica. *Pain*. 2005;114:186-194.

48. Nyrhi L, Kuitunen I, Ponkilainen V, Mäntymäki H, Huttunen TT, Mattila VM. Incidence of lumbar discectomy during pregnancy and within 12 months post-partum in Finland between 1999 and 2017: a retrospective register-based cohort study. *Spine J*. 2023;23:287-294.

49. Pietri-Taleb F, Riihimäki H, Viikari-Juntura E, Lindström K, Moneta GB. The role of psychological distress and personality in the incidence of sciatic pain among working men. *Am J Public Health*. 1995;85:541-545.

50. Riihimäki H, Viikari-Juntura E, Moneta G, Kuha J, Videman T, Tola S. Incidence of sciatic pain among men in machine operating, dynamic physical work, and sedentary work. *Spine*. 1994;19:138-142.

51. Riihimaki H, Wickström G, Hanninen K, Luopajarvi T. Predictors of sciatic pain among concrete reinforcement workers and house painters--a five-year follow-up. *Scand J Work Environ Health*. 1989;15:415-423.

52. Schumann B, Bolm-Audorff U, Bergmann A, Ellegast R, Elsner G, Grifka J, Haerting J, Jäger M, Michaelis M, Seidler A. Lifestyle factors and lumbar disc disease: results of a German multi-center case-control study (EPILIFT). *Arthritis Res Ter*. 2010;12:R193.

53. Seidler A, Bergmann A, Jäger M, Ellegast R, Ditchen D, Elsner G, Grifka J, Haerting J, Hofmann F, Linhardt O, Luttmann A, Michaelis M, Petereit-Haack G, Schumann B, Bolm-Audorff U. Cumulative occupational lumbar load and lumbar disc disease – results of a German multi-center case-control study (EPILIFT). *BMC Musculoskelet Disord*. 2009;10:48.

54. Seidler A, Bolm-Audorff U, Siol T, Henkel N, Fuchs C, Schug H, Leheta F, Marquardt G, Schmitt E, Ulrich P, Beck W, Missalla A, Elsner G. Occupational risk factors for symptomatic lumbar disc herniation; a case-control study. *Occup Environ Med* . 2003;60:821-830.

55. Sørensen IG, Jacobsen P, Gyntelberg F, Suadicani P. Occupational and other predictors of herniated lumbar disc disease—A 33-year follow-up in the Copenhagen Male Study. *Spine*. 2011;36:1541-1546.

56. Suri P, Boyko EJ, Goldberg J, Forsberg CW, Jarvik JG. Longitudinal associations between incident lumbar spine MRI findings and chronic low back pain or radicular symptoms: retrospective analysis of data from the longitudinal assessment of imaging and disability of the back (LAIDBACK). *BMC Musculoskelet Disord*. 2014;15:152.

57. Virtanen IM, Song YQ, Cheung KMC, Ala-Kokko L, Karppinen J, Ho DWH, Luk KDK, Yip SP, Leong JCY, Cheah KSE, Sham P, Chan D. Phenotypic and population differences in the association between CILP and lumbar disc disease. *J Med Genet*. 2007;44:285-288.

58. Wahlström J, Burström L, Johnson PW, Nilsson T, Järvholm B. Exposure to whole-body vibration and hospitalization due to lumbar disc herniation. *Int Arch Occup Environ Health*. 2018;91:689-694.

59. Wahlström J, Burström L, Nilsson T, Järvholm B. Risk factors for hospitalization due to lumbar disc disease. *Spine*. 2012;37:1334-1339.

60. Wang Y, Wang H, Lv F, Ma X, Xia X, Jiang J. Asymmetry between the superior and inferior endplates is a risk factor for lumbar disc degeneration. *J Orthop Res*. 2018;36:2469-2475.

61. Withanage ND, Perera S, Peiris H, Athiththan LV. Serum 25-hydroxyvitamin D, serum calcium and vitamin D receptor (VDR) polymorphisms in a selected population with lumbar disc herniation—A case control study. *PLoS One*. 2018;13:e0205841.

62. Wu J, Sun Y, Xiong Z, Liu J, Li H, Liu Y, Li B, Jin T. Association of GSDMC polymorphisms with lumbar disc herniation among Chinese Han population. *Int J Immunogenet*. 2020;47:546-553.

63. Wu Y, Bai M, Yu Y, Wang Y, Zhang Y. Association of LINC-PINT polymorphisms with lumbar disc herniation risk among Chinese Han population: a case control study. *J Orthop Surg Res*. 2023;18:585.

64. Yang X, Guo X, Huang Z, Da Y, Xing W, Li F, Li M, Sun K, Jia H, Zhu Y. CHRNA5/CHRNA3 gene cluster is a risk factor for lumbar disc herniation: a case-control study. *J Orthop Surg Res*. 2019;14:243.

65. Zhou Q, Teng D, Zhang T, Lei X, Jiang W. Association of facet tropism and orientation with lumbar disc herniation in young patients. *Neurol Sci*. 2018;39:841-846.

66. Zhu Y, Jia H, Li J, Ren S, Huang Z, Li F, Xing W, Li S, Yang X. Associations between variants in BDNF/BDNFOS gene and lumbar disc herniation risk among Han Chinese people. *Sci Rep*. 2018;8:12782.

67. Zitting P, Rantakallio P, Vanharanta H. Cumulative incidence of lumbar disc diseases leading to hospitalization up to the age of 28 years. *Spine*. 1998;23:2337-43; discussion 2343-4.
