# Supplementary material for: Incidence of and risk factors for lumbar disc herniation with radiculopathy in adults: a systematic review
Source: Eur Spine J. 2024 Oct 25;34(1):263–94. doi: 10.1007/s00586-024-08528-8 (PMC11754359; doi:10.1007/s00586-024-08528-8)
Supplement: Supplementary file 3 — Supplementary file3 (DOCX 177 KB) [file 586_2024_8528_MOESM3_ESM.docx]

Excluded articles after full-text screen and the primary reason for exclusion (sorted by reason for exclusion, chronologically, and author [alphabetically]).

| Exclusion reason: Ineligible outcome of interest (N=41) |
| --- |
| 1. Zawilla NH, Darweesh H, Mansour N, et al. Matrix metalloproteinase-3, vitamin D receptor gene polymorphisms, and occupational risk factors in lumbar disc degeneration. J Occup Rehabil. 2014;24(2):370-381. doi:10.1007/s10926-013-9472-7 2. Duran S, Cavusoglu M, Hatipoglu HG, Sozmen Cılız D, Sakman B. Association between measures of Vertebral Endplate Morphology and Lumbar Intervertebral Disc Degeneration. *Canadian Association of Radiologists Journal*. 2017;68(2):210-216. doi:10.1016/j.carj.2016.11.002 3. Vinas-Rios JM, Sanchez-Aguilar M, Medina-Govea FA, Meyer F. Early recurrent lumbar disc herniation-data from the German spine registry. European Spine Journal. 2017;26(11):2978-3057. doi:10.1007/s00586-017-5336-8 4. Zhu Y, Li S, Niu F, et al. Association between IL4, IL6 gene polymorphism and lumbar disc degeneration in Chinese population. Oncotarget. 2017;8(51):89064-89071. doi:10.18632/oncotarget.21650 5. Chen Y, Ma H, Bi D, Qiu B. Association of interleukin 1 gene polymorphism with intervertebral disc degeneration risk in the Chinese Han population. Biosci Rep. 2018;38(4). doi:10.1042/BSR20171627 6. Huang WC, Kuo CH, Wu JC, Chen YC. Higher risk of intervertebral disc herniation among neurosurgeons than neurologists: 15 year-follow-up of a physician cohort. J Clin Med. 2018;7(8):198. doi:10.3390/jcm7080198 7. Kitis S, Coskun ZM, Tasdemir P, Tuncez E, Zamani AG, Acar A. Analysis of genetic polymorphisms associated with intervertebral disc degeneration. Cell Mol Biol (Noisy-le-grand). 2018;64(10):61-65. 8. Korshøj M, Jørgensen MB, Hallman DM, Lagersted-Olsen J, Holtermann A, Gupta N. Prolonged sitting at work is associated with a favorable time course of low-back pain among blue-collar workers: a prospective study in the DPhacto cohort. Scand J Work Environ Health. 2018;44(5):530-538. doi:10.5271/sjweh.3726 9. Zhou X, Cheung CL, Karasugi T, et al. Trans-ethnic polygenic analysis supports genetic overlaps of lumbar disc degeneration with height, body mass index, and bone mineral density. Front Genet. 2018;9. doi:10.3389/fgene.2018.00267 10. Schistad EI, Bjorland S, Røe C, et al. Five-year development of lumbar disc degeneration—a prospective study. Skeletal Radiol. 2019;48(6):871-879. doi:10.1007/s00256-018-3062-x 11. Akarirmak U, Sari H. Lumbar disc herniation and vitamin d receptor gene polymorphisms in Turkish patients. Osteoporosis International. 2020;31(S1):133-621. doi:10.1007/s00198-020-05696-3 12. Ding Y, Lv S, Li G, Dong S, Sun X, Chen Y. Scheuermann’s disease as a risk factor for lumbar disc herniation recurrence. J Coll Physicians Surg Pak. 2020;30(06):584-589. doi:10.29271/jcpsp.2020.06.584 13. Hung IYJ, Shih TTF, Chen BB, Liou SH, Ho IK, Guo YL. The roles of lumbar load thresholds in cumulative lifting exposure to predict disk protrusion in an Asian population. BMC Musculoskelet Disord. 2020;21(1):169. doi:10.1186/s12891-020-3167-y 14. Kiraz M, Demir E. Relationship of lumbar disc degeneration with hemoglobin value and smoking. Neurochirurgie. 2020;66(5):373-377. doi:10.1016/j.neuchi.2020.06.133 15. Öz T, Kaya İ, Nursal AF, Aydın HE, Demir O, Yiğit S. ACAN gene VNTR polymorphism and intervertebral disc degeneration in a Turkish population. Medical Bulletin of Haseki. 2020;58(4):309-314. doi:10.4274/haseki.galenos.2020.6006 16. Wirries N, Schwarze M, Daentzer D, Skutek M. Total hip arthroplasty and lumbar spine disorders: Plain co-existence or mutual influence? Orthop Rev (Pavia). 2020;12(2). doi:10.4081/or.2020.8546 17. Baker HP, Mosenthal W, Qin C, Volchenko E, Athiviraham A. Is average club head speed a risk factor for lower back injuries in professional golfers? A retrospective case control study. Phys Sportsmed. 2021;49(2):214-218. doi:10.1080/00913847.2020.1809968 18. Abdallah A, Emel E, Güler Abdallah B. Factors associated with the recurrence of lumbar disk herniation: biomechanical–radiological and demographic factors. Neurol Res. 2022;44(9):830-846. doi:10.1080/01616412.2022.2056340 19. Choi TY, Chang MY, Lee SH, Cho JG, Lee S. Psoas muscle measurement as a predictor of recurrent lumbar disc herniation: A retrospective blind study. Medicine. 2022;101(26):e29778. doi:10.1097/MD.0000000000029778 20. Guo J, Li G, Ji X, et al. Clinical and radiological risk factors of early recurrent lumbar disc herniation at six months or less: a clinical retrospective analysis in one medical center. Pain Physician. 2022;25(7):E1039-E1045. 21. Iachina M, Ljungdalh P, Nørgård BM, Garvik O, Stenager E, Schiøttz-Christensen B. Psychiatric disorders, diagnosed in psychiatric clinics, in patients with back pain: A cohort study. Scand J Public Health. 2023;51(8):1153-1160. doi:10.1177/14034948221100105 22. Kao YC, Chen JY, Chen HH, Liao KW, Huang SS. The association between depression and chronic lower back pain from disc degeneration and herniation of the lumbar spine. The International Journal of Psychiatry in Medicine. 2022;57(2):165-177. doi:10.1177/00912174211003760 23. Kasch R, Truthmann J, Hancock MJ, et al. Association of lumbar MRI findings with current and future back pain in a population-based cohort study. Spine (Phila Pa 1976). 2022;47(3):201-211. doi:10.1097/BRS.0000000000004198 24. Konovalov NA, Nazarenko AG, Brinyuk ES, Kaprovoy SV, Beloborodov VA, Stepanov IA. Risk factors for recurrent lumbar disk herniation. Coluna/Columna. 2022;21(4). doi:10.1590/s1808-185120222104263325 25. Ono K, Ohmori K, Yoneyama R, Matsushige O, Majima T. Risk factors and surgical management of recurrent herniation after full-endoscopic 26. Siccoli A, Staartjes VE, Klukowska AM, Muizelaar JP, Schröder ML. Overweight and smoking promote recurrent lumbar disk herniation after discectomy. European Spine Journal. 2022;31(3):604-613. doi:10.1007/s00586-022-07116-y 27. Thakar S, Raj V, Neelakantan S, et al. Spinal morphometry as a novel predictor for recurrent lumbar disc herniation requiring revision surgery: results of a case control study. Neurol India. 2022;70(8):211. doi:10.4103/0028-3886.360932 28. Wang F, Chen K, Lin Q, et al. Earlier or heavier spinal loading is more likely to lead to recurrent lumbar disc herniation after percutaneous endoscopic lumbar discectomy. J Orthop Surg Res. 2022;17(1):356. doi:10.1186/s13018-022-03242-x 29. Wang X, Liu H, Wang W, et al. Comparison of multifidus degeneration between scoliosis and lumbar disc herniation. BMC Musculoskelet Disord. 2022;23(1):891. doi:10.1186/s12891-022-05841-5 30. Abdallah A, Güler Abdallah B. Factors associated with the recurrence of lumbar disk herniation: non-biomechanical–radiological and intraoperative factors. Neurol Res. 2023;45(1):11-27. doi:10.1080/01616412.2022.2116525 31. Borja AJ, Connolly J, Kvint S, et al. Household income is associated with return to surgery following discectomy for far lateral disc herniation. J Neurosurg Sci. 2023;67(3). doi:10.23736/S0390-5616.21.05246-2 32. Geere JH, Swamy GN, Hunter PR, et al. Incidence and risk factors for five-year recurrent disc herniation after primary single-level lumbar discectomy. Bone Joint J. 2023;105-B(3):315-322. doi:10.1302/0301-620X.105B3.BJJ-2022-1005.R2 33. He H, Ma J, Xiong C, et al. Development and validation of a nomogram to predict the risk of lumbar disk reherniation within 2 years after percutaneous endoscopic lumbar discectomy. World Neurosurg. 2023;172:e349-e356. doi:10.1016/j.wneu.2023.01.026 34. Kızılgöz V, Aydın S, Karavaş E, Kantarcı M, Kahraman Ş. Are paraspinal muscle quantity, lumbar indentation value, and subcutaneous fat thickness related to disc degeneration? An MRI-based study. Radiography. 2023;29(2):428-435. doi:10.1016/j.radi.2023.02.004 35. Lastra-Power J, Nieves-Ríos C, Baralt-Nazario F, et al. Predictors of reoperation in hispanic-americans with recurrent lumbosacral disc herniation following primary hemilaminectomy and discectomy surgery. World Neurosurg X. 2023;18:100172. doi:10.1016/j.wnsx.2023.100172 36. Li X, Pan B, Cheng L, Li G, Liu J, Yuan F. Development and validation of a prognostic model for the risk of recurrent lumbar disc herniation after percutaneous endoscopic transforaminal discectomy. Pain Physician. 2023;26(1):81-90. 37. Mäntymäki H, Ponkilainen VT, Huttunen TT, Mattila VM. Regional variations in lumbar spine surgery in Finland. Arch Orthop Trauma Surg. 2021;143(3):1451-1458. doi:10.1007/s00402-021-04313-0 38. Monticelli M, Gelmi CAE, Scerrati A, Cavallo MA, De Bonis P. Recurrent or junctional lumbar foraminal herniated disc in patients operated with trans pars microscopic approach. Neurosurg Rev. 2023;46(1):211. doi:10.1007/s10143-023-02109-x 39. Shan ZM, Ren XS, Shi H, et al. Machine learning prediction model and risk factor analysis of reoperation in recurrent lumbar disc herniation patients after percutaneous endoscopic lumbar discectomy. Global Spine J. Published online May 10, 2023:219256822311733. doi:10.1177/21925682231173353 40. Zhong D, Wang Y, Lin L, et al. Development and validation of a nomogram to predict the risk of recurrent lower extremity radiating pain within 1 week following full-endoscopic lumbar discectomy. World Neurosurg. 2023;179:e348-e358. doi:10.1016/j.wneu.2023.08.090 41. Zhu F, Jia D, Zhang Y, et al. Moderate to severe multifidus fatty atrophy is the risk factor for recurrence after microdiscectomy of lumbar disc herniation. Neurospine. 2023;20(2):637-650. doi:10.14245/ns.2346054.027 |
| Exclusion reason: Ineligible design (N=23) |
| 1. Chadha M, Sharma G, Arora SS, Kochar V. Association of facet tropism with lumbar disc herniation. European Spine Journal. 2013;22(5):1045-1052. doi:10.1007/s00586-012-2612-5 2. Pan J, Lu X, Yang G, Han Y, Tong X, Wang Y. Lumbar disc degeneration was not related to spine and hip bone mineral densities in Chinese: facet joint osteoarthritis may confound the association. Arch Osteoporos. 2017;12(1):20. doi:10.1007/s11657-017-0315-6 3. Papic M, Papic V, Kresoja M, Munteanu V, Mikov I, Cigic T. Relation between grades of intervertebral disc degeneration and occupational activities of patients with lumbar disc herniation. Vojnosanit Pregl. 2017;74(12):1121-1127. doi:10.2298/VSP151112306P 4. Shahlaee A, Rahimi-Movaghar V. A familial incidence of L1-L2 disc herniation. J Neurosurg Sci. 2016;61(2). doi:10.23736/S0390-5616.16.03262-8 5. Ravindra VM, Senglaub SS, Rattani A, et al. Degenerative lumbar spine disease: estimating global incidence and worldwide volume. Global Spine J. 2018;8(8):784-794. doi:10.1177/2192568218770769 6. Reito A, Kyrölä K, Pekkanen L, Paloneva J. Specific spinal pathologies in adult patients with an acute or subacute atraumatic low back pain in the emergency department. Int Orthop. 2018;42(12):2843-2849. doi:10.1007/s00264-018-3983-y 7. Läubli R, Brugger R, Pirvu T, et al. Disproportionate vertebral bodies and their impact on lumbar disc herniation. J Clin Med. 2021;10(14):3174. doi:10.3390/jcm10143174 8. Zehra U, Cheung JPY, Bow C, et al. Spinopelvic alignment predicts disc calcification, displacement, and Modic changes: Evidence of an evolutionary etiology for clinically‐relevant spinal phenotypes. JOR Spine. 2020;3(1). doi:10.1002/jsp2.1083 9. Walter SS, Lorbeer R, Hefferman G, et al. Correlation between thoracolumbar disc degeneration and anatomical spinopelvic parameters in supine position on MRI. PLoS One. 2021;16(6):e0252385. doi:10.1371/journal.pone.0252385 10. Cha E woo, Jung S mi, Lee I ho, et al. Approval status and characteristics of work-related musculoskeletal disorders among Korean workers in 2020. Ann Occup Environ Med. 2022;34(1). doi:10.35371/aoem.2022.34.e31 11. Dittmar-Johnson HM, Cruz-López F, González-Camacho E, et al. Prevalence and characteristics of upper lumbar disc herniations in our practice: a retrospective analysis. Coluna/Columna. 2022;21(1). doi:10.1590/s1808-185120222101259474 12. Pourabbas Tahvildari B, Masroori Z, Erfani MA, Solooki S, Vosoughi AR. The impact of spino-pelvic parameters on pathogenesis of lumbar disc herniation. Musculoskelet Surg. 2022;106(2):195-199. doi:10.1007/s12306-020-00693-5 13. Raymaekers V, Bamps S, Duyvendak W, et al. Real world data collection and cluster analysis in patients with sciatica due to lumbar disc herniation. Clin Neurol Neurosurg. 2022;217:107246. doi:10.1016/j.clineuro.2022.107246 14. Azemi ES, Kola S, Kola I, Tanka M, Bilaj F, Abazaj E. Lumbar disk herniation: a clinical epidemiological and radiological evaluation. Open Access Maced J Med Sci. 2022;10(B):1588-1594. doi:10.3889/oamjms.2022.8828 15. Tarabeih N, Shalata A, Higla O, Kalinkovich A, Livshits G. The search for systemic biomarkers for monitoring degenerative lumbar spinal disorders. Osteoarthr Cartil Open. 2022;4(4):100323. doi:10.1016/j.ocarto.2022.100323 16. Wang P, Chen C, Liu F, et al. The effects of ambient temperature on lumbar disc herniation: a retrospective study. Front Med (Lausanne). 2022;9. doi:10.3389/fmed.2022.811237 17. Yazici A, Yerlikaya T. The relationship between the degeneration and asymmetry of the lumbar multifidus and erector spinae muscles in patients with lumbar disc herniation with and without root compression. J Orthop Surg Res. 2022;17(1):541. doi:10.1186/s13018-022-03444-3 18. Zhao X, Liang H, Hua Z, et al. The morphological characteristics of paraspinal muscles in young patients with unilateral neurological symptoms of lumbar disc herniation. BMC Musculoskelet Disord. 2022;23(1):994. doi:10.1186/s12891-022-05968-5 19. Chen X, Li Y, Wang W, Cui P, Wang Y, Lu S. Correlation between inflammatory cytokine expression in paraspinal tissues and severity of disc degeneration in individuals with lumbar disc herniation. BMC Musculoskelet Disord. 2023;24(1):193. doi:10.1186/s12891-023-06295-z 20. Ke S, Sun T, Zhang W, Zhang J, Li Z. Are there correlations between facet joint parameters and lumbar disk herniation laterality in young adults? Journal of Clinical Neuroscience. 2023;109:50-56. doi:10.1016/j.jocn.2023.01.013 21. Ordaz A, Anderson B, Zlomislic V, et al. Paraspinal muscle gene expression across different aetiologies in individuals undergoing surgery for lumbar spine pathology. European Spine Journal. 2023;32(4):1123-1131. doi:10.1007/s00586-023-07543-5 22. Takahashi M, Iwamoto K, Tomita K, Ueda S, Igawa T, Miyauchi Y. Factors associated with spinal instability in low back lumbar diseases with leg pain: Analysis of sagittal translation and segmental angulation. J Back Musculoskelet Rehabil. 2023;36(2):437-444. doi:10.3233/BMR-220067 23. Tian G, Wang Y, Xia J, et al. Correlation of multifidus degeneration with sex, age and side of herniation in patients with lumbar disc herniation. BMC Musculoskelet Disord. 2023;24(1):652. doi:10.1186/s12891-023-06783-2 |
| Exclusion reason: Ineligible population (N=7) |
| 1. Rajasekaran S, Kanna RM, Reddy RR, et al. How reliable are the reported genetic associations in disc degeneration? Spine (Phila Pa 1976). 2016;41(21):1649-1660. doi:10.1097/BRS.0000000000001847 2. Chiu CD, Chen HJ, Saw HP, Yao NW, Yen HR, Kao CH. Asthma and early herniated intervertebral disc disease. Curr Med Res Opin. 2017;33(11):2019-2025. doi:10.1080/03007995.2017.1330260 3. Botti B, Rodriguez J, Mehallo C, Simon J, Close J, Rhoden I. Incidence of causes of adolescent back pain. Clinical Journal of Sport Medicine. 2019;29(2):154-161. doi:10.1097/JSM.0000000000000726 4. Aydin HE, Yigit S, Kaya I, Tural E, Tuncer S, Nursal AF. VEGF and eNOS variants may influence intervertebral disc degeneration. Nucleosides Nucleotides Nucleic Acids. 2022;41(10):982-993. doi:10.1080/15257770.2022.2093363 5. Sayin Gülensoy E, Gülensoy B. A 9-year retrospective cohort of patients with lumbar disc herniation: Comparison of patient characteristics and recurrence frequency by smoking status. Medicine. 2022;101(51):e32462. doi:10.1097/MD.0000000000032462 6. Ishihama Y, Tezuka F, Manabe H, et al. Facet joint morphology and tropism in adolescents. Spine (Phila Pa 1976). Published online September 4, 2023. doi:10.1097/BRS.0000000000004818 7. Ovcharov ME, Mladenovski MN, Mladenovski IN, Valkov I V., Vasilkova SB. Lumbar disc herniation in children and elderly patients. Folia Med (Plovdiv). 2023;65(4):631-637. doi:10.3897/folmed.65.e97233 |
| Exclusion reason: Ineligible publication type (N=8) |
| 1. Doraiswamy R, Ramaswami K, Subramanian R, Srinivasan DK, Sivasankaran B. Association of Vitamin‐D receptor polymorphisms with degenerative disc disease in Indian population. The FASEB Journal. 2017;31(S1). doi:10.1096/fasebj.31.1_supplement.902.19 2. Tarnoki AD, Tárnoki DL, Szily M, et al. Relationship between obstructive sleep apnea and lumbal disc protrusion: A twin study. Twin Research and Human Genetics. 2017;20(6):564-642. doi:10.1017/thg.2017.63 3. Kim H doo, Kim DH, An YS, Jeong KS, Ahn YS, Yoon J ha. 1314 Risk assessment for back pain and lumbar degenerative disease in korean firefighters. In: Musculoskeletal Disorders. BMJ Publishing Group Ltd; 2018:A266.1-A266. doi:10.1136/oemed-2018-ICOHabstracts.760 4. Lener S, Hartmann S, Thoḿe C, Tschugg A. The impact of obesity on young individuals suffering from lumbar disc herniation: A retrospective analysis of 97 cases. Global Spine J. 2018;8(1_suppl):174S-374S. doi:10.1177/2192568218771072 5. Vidal Rodriguez S, Sánchez Benitez De Soto J. Genetic polymorphism analysis of VDR, COL1A1, GDF5, THSB2, CHST3, rank, and opg in patients with symptomatic lumbar herniated disc and surgical indication. European Spine Journal. 2018;27(10):2665-2690. doi:10.1007/s00586-018-5769-8 6. Abid M, Ullah Khan H, Huzea Abid M, et al. association of occupational risk factors with the level of lumbar disc nucleus pulposus herniation. Pakistan Journal of Medical and Health Sciences. 2021;15(10):2863-2864. doi:10.53350/pjmhs2115102863 7. Unsal D, Subasi F, Kaya AH, Gulec Yilmaz S, Yaltirik CK, Isbir T. Association of IL 1-P (rs 1143627 T/C) gene polymorphism with lumbar disc degeneration in Turkish population: a case-control study. European Spine Journal. 2021;30(11):3328-3414. doi:10.1007/s00586-021-07017-6 8. Hur J, Hong JT. Risk factor analysis for inferior clinical outcome and recurrence after full-endoscopic interlaminar discectomy (FEID) for lumbar disc herniations (LDH); A prospective observational study. Global Spine J. 2022;12(3_suppl):205S-355S. doi:10.1177/21925682221096075 |

Publication type^1–8^

Population^9–15^

Outcome of interest^16–56^

Design^57–79^

1. Doraiswamy R, Ramaswami K, Subramanian R, Srinivasan DK, Sivasankaran B. Association of Vitamin‐D receptor polymorphisms with degenerative disc disease in Indian population. *The FASEB Journal*. 2017;31(S1). doi:10.1096/fasebj.31.1_supplement.902.19

2. Tarnoki AD, Tárnoki DL, Szily M, et al. Relationship between obstructive sleep apnea and lumbal disc protrusion: A twin study. *Twin Research and Human Genetics*. 2017;20(6):564-642. doi:10.1017/thg.2017.63

3. Kim H doo, Kim DH, An YS, Jeong KS, Ahn YS, Yoon J ha. 1314 Risk assessment for back pain and lumbar degenerative disease in korean firefighters. In: *Musculoskeletal Disorders*. BMJ Publishing Group Ltd; 2018:A266.1-A266. doi:10.1136/oemed-2018-ICOHabstracts.760

4. Lener S, Hartmann S, Thoḿe C, Tschugg A. The impact of obesity on young individuals suffering from lumbar disc herniation: A retrospective analysis of 97 cases. *Global Spine J*. 2018;8(1_suppl):174S-374S. doi:10.1177/2192568218771072

5. Abid M, Ullah Khan H, Huzea Abid M, et al. Association of Occupational Risk Factors with the Level of Lumbar Disc Nucleus Pulposus Herniation. *Pakistan Journal of Medical and Health Sciences*. 2021;15(10):2863-2864. doi:10.53350/pjmhs2115102863

6. Hur J, Hong JT. Risk factor analysis for inferior clinical outcome and recurrence after full-endoscopic interlaminar discectomy (FEID) for lumbar disc herniations (LDH); A prospective observational study. *Global Spine J*. 2022;12(3_suppl):205S-355S. doi:10.1177/21925682221096075

7. Unsal D, Subasi F, Kaya AH, Gulec Yilmaz S, Yaltirik CK, Isbir T. Association of IL 1-P (rs 1143627 T/C) Gene Polymorphism with Lumbar Disc Degeneration in Turkish Population: A Case-Control Study. *European Spine Journal*. 2021;30(11):3328-3414. doi:10.1007/s00586-021-07017-6

8. Vidal Rodriguez S, Sánchez Benitez De Soto J. Genetic polymorphism analysis of VDR, COL1A1, GDF5, THSB2, CHST3, rank, and opg in patients with symptomatic lumbar herniated disc and surgical indication. *European Spine Journal*. 2018;27(10):2665-2690. doi:10.1007/s00586-018-5769-8

9. Rajasekaran S, Kanna RM, Reddy RR, et al. How reliable are the reported genetic associations in disc degeneration? *Spine (Phila Pa 1976)*. 2016;41(21):1649-1660. doi:10.1097/BRS.0000000000001847

10. Chiu CD, Chen HJ, Saw HP, Yao NW, Yen HR, Kao CH. Asthma and early herniated intervertebral disc disease. *Curr Med Res Opin*. 2017;33(11):2019-2025. doi:10.1080/03007995.2017.1330260

11. Botti B, Rodriguez J, Mehallo C, Simon J, Close J, Rhoden I. Incidence of causes of adolescent back pain. *Clinical Journal of Sport Medicine*. 2019;29(2):154-161. doi:10.1097/JSM.0000000000000726

12. Aydin HE, Yigit S, Kaya I, Tural E, Tuncer S, Nursal AF. *VEGF* and *eNOS* variants may influence intervertebral disc degeneration. *Nucleosides Nucleotides Nucleic Acids*. 2022;41(10):982-993. doi:10.1080/15257770.2022.2093363

13. Sayin Gülensoy E, Gülensoy B. A 9-year retrospective cohort of patients with lumbar disc herniation: Comparison of patient characteristics and recurrence frequency by smoking status. *Medicine*. 2022;101(51):e32462. doi:10.1097/MD.0000000000032462

14. Ishihama Y, Tezuka F, Manabe H, et al. Facet Joint Morphology and Tropism in Adolescents. *Spine (Phila Pa 1976)*. Published online September 4, 2023. doi:10.1097/BRS.0000000000004818

15. Ovcharov ME, Mladenovski MN, Mladenovski IN, Valkov I V., Vasilkova SB. Lumbar disc herniation in children and elderly patients. *Folia Med (Plovdiv)*. 2023;65(4):631-637. doi:10.3897/folmed.65.e97233

16. Zawilla NH, Darweesh H, Mansour N, et al. Matrix Metalloproteinase-3, Vitamin D Receptor Gene Polymorphisms, and Occupational Risk Factors in Lumbar Disc Degeneration. *J Occup Rehabil*. 2014;24(2):370-381. doi:10.1007/s10926-013-9472-7

17. Vinas-Rios JM, Sanchez-Aguilar M, Medina-Govea FA, Meyer F. Early recurrent lumbar disc herniation-data from the German spine registry. *European Spine Journal*. 2017;26(11):2978-3057. doi:10.1007/s00586-017-5336-8

18. Zhu Y, Li S, Niu F, et al. Association between IL4, IL6 gene polymorphism and lumbar disc degeneration in Chinese population. *Oncotarget*. 2017;8(51):89064-89071. doi:10.18632/oncotarget.21650

19. Chen Y, Ma H, Bi D, Qiu B. Association of interleukin 1 gene polymorphism with intervertebral disc degeneration risk in the Chinese Han population. *Biosci Rep*. 2018;38(4). doi:10.1042/BSR20171627

20. Huang WC, Kuo CH, Wu JC, Chen YC. Higher Risk of Intervertebral Disc Herniation among Neurosurgeons Than Neurologists: 15 Year-Follow-Up of a Physician Cohort. *J Clin Med*. 2018;7(8):198. doi:10.3390/jcm7080198

21. Kitis S, Coskun ZM, Tasdemir P, Tuncez E, Zamani AG, Acar A. Analysis of genetic polymorphisms associated with intervertebral disc degeneration. *Cell Mol Biol (Noisy-le-grand)*. 2018;64(10):61-65.

22. Korshøj M, Jørgensen MB, Hallman DM, Lagersted-Olsen J, Holtermann A, Gupta N. Prolonged sitting at work is associated with a favorable time course of low-back pain among blue-collar workers: a prospective study in the DPhacto cohort. *Scand J Work Environ Health*. 2018;44(5):530-538. doi:10.5271/sjweh.3726

23. Zhou X, Cheung CL, Karasugi T, et al. Trans-Ethnic Polygenic Analysis Supports Genetic Overlaps of Lumbar Disc Degeneration With Height, Body Mass Index, and Bone Mineral Density. *Front Genet*. 2018;9. doi:10.3389/fgene.2018.00267

24. Schistad EI, Bjorland S, Røe C, et al. Five-year development of lumbar disc degeneration—a prospective study. *Skeletal Radiol*. 2019;48(6):871-879. doi:10.1007/s00256-018-3062-x

25. Akarirmak U, Sari H. Lumbar disc herniation and vitamin d receptor gene polymorphisms in Turkish patients. *Osteoporosis International*. 2020;31(S1):133-621. doi:10.1007/s00198-020-05696-3

26. Ding Y, Lv S, Li G, Dong S, Sun X, Chen Y. Scheuermann’s disease as a risk factor for lumbar disc herniation recurrence. *J Coll Physicians Surg Pak*. 2020;30(06):584-589. doi:10.29271/jcpsp.2020.06.584

27. Hung IYJ, Shih TTF, Chen BB, Liou SH, Ho IK, Guo YL. The roles of lumbar load thresholds in cumulative lifting exposure to predict disk protrusion in an Asian population. *BMC Musculoskelet Disord*. 2020;21(1):169. doi:10.1186/s12891-020-3167-y

28. Kiraz M, Demir E. Relationship of lumbar disc degeneration with hemoglobin value and smoking. *Neurochirurgie*. 2020;66(5):373-377. doi:10.1016/j.neuchi.2020.06.133

29. Öz T, Kaya İ, Nursal AF, Aydın HE, Demir O, Yiğit S. ACAN Gene VNTR Polymorphism and Intervertebral Disc Degeneration in a Turkish Population. *Medical Bulletin of Haseki*. 2020;58(4):309-314. doi:10.4274/haseki.galenos.2020.6006

30. Wirries N, Schwarze M, Daentzer D, Skutek M. Total hip arthroplasty and lumbar spine disorders: Plain co-existence or mutual influence? *Orthop Rev (Pavia)*. 2020;12(2). doi:10.4081/or.2020.8546

31. Baker HP, Mosenthal W, Qin C, Volchenko E, Athiviraham A. Is average club head speed a risk factor for lower back injuries in professional golfers? A retrospective case control study. *Phys Sportsmed*. 2021;49(2):214-218. doi:10.1080/00913847.2020.1809968

32. Abdallah A, Emel E, Güler Abdallah B. Factors associated with the recurrence of lumbar disk herniation: biomechanical–radiological and demographic factors. *Neurol Res*. 2022;44(9):830-846. doi:10.1080/01616412.2022.2056340

33. Choi TY, Chang MY, Lee SH, Cho JG, Lee S. Psoas muscle measurement as a predictor of recurrent lumbar disc herniation: A retrospective blind study. *Medicine*. 2022;101(26):e29778. doi:10.1097/MD.0000000000029778

34. Guo J, Li G, Ji X, et al. Clinical and Radiological Risk Factors of Early Recurrent Lumbar Disc Herniation at Six Months or Less: A Clinical Retrospective Analysis in One Medical Center. *Pain Physician*. 2022;25(7):E1039-E1045.

35. Iachina M, Ljungdalh P, Nørgård BM, Garvik O, Stenager E, Schiøttz-Christensen B. Psychiatric disorders, diagnosed in psychiatric clinics, in patients with back pain: A cohort study. *Scand J Public Health*. 2023;51(8):1153-1160. doi:10.1177/14034948221100105

36. Kao YC, Chen JY, Chen HH, Liao KW, Huang SS. The association between depression and chronic lower back pain from disc degeneration and herniation of the lumbar spine. *The International Journal of Psychiatry in Medicine*. 2022;57(2):165-177. doi:10.1177/00912174211003760

37. Kasch R, Truthmann J, Hancock MJ, et al. Association of Lumbar MRI Findings with Current and Future Back Pain in a Population-based Cohort Study. *Spine (Phila Pa 1976)*. 2022;47(3):201-211. doi:10.1097/BRS.0000000000004198

38. Konovalov NA, Nazarenko AG, Brinyuk ES, Kaprovoy SV, Beloborodov VA, Stepanov IA. RISK FACTORS FOR RECURRENT LUMBAR DISK HERNIATION. *Coluna/Columna*. 2022;21(4). doi:10.1590/s1808-185120222104263325

39. Ono K, Ohmori K, Yoneyama R, Matsushige O, Majima T. Risk factors and surgical management of recurrent herniation after full-endoscopic lumbar discectomy using interlaminar approach. *J Clin Med*. 2022;11(3):748. doi:10.3390/jcm11030748

40. Siccoli A, Staartjes VE, Klukowska AM, Muizelaar JP, Schröder ML. Overweight and smoking promote recurrent lumbar disk herniation after discectomy. *European Spine Journal*. 2022;31(3):604-613. doi:10.1007/s00586-022-07116-y

41. Thakar S, Raj V, Neelakantan S, et al. Spinal Morphometry As A Novel Predictor For Recurrent Lumbar Disc Herniation Requiring Revision Surgery: Results of A Case Control Study. *Neurol India*. 2022;70(8):211. doi:10.4103/0028-3886.360932

42. Wang F, Chen K, Lin Q, et al. Earlier or heavier spinal loading is more likely to lead to recurrent lumbar disc herniation after percutaneous endoscopic lumbar discectomy. *J Orthop Surg Res*. 2022;17(1):356. doi:10.1186/s13018-022-03242-x

43. Wang X, Liu H, Wang W, et al. Comparison of multifidus degeneration between scoliosis and lumbar disc herniation. *BMC Musculoskelet Disord*. 2022;23(1):891. doi:10.1186/s12891-022-05841-5

44. Abdallah A, Güler Abdallah B. Factors associated with the recurrence of lumbar disk herniation: non-biomechanical–radiological and intraoperative factors. *Neurol Res*. 2023;45(1):11-27. doi:10.1080/01616412.2022.2116525

45. BORJA AJ, CONNOLLY J, KVINT S, et al. Household income is associated with return to surgery following discectomy for far lateral disc herniation. *J Neurosurg Sci*. 2023;67(3). doi:10.23736/S0390-5616.21.05246-2

46. Geere JH, Swamy GN, Hunter PR, et al. Incidence and risk factors for five-year recurrent disc herniation after primary single-level lumbar discectomy. *Bone Joint J*. 2023;105-B(3):315-322. doi:10.1302/0301-620X.105B3.BJJ-2022-1005.R2

47. He H, Ma J, Xiong C, et al. Development and Validation of a Nomogram to Predict the Risk of Lumbar Disk Reherniation within 2 Years After Percutaneous Endoscopic Lumbar Discectomy. *World Neurosurg*. 2023;172:e349-e356. doi:10.1016/j.wneu.2023.01.026

48. Kızılgöz V, Aydın S, Karavaş E, Kantarcı M, Kahraman Ş. Are paraspinal muscle quantity, lumbar indentation value, and subcutaneous fat thickness related to disc degeneration? An MRI-based study. *Radiography*. 2023;29(2):428-435. doi:10.1016/j.radi.2023.02.004

49. Lastra-Power J, Nieves-Ríos C, Baralt-Nazario F, et al. Predictors of reoperation in hispanic-americans with recurrent lumbosacral disc herniation following primary hemilaminectomy and discectomy surgery. *World Neurosurg X*. 2023;18:100172. doi:10.1016/j.wnsx.2023.100172

50. Li X, Pan B, Cheng L, Li G, Liu J, Yuan F. Development and Validation of a Prognostic Model for the Risk of Recurrent Lumbar Disc Herniation After Percutaneous Endoscopic Transforaminal Discectomy. *Pain Physician*. 2023;26(1):81-90.

51. Mäntymäki H, Ponkilainen VT, Huttunen TT, Mattila VM. Regional variations in lumbar spine surgery in Finland. *Arch Orthop Trauma Surg*. 2021;143(3):1451-1458. doi:10.1007/s00402-021-04313-0

52. Monticelli M, Gelmi CAE, Scerrati A, Cavallo MA, De Bonis P. Recurrent or junctional lumbar foraminal herniated disc in patients operated with trans pars microscopic approach. *Neurosurg Rev*. 2023;46(1):211. doi:10.1007/s10143-023-02109-x

53. Shan ZM, Ren XS, Shi H, et al. Machine Learning Prediction Model and Risk Factor Analysis of Reoperation in Recurrent Lumbar Disc Herniation Patients After Percutaneous Endoscopic Lumbar Discectomy. *Global Spine J*. Published online May 10, 2023:219256822311733. doi:10.1177/21925682231173353

54. Zhong D, Wang Y, Lin L, et al. Development and Validation of a Nomogram to Predict the Risk of Recurrent Lower Extremity Radiating Pain Within 1 Week Following Full-Endoscopic Lumbar Discectomy. *World Neurosurg*. 2023;179:e348-e358. doi:10.1016/j.wneu.2023.08.090

55. Zhu F, Jia D, Zhang Y, et al. Moderate to Severe Multifidus Fatty Atrophy is the Risk Factor for Recurrence After Microdiscectomy of Lumbar Disc Herniation. *Neurospine*. 2023;20(2):637-650. doi:10.14245/ns.2346054.027

56. Duran S, Cavusoglu M, Hatipoglu HG, Sozmen Cılız D, Sakman B. Association between Measures of Vertebral Endplate Morphology and Lumbar Intervertebral Disc Degeneration. *Canadian Association of Radiologists Journal*. 2017;68(2):210-216. doi:10.1016/j.carj.2016.11.002

57. Chadha M, Sharma G, Arora SS, Kochar V. Association of facet tropism with lumbar disc herniation. *European Spine Journal*. 2013;22(5):1045-1052. doi:10.1007/s00586-012-2612-5

58. Pan J, Lu X, Yang G, Han Y, Tong X, Wang Y. Lumbar disc degeneration was not related to spine and hip bone mineral densities in Chinese: facet joint osteoarthritis may confound the association. *Arch Osteoporos*. 2017;12(1):20. doi:10.1007/s11657-017-0315-6

59. Papic M, Papic V, Kresoja M, Munteanu V, Mikov I, Cigic T. Relation between grades of intervertebral disc degeneration and occupational activities of patients with lumbar disc herniation. *Vojnosanit Pregl*. 2017;74(12):1121-1127. doi:10.2298/VSP151112306P

60. Shahlaee A, Rahimi-Movaghar V. A familial incidence of L1-L2 disc herniation. *J Neurosurg Sci*. 2016;61(2). doi:10.23736/S0390-5616.16.03262-8

61. Ravindra VM, Senglaub SS, Rattani A, et al. Degenerative lumbar spine disease: estimating global incidence and worldwide volume. *Global Spine J*. 2018;8(8):784-794. doi:10.1177/2192568218770769

62. Reito A, Kyrölä K, Pekkanen L, Paloneva J. Specific spinal pathologies in adult patients with an acute or subacute atraumatic low back pain in the emergency department. *Int Orthop*. 2018;42(12):2843-2849. doi:10.1007/s00264-018-3983-y

63. Läubli R, Brugger R, Pirvu T, et al. Disproportionate Vertebral Bodies and Their Impact on Lumbar Disc Herniation. *J Clin Med*. 2021;10(14):3174. doi:10.3390/jcm10143174

64. Zehra U, Cheung JPY, Bow C, et al. Spinopelvic alignment predicts disc calcification, displacement, and Modic changes: Evidence of an evolutionary etiology for clinically‐relevant spinal phenotypes. *JOR Spine*. 2020;3(1). doi:10.1002/jsp2.1083

65. Walter SS, Lorbeer R, Hefferman G, et al. Correlation between thoracolumbar disc degeneration and anatomical spinopelvic parameters in supine position on MRI. *PLoS One*. 2021;16(6):e0252385. doi:10.1371/journal.pone.0252385

66. Cha E woo, Jung S mi, Lee I ho, et al. Approval status and characteristics of work-related musculoskeletal disorders among Korean workers in 2020. *Ann Occup Environ Med*. 2022;34(1). doi:10.35371/aoem.2022.34.e31

67. Dittmar-Johnson HM, Cruz-López F, González-Camacho E, et al. Prevalence and characteristics of upper lumbar disc herniations in our practice: a retrospective analysis. *Coluna/Columna*. 2022;21(1). doi:10.1590/s1808-185120222101259474

68. Pourabbas Tahvildari B, Masroori Z, Erfani MA, Solooki S, Vosoughi AR. The impact of spino-pelvic parameters on pathogenesis of lumbar disc herniation. *Musculoskelet Surg*. 2022;106(2):195-199. doi:10.1007/s12306-020-00693-5

69. Raymaekers V, Bamps S, Duyvendak W, et al. Real world data collection and cluster analysis in patients with sciatica due to lumbar disc herniation. *Clin Neurol Neurosurg*. 2022;217:107246. doi:10.1016/j.clineuro.2022.107246

70. Azemi ES, Kola S, Kola I, Tanka M, Bilaj F, Abazaj E. Lumbar disk herniation: a clinical epidemiological and radiological evaluation. *Open Access Maced J Med Sci*. 2022;10(B):1588-1594. doi:10.3889/oamjms.2022.8828

71. Tarabeih N, Shalata A, Higla O, Kalinkovich A, Livshits G. The search for systemic biomarkers for monitoring degenerative lumbar spinal disorders. *Osteoarthr Cartil Open*. 2022;4(4):100323. doi:10.1016/j.ocarto.2022.100323

72. Wang P, Chen C, Liu F, et al. The Effects of Ambient Temperature on Lumbar Disc Herniation: A Retrospective Study. *Front Med (Lausanne)*. 2022;9. doi:10.3389/fmed.2022.811237

73. Yazici A, Yerlikaya T. The relationship between the degeneration and asymmetry of the lumbar multifidus and erector spinae muscles in patients with lumbar disc herniation with and without root compression. *J Orthop Surg Res*. 2022;17(1):541. doi:10.1186/s13018-022-03444-3

74. Zhao X, Liang H, Hua Z, et al. The morphological characteristics of paraspinal muscles in young patients with unilateral neurological symptoms of lumbar disc herniation. *BMC Musculoskelet Disord*. 2022;23(1):994. doi:10.1186/s12891-022-05968-5

75. Chen X, Li Y, Wang W, Cui P, Wang Y, Lu S. Correlation between inflammatory cytokine expression in paraspinal tissues and severity of disc degeneration in individuals with lumbar disc herniation. *BMC Musculoskelet Disord*. 2023;24(1):193. doi:10.1186/s12891-023-06295-z

76. Ke S, Sun T, Zhang W, Zhang J, Li Z. Are there correlations between facet joint parameters and lumbar disk herniation laterality in young adults? *Journal of Clinical Neuroscience*. 2023;109:50-56. doi:10.1016/j.jocn.2023.01.013

77. Ordaz A, Anderson B, Zlomislic V, et al. Paraspinal muscle gene expression across different aetiologies in individuals undergoing surgery for lumbar spine pathology. *European Spine Journal*. 2023;32(4):1123-1131. doi:10.1007/s00586-023-07543-5

78. Takahashi M, Iwamoto K, Tomita K, Ueda S, Igawa T, Miyauchi Y. Factors associated with spinal instability in low back lumbar diseases with leg pain: Analysis of sagittal translation and segmental angulation. *J Back Musculoskelet Rehabil*. 2023;36(2):437-444. doi:10.3233/BMR-220067

79. Tian G, Wang Y, Xia J, et al. Correlation of multifidus degeneration with sex, age and side of herniation in patients with lumbar disc herniation. *BMC Musculoskelet Disord*. 2023;24(1):652. doi:10.1186/s12891-023-06783-2
